# Cyanovinylation of Aldehydes: Organocatalytic Multicomponent Synthesis of Conjugated Cyanomethyl Vinyl Ethers

**DOI:** 10.3390/molecules26144120

**Published:** 2021-07-06

**Authors:** Samuel Delgado-Hernández, Fernando García-Tellado, David Tejedor

**Affiliations:** 1 Instituto de Productos Naturales y Agrobiología, Consejo Superior de Investigaciones Científicas, Astrofísico Francisco Sánchez 3, 38206 La Laguna, Spain; samuel050690@gmail.com; 2 Doctoral and Postgraduate School, Universidad de La Laguna, Apartado Postal 456, 38200 La Laguna, Spain

**Keywords:** cyanomethyl vinyl ethers, 3-(cyanomethoxy)acrylates, multicomponent, cyanohydrin, cyanovinylation, organocatalysis, tertiary amine, *N*-methyl morpholine

## Abstract

A novel organocatalytic multicomponent cyanovinylation of aldehydes was designed for the synthesis of conjugated cyanomethyl vinyl ethers. The reaction was implemented for the synthesis of a 3-substituted 3-(cyanomethoxy)acrylates, using aldehydes as substrates, acetone cyanohydrin as the cyanide anion source, and methyl propiolate as the source of the vinyl component. The multicomponent reaction is catalyzed by *N*-methyl morpholine (2.5 mol%) to deliver the 3-(cyanomethoxy)acrylates in excellent yields and with preponderance of the *E*-isomer. The multicomponent reaction manifold is highly tolerant to the structure and composition of the aldehyde (aliphatic, aromatic, heteroaromatics), and it is instrumentally simple (one batch, open atmospheres), economic (2.5 mol% catalyst, stoichiometric reagents), environmentally friendly (no toxic waste), and sustainable (easy scalability).

## 1. Introduction

**Scheme 1:**
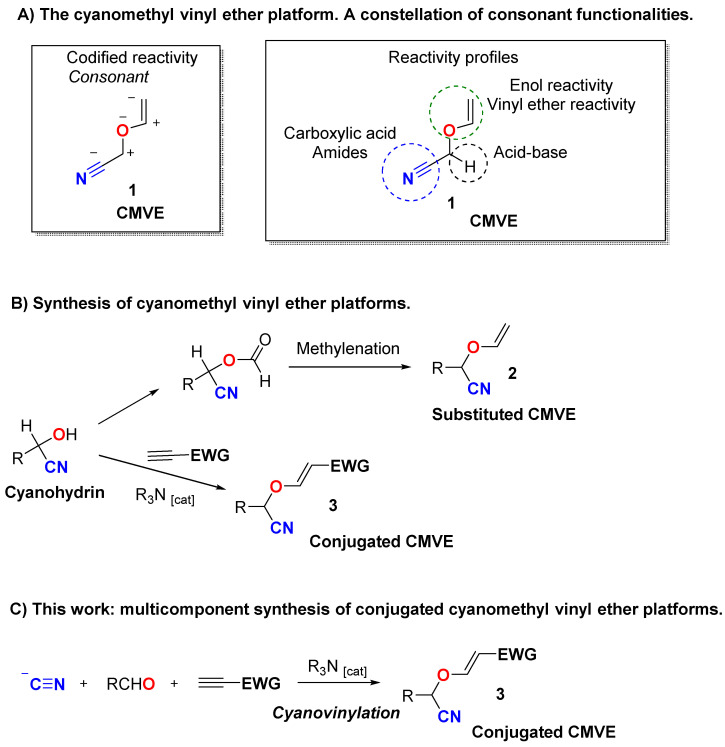
Cyanomethyl vinyl ethers platforms. (**A**) Properties, (**B**) previous synthesis, and (**C**) this work.

Cyanomethyl vinyl ethers (CMVEs) **1** constitute densely functionalized linear synthetic platforms which have found use as monomeric units in the construction of alkenyl ether-vinyl ester copolymers [1,2,3,4,5], as building blocks in the construction of multi-substituted 2,3-dihydrofurans [6,7] and 2,3-dihydropyrroles [7], and as convenient platforms for mechanistic investigations in the Claisen rearrangement of allyl vinyl ethers [8,9,10] (Scheme 1A). They are usually synthesized in a step-wise manner from the corresponding aldehydes through the formation of the *O*-formyl cyanohydrin intermediate and carbonyl methylenation [9] (Scheme 1B). When the vinyl moiety is endowed with electron withdrawing groups (e.g., **3**), the cyanohydrin is directly converted into the conjugated cyanomethyl vinyl ether derivative by the amine-catalyzed Michael addition on the corresponding conjugated alkyne [6,7] (Scheme 1B). In both protocols, the corresponding cyanohydrin has to be synthesized and isolated to be used in the following reaction step. A direct synthesis of CMVEs **1** from the parent aldehydes should be desirable in terms of synthetic efficiency and both labor and step economies. With this idea in mind, we designed the three-component reaction (3CR) depicted in Scheme 1C, which implements a novel cyanovinylation of aldehydes to construct conjugated CMVEs **3**. To the best of our knowledge, this transformation has not been reported in the bibliography [11]. We report herein our results in the design and implementation of this 3CR and its practical application to the synthesis of methyl 3-substituted 3-(cyanomethoxy)acrylates (CMAs) **7**.

## 2. Results and Discussion

### 2.1. Design of a 3CR Manifold for the Synthesis of 3-(Cyanomethoxy)acrylates **7**

**Scheme 2:**
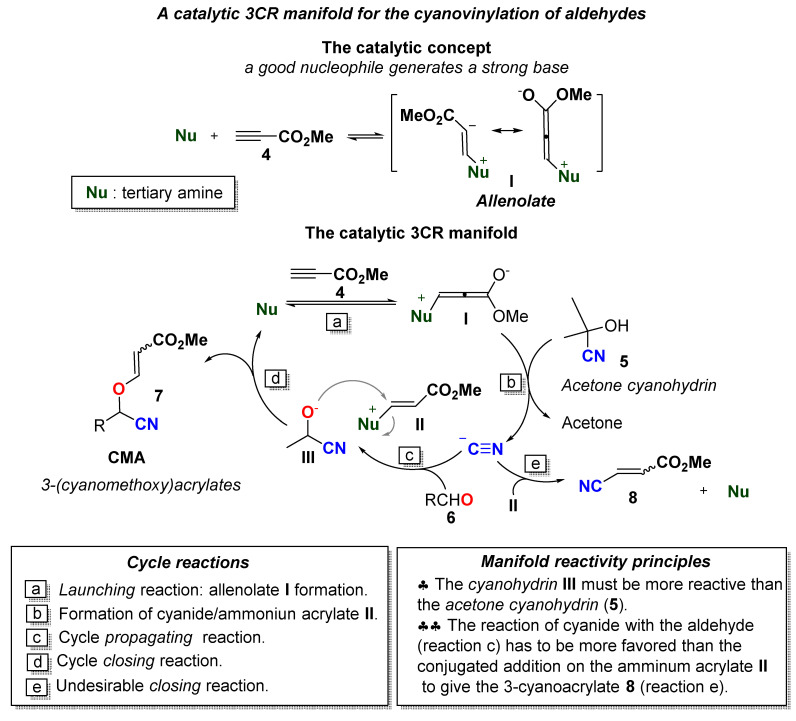
Catalytic 3CR for the synthesis of conjugated 3-(cyanomethoxy)acrylates **7**.

The 3CR process was designed to operate under organocatalytic conditions according to the catalytic concept of “a good nucleophile generates a strong base” [12,13,14,15] (Scheme 2). This catalytic concept allows for launching base-driven processes by the in situ generation of catalytic amounts of a strong base in the reaction medium. It is technically performed by the reaction of a Lewis base (nucleophilic catalyst) on a conjugated alkyne to generate a conjugated vinyl anion (strong base). In the design of this 3CR manifold, we chose a tertiary amine as the Lewis base catalyst, because they have proved to be excellent catalysts for the in situ generation of allenolate anions type **I** [12,16]. With this catalytic principle in mind, we designed the 3CR depicted in Scheme 2. The reaction manifold generates CMAs **7** through the catalytic cyanovinylation of an aldehyde substrate in the presence of stoichiometric amounts of cyanide anion and methyl propiolate (**4**). The choice of the cyanide anion source was an important design issue, because it had to be safe, environmentally friendly and suitable for the generation of cyanide anion under basic conditions, without introducing other reactive species into the catalytic cycle. Among of the commercially available cyanide precursors, we chose acetone cyanohydrin (**5**) because it meets all these criteria: it is safe [17] and it releases cyanide anion and acetone (waste) in the presence of bases [18]. The presence of acetone in the reaction medium should not introduce reactivity distortion issues in the catalytic process.

With all considerations in mind, we envisioned the 3CR depicted in Scheme 2, which would be launched by the addition of the catalyst on the conjugated alkyne to generate the allenolate **I** (Scheme 2, reaction a). Allenolate **I** is a strong base, and it would deprotonate the acetone cyanohydrin (**5**) to generate the cyanide anion and the reactive ammonium acrylate **II** (reaction b). Addition of the cyanide anion on the aldehyde **6** would form the cyanohydrin **III** (reaction c), which would then add onto the ammonium acrylate **II** to deliver the final CMA **7** with liberation of the catalyst to reinitiate the cycle (reaction d). Based on our own experience [12,13,14,16], the catalytic cycle should deliver **7** as an *E/Z* mixture of isomers, with a clear preponderance of the *E*-isomer.

A critical concern in this design arises from the reactivity of the cyanide anion toward the aldehyde **6** versus the ammonium acrylate **II** (step c). The catalytic cycle requires that the cyanide anion fully react with the aldehyde to avoid the undesirable formation of methyl 3-cyanoacrylate **8** (reaction e) [12]. It must be noted that the formation of methyl 3-cyanoacrylate **8** is also catalytic: its formation releases the catalyst to reinitiate the cycle [12]. Gratifyingly, the reactivity of the cyanide anion toward the aldehyde was good enough to mostly funnel the catalytic manifold toward formation of the CMA **7**.

### 2.2. Implementation of the 3CR Manifold

We chose the reaction of benzaldehyde (**6a**), acetone cyanohydrin (**5**) and methyl propiolate (**4**) as the benchmark reaction to find a set of convenient reaction conditions for the 3CR (Table 1). A wide panel of different tertiary amines and solvents was used in this exploratory study. Among all the combinations of tertiary amines and solvents assayed (entries 1–18), the combination of *N*-methylmorpholine (NMM) and *n*-hexanes showed to be the best, funneling the 3CR toward a nearly quantitative production of CMA **7a** (99%, *E*/*Z*:3/1) (only traces of 3-cyanoacrylate **8** were observed) (entry 8). More importantly, the NMM charge could be reduced up to 2.5 mol% without significant loss in efficiency (94%) and stereoselectivity (2.5/1) (entry 21). The effect of temperature on the stereoselectivity was studied carrying out the reaction at 0 °C and −78 °C, without observing any appreciable improvement at both temperatures (entries 22 and 23). As expected, no reaction was observed in the absence of catalyst (entry 24). Thus, we chose the conditions depicted in entry 21 as the standard reaction conditions: *N*-methyl morpholine (2.5 mol%), *n*-hexanes, room temperature.

### 2.3. Scope of the 3CR Manifold

**Scheme 3:**
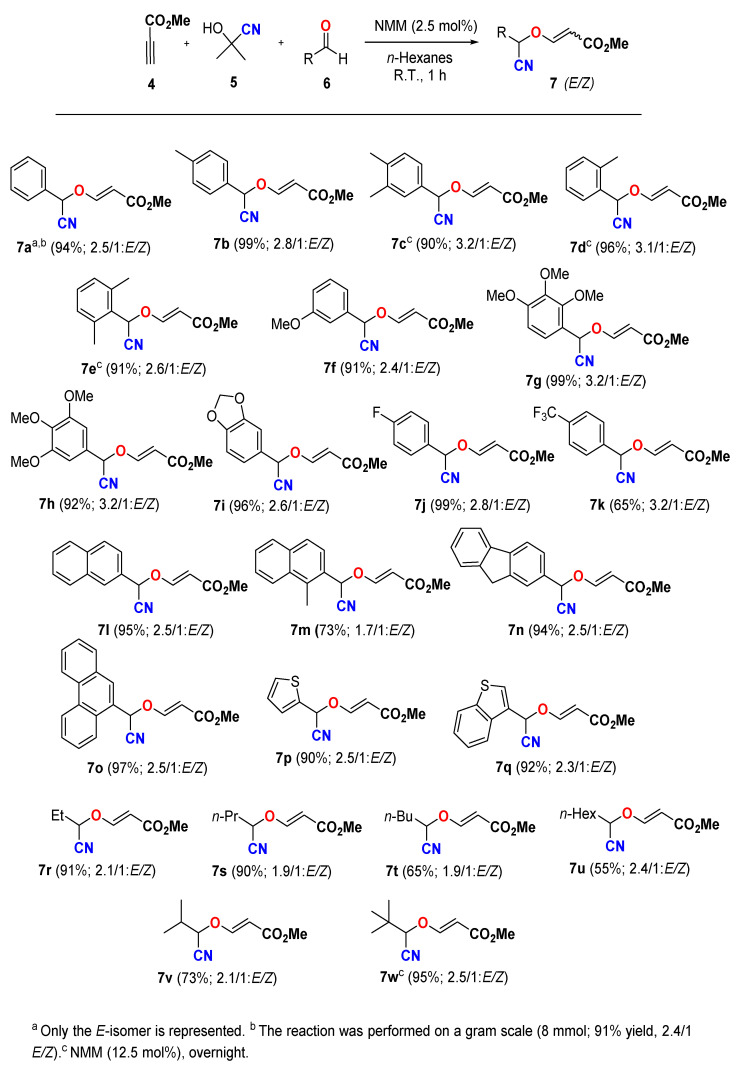
Aldehyde scope of the 3CR.

The scope of the catalytic 3CR was studied using a wide array of different aldehydes **6** and the standardized reaction conditions (Scheme 3). Aldehydes were chosen to span a wide reactivity profile, including aromatic (**6a**–**o**) heteroaromatic (**6p**–**q**) and aliphatic (**6r**–**w**). The reaction process was widely tolerant with respect to the aldehyde nature delivering the corresponding CMAs **7a**–**w** in yields spanning from 55% (**7u**) to 99% (**7b**, **7g** and **7j**).

Although the electronic nature of the aromatic rings did not show a general influence in the efficiency of the 3CR (compare **7b**, **7g** and **7j**), the steric environment of the carbonyl function of the aldehyde was more determining, requiring, in the most steric-demanding cases, the use of higher charges of catalyst (12.5 mol%) and longer reaction times (overnight) to achieve good yields of CMAs (see **7c**, **7d**, **7e** and **7w**). Under the standard reaction conditions, the hindered 1-methyl-2-naphthaldehyde (**6m**) and isobutanal (**6v**) delivered the corresponding **7m** and **7v** in good yields (73%), and their reactions were not further implemented. The reactivity of the linear aliphatic aldehydes was dependent on the length of the chain, being optimal for 2–3 carbon atoms (**7r**, **7s**) and disadvantageous for longer chains (**7t**, **7u**). With regard to the stereoselectivity of the reaction, the *E*/*Z* ratio was moderate in all cases, spanning from 1.7/1 for **7m** to 3.2/1 for **7c**, **7g**, **7h** and **7k**.

Additionally, the scaling power of this 3CR and its suitability for real-world synthesis was established performing the reaction of benzaldehyde (**6a**) on a gram-scale (8 mmol). Under these conditions, CMA **7a** was obtained in 91% yield as a mixture of *E/Z* isomers (2.4/1).

Finally, and in accordance with our previous findings on the use of tertiary amines as catalysts for domino processes involving activated alkynes [13,16,19], the use of internal alkynes such as methyl 2-octynoate or ethyl phenylpropiolate did not lead to the formation of products.

## 3. Materials and Methods

### 3.1. General Information

^1^H NMR and ^13^C NMR spectra of CDCl_3_ solutions were recorded either at 400 and 100 MHz or at 500 and 125 MHz (Bruker Ac 200 and AMX2-500 respectively). ^1^H and ^13^C NMR spectra for compounds **7** are available from the Supplementary Materials. High-resolution mass spectra were recorded with a mass spectrometer LCT Premier XE (Manchester, UK) with two types of ionization sources: electrospray (ESI), an atmospheric pressure chemical ionization source (APCI), and with an orthogonal acceleration time-offlight (oa-TOF) analyzer. Analytical thin-layer chromatography plates used were E. Merck Brinkman UV-active silica gel (Kieselgel 60 F254) on aluminum. Flash column chromatography was carried out with E. Merck silica gel 60 (particle size less than 0.020 mm) using appropriate mixtures of ethyl acetate and hexanes, or ethyl acetate and dichloromethane as eluents. All reactions were performed in oven-dried glassware. All materials were obtained from commercial suppliers and used as received.

### 3.2. General Procedure for the Synthesis of Cyanomethyl Vinyl Ether **7a**

To a solution of aldehyde (**6**) (2.0 mmol), acetone cyanohydrin (**5**) (2.0 mmol) and methyl propiolate (**4**) (2.0 mmol) in *n*-hexanes (6 mL) was added *N*-methylmorpholine (0.05 mmol) at once and the reaction mixture was stirred for 1 h at room temperature. The solvent was removed under reduced pressure, and the residue was purified by flash column chromatography (silica gel; *n*-hexane/ethyl acetate: 80/20) to give the desired 3-(cyanomethoxy)acrylate **7**.

### 3.3. Characterization Data for 3-(cyanomethoxy)acrylates **7**

*Methyl 3-(cyano(phenyl)methoxy)acrylate (***7a***). (E*-isomer, major): (8.0 mol scale) (1.12 g, 64.5%). White solid: ^1^H NMR (CDCl_3_, 400 MHz): δ = 3.71 (s, 3H), 5.53 (d, 1H, *J* = 12.6 Hz), 5.65 (s, 1H), 7.46–7.53 (m, 5H), 7.56 (d, 1H, *J* = 12.6 Hz). ^13^C NMR (CDCl_3_, 100 MHz): δ = 51.5, 70.3, 101.3, 115.2, 127.4 (2C), 129.4 (2C), 130.7, 131.2, 158.1, 166.7 ppm. HRMS (ESI^+^): *m*/*z* [M + Na]^+^ calculated for C_12_H_11_NO_3_Na 240.0636, found 240.0637. *(Z*-isomer, minor): (8.0 mol scale) (466 mg, 26.8%). Yellowish solid: ^1^H NMR (CDCl_3_, 400 MHz): δ = 3.69 (s, 3H), 5.09 (d, 1H, *J* = 7.1 Hz), 5.83 (s, 1H), 6.59 (d, 1H, *J* = 7.1 Hz), 7.45–7.46 (m, 3H), 7.53–7.55 (m, 2H). ^13^C NMR (CDCl_3_, 100 MHz): δ = 51.2, 71.9, 101.0, 115.6, 127.3 (2C), 129.4 (2C), 130.6, 131.3, 152.7, 164.5 ppm.

*Methyl 3-(cyano(p-tolyl)methoxy)acrylate (***7b***)*. *(E*-isomer, major): (341.2 mg, 74%). White solid: ^1^H NMR (CDCl_3_, 400 MHz): δ = 2.39 (s, 3H), 3.71 (s, 3H), 5.51 (d, 1H, *J* = 12.7 Hz), 5.62 (s, 1H), 7.27 (d, 2H, *J* = 8.1 Hz), 7.38 (d, 2H, *J* = 8.1 Hz), 7.54 (d, 1H, *J* = 12.7 Hz). ^13^C NMR (CDCl_3_, 100 MHz): δ = 21.2, 51.3, 70.2, 101.1, 114.8 (2C), 115.4, 127.4 (2C), 128.2, 130.0 (2C), 141.0, 158.1, 166.7 ppm. HRMS (ESI^+^): *m*/*z* [M + Na]^+^ calculated for C_13_H_13_NO_3_Na 254.0793, found 254.0791. *(Z*-isomer, minor): (120.0 mg, 26%). Yellow oil: ^1^H NMR (CDCl_3_, 400 MHz): δ = 2.32 (s, 3H), 3.64 (s, 3H), 5.01 (d, 1H, *J* = 7.2 Hz), 5.76 (s, 1H), 6.53 (d, 1H, *J* = 7.2 Hz), 7.20 (d, 2H, *J* = 8.1 Hz), 7.37 (d, 2H, *J* = 8.1 Hz). ^13^C NMR (CDCl_3_, 100 MHz): δ = 21.2, 51.1, 71.8, 101.6, 115.7 (2C), 127.4 (2C), 128.4, 129.9 (2C), 125.8, 140.9, 164.5 ppm.

*Methyl 3-(cyano(3,4-dimethylphenyl)methoxy)acrylate (***7c***). (E*-isomer, major): (337.0 mg, 69%). White solid: ^1^H NMR (CDCl_3_, 500 MHz): δ = 2.25 (s, 3H), 2.27 (s, 3H), 3.67 (s, 3H), 5.47 (d, 1H, *J* = 12.5 Hz), 5.55 (s, 1H), 7.19 (s, 2H), 7.23 (s, 1H), 7.27 (d, 2H, *J* = 8.1 Hz), 7.38 (d, 2H, *J* = 8.1 Hz), 7.50 (d, 1H, *J* = 12.5 Hz). ^13^C NMR (CDCl_3_, 125 MHz): δ = 19.6, 19.7, 51.4, 70.3, 100.9, 115.5, 125.0, 128.5, 128.6, 130.5, 138.0, 139.7 158.2, 166.9 ppm. HRMS (ESI^+^): *m*/*z* [M + Na]^+^ calculated for C_14_H_15_NO_3_Na 268.0950, found 268.0954. (*Z*-isomer, minor): (104.5 mg, 21%). Transparent oil: ^1^H NMR (CDCl_3_, 500 MHz): δ = 2.24 (s, 3H), 2.25 (s, 3H), 3.65 (s, 3H), 5.01 (d, 1H, *J* = 7.1 Hz), 5.74 (s, 1H), 6.55 (d, 1H, *J* = 7.1 Hz), 7.17 (d, 1H, *J* = 8.0 Hz), 7.22 (d, 1H, *J* = 8.0 Hz), 7.26 (s, 1H). ^13^C NMR (CDCl_3_, 125 MHz): δ = 19.5, 19.7, 51.1, 71.8, 100.3, 115.8, 125.0, 128.5, 128.6, 130.4, 137.9, 139.6, 152.9, 164.7 ppm.

*Methyl 3-(cyano(o-tolyl)methoxy)acrylate (***7d***)*. (*E*-isomer, major): (340.0 mg, 72%). White solid: ^1^H NMR (CDCl_3_, 500 MHz): δ = 2.40 (s, 3H), 3.71 (s, 3H), 5.55 (d, 1H, ^3^*J*(H,H) = 12.4 Hz), 5.81 (s, 1H), 7.26–7.32 (m, 2H), 7.38 (t,1H, *J* = 7.5 Hz), 7.57 (d, 1H, *J* = 7.5 Hz), 7.59 (d, 1H, *J* = 12.4 Hz). ^13^C NMR (CDCl_3_, 125 MHz): δ = 18.6, 51.3, 68.6, 100.9, 115.1, 126.7, 128.0, 129.1, 130.7, 131.4, 136.6, 158.0, 166.6 ppm. HRMS (ESI^+^): *m*/*z* [M + Na]^+^ calculated for C_13_H_13_NO_3_Na 254.0793, found 254.0796. (*Z*-isomer, minor): (110.0 mg, 24%). Transparent oil: ^1^H NMR (CDCl_3_, 500 MHz): δ = 2.46 (s, 3H), 3.68 (s, 3H), 5.06 (d, 1H, *J* = 7.1 Hz), 5.88 (s, 1H), 6.60 (d, 1H, *J* = 7.1 Hz), 7.25–7.30 (m, 2H), 7.36 (t, 1H, *J* = 7.5 Hz), 7.59 (d, 1H, *J* = 7.5 Hz). ^13^C NMR (CDCl_3_, 125 MHz): δ = 18.8, 51.3, 70.8, 100.6, 115.4, 126.6, 128.2, 129.3, 130.8, 131.5, 137.1, 152.8, 164.5 ppm.

*Methyl 3-(cyano(2,6-dimethylphenyl)methoxy)acrylate (***7e***)* (*E*-isomer, major): (319.6 mg, 65%). Transparent solid: ^1^H NMR (CDCl_3_, 400 MHz): δ = 2.48 (s, 6H), 3.70 (s, 3H), 5.52 (d, 1H, *J* = 12.4 Hz), 5.97 (s, 1H), 7.09 (d, 2H, *J* = 7.5 Hz), 7.23 (t, 1H, *J* = 7.5 Hz), 7.50 (d, 1H, *J* = 12.4 Hz). ^13^C NMR (CDCl_3_, 100 MHz): δ = 20.0 (2C), 51.1, 65.8, 100.7, 115.1, 127.8, 129.6 (2C), 130.6, 137.6 (2C), 158.2, 166.8 ppm. HRMS (ESI^+^): *m*/*z* [M + Na]^+^ calculated for C_14_H_15_NO_3_Na 268.0950, found 268.0950. (*Z*-isomer, minor): (125.1 mg, 26%). Transparent oil: ^1^H NMR (CDCl_3_, 500 MHz): δ = 2.54 (s, 6H), 3.69 (s, 3H), 5.05 (d, 1H, *J* = 6.9 Hz), 6.00 (s, 1H), 6.54 (d, 1H, *J* = 6.9 Hz), 7.08 (d, 2H, *J* = 7.6 Hz), 7.22 (t, 1H, *J* = 7.6 Hz). ^13^C NMR (CDCl_3_, 125 MHz): δ = 20.0 (2C), 51.1, 68.2, 100.2, 115.6, 128.1, 129.5 (2C), 130.5, 137.7 (2C), 153.6, 164.5 ppm.

*Methyl 3-(cyano(4-methoxyphenyl)methoxy)acrylate (***7f***)* (*E*-isomer, major): (316.8 mg, 64%). White solid: ^1^H NMR (CDCl_3_, 400 MHz): δ = 3.71 (s, 3H), 3.81 (s, 3H), 5.50 (d, 1H, *J* = 12.6 Hz), 5.58 (s, 1H), 6.97 (d, 1H, *J* = 8.7 Hz), 7.42 (d, 1H, *J* = 8.7 Hz), 7.52 (d, 1H, *J* = 12.6 Hz). ^13^C NMR (CDCl_3_, 100 MHz): δ = 51.4, 55.4, 70.1, 101.2, 114.8 (2C), 115.4, 123.2, 129.3 (2C), 158.0, 161.5, 166.8 ppm. HRMS (ESI^+^): *m*/*z* [M + Na]^+^ calculated for C_13_H_13_NO_4_Na 270.0742, found 270.0743. (*Z*-isomer, minor): (130.9 mg, 27%). Yellow solid: ^1^H NMR (CDCl_3_, 400 MHz): δ = 3.68 (s, 3H), 3.81 (s, 3H), 5.05 (d, 1H, *J* = 7.1 Hz), 5.77 (s, 1H), 6.57 (d, 1H, *J* = 7.1 Hz), 6.94 (d, 1H, *J* = 8.7 Hz), 7.45 (d, 1H, *J* = 8.7 Hz). ^13^C NMR (CDCl_3_, 100 MHz): δ = 51.1, 55.4, 71.6, 100.5, 114.7 (2C), 115.8, 123.3, 129.2 (2C), 152.7, 161.3, 164.5 ppm.

*Methyl 3-(cyano(2,3,4-trimethoxyphenyl)methoxy)acrylate* (**7g**) (*E*-isomer, major): (467.1 mg, 76%). Transparent oil: ^1^H NMR (CDCl_3_, 400 MHz): δ = 3.65 (s, 3H), 3.81 (s, 3H), 3.84 (s, 3H), 3.92 (s, 3H), 5.46 (d, 1H, *J* = 12.5 Hz), 5.88 (s, 1H), 6.69 (d, 1H, *J* = 8.6 Hz), 7.21 (d, 1H, *J* = 8.6 Hz), 7.52 (d, 1H, *J* = 12.5 Hz). ^13^C NMR (CDCl_3_, 100 MHz): δ = 51.1, 55.9, 60.6, 61.2, 65.9, 100.4, 115.6, 117.2, 123.3, 141.8, 151.4, 156.1, 158.5, 166.7 ppm. HRMS (ESI^+^): *m*/*z* [M + Na]^+^ calculated for C_15_H_17_NO_6_Na 330.0954, found 330.0951. (*Z*-isomer, minor): (147.8 mg, 24%). Yellow oil: ^1^H NMR (CDCl_3_, 400 MHz): δ = 3.68 (s, 3H), 3.86 (s, 3H), 3.89 (s, 3H), 3.98 (s, 3H), 5.05 (d, 1H, *J* = 7.05 Hz), 5.97 (s, 1H), 6.67 (d, 1H, *J* = 7.05 Hz), 6.73 (d, 1H, *J* = 8.52 Hz), 7.32 (d, 1H, *J* = 8.52 Hz). ^13^C NMR (CDCl_3_, 100 MHz): δ = 51.1, 56.1, 60.8, 61.5, 68.1, 99.9, 107.4, 116.0, 117.6, 123.5, 142.1, 151.6, 153.6, 156.1, 164.6 ppm.

*Methyl 3-(cyano(3,4,5-trimethoxyphenyl)methoxy)acrylate* (**7h**) (*E*-isomer, major): (428.4 mg, 70%). Yellow oil: ^1^H NMR (CDCl_3_, 400 MHz): δ = 3.67 (s, 3H), 3.82 (s, 3H), 3.84 (s, 6H), 5.50 (d, 1H, *J* = 12.8 Hz), 5.60 (s, 1H), 6.67 (s, 2H), 7.51 (d, 1H, *J* = 12.8 Hz). ^13^C NMR (CDCl_3_, 100 MHz): δ = 51.3, 56.1 (2C), 60.7, 70.2, 101.1, 104.6 (2C), 115.2, 126.3, 139.7, 153.8 (2C), 157.9, 166.6 ppm. HRMS (ESI^+^): *m*/*z* [M + Na]^+^ calculated for C_15_H_17_NO_6_Na 330.0954, found 330.0951. (*Z*-isomer, minor): (135.1 mg, 22%). Yellow solid: ^1^H NMR (CDCl_3_, 400 MHz): δ = 3.68 (s, 3H), 3.83 (s, 3H), 3.86 (s, 6H), 5.10 (d, 1H, *J* = 7.1 Hz), 5.77 (s, 1H), 6.58 (d, 1H, *J* = 7.1 Hz), 6.75 (s, 2H). ^13^C NMR (CDCl_3_, 100 MHz): δ = 51.1, 56.2 (2C), 60.7, 71.9, 100.9, 104.5 (2C), 115.6, 126.6, 139.6, 152.7 (2C), 153.9, 164.5 ppm.

*Methyl 3-(benzo[d]*[1,3]*dioxol-5-yl(cyano)methoxy)acrylate* (**7i**). (*E*-isomer, major): (361.5 mg, 69%). Orange solid: ^1^H NMR (CDCl_3_, 500 MHz): δ = 3.70 (s, 3H), 5.49 (d, 1H, *J* = 12.5 Hz), 5.55 (s, 1H), 6.01 (s, 2H), 6.84 (d, 1H, *J* = 8.0 Hz), 6.94 (s, 1H), 6.97 (d, 1H, *J* = 8.0 Hz), 7.50 (d, 1H, *J* = 12.5 Hz). ^13^C NMR (CDCl_3_, 125 MHz): δ = 51.4, 70.1, 101.2, 101.9, 107.7, 108.7, 115.3, 122.0, 124.7, 148.6, 149.7, 157.9, 166.7 ppm. HRMS (ESI^+^): *m*/*z* [M + Na]^+^ calculated for C_13_H_11_NO_5_Na 284.0535, found 284.0530. (*Z*-isomer, minor): (140.7 mg, 27%). Yellow solid: ^1^H NMR (CDCl_3_, 500 MHz): δ = 3.69 (s, 3H), 5.08 (d, 1H, *J* = 7.1 Hz), 5.72 (s, 1H), 6.01 (s, 2H), 6.57 (d, 1H, *J* = 7.1 Hz), 6.84 (d, 1H, *J* = 8.0 Hz), 7.00 (s,1H), 7.02 (d, 1H, *J* = 8.0 Hz). ^13^C NMR (CDCl_3_, 125 MHz): δ = 51.2, 71.7, 100.7, 101.8, 107.7, 108.6, 115.6, 121.9, 124.9, 148.7, 149.6, 152.6, 164.5 ppm.

*Methyl 3-(cyano(4-fluorophenyl)methoxy)acrylate* (**7j**) (*E*-isomer, major): (345.0 mg, 73%). Transparent oil: ^1^H NMR (CDCl_3_, 500 MHz): δ = 3.69 (s, 3H), 5.12 (d, 1H, *J* = 12.5 Hz), 5.67 (s, 1H), 7.14 (t, 2H, *J* = 8.5 Hz), 7.48–7.51 (m, 2H), 7.53 (d, 1H, *J* = 12.5 Hz). ^13^C NMR (CDCl_3_, 125 MHz): δ = 51.5, 69.4, 101.3, 112.2, 116.5 (d, 2C, *J* = 22.6 Hz), 127.2 (d, *J* = 3.2 Hz), 129.6 (d, 2C, JCF = 8.9 Hz), 157.9, 163.8 (d, JCF = 250.8 Hz), 166.6 ppm. HRMS (ESI^+^): *m*/*z* [M + Na]^+^ calculated for C_12_H_10_FNO_3_Na 258.0542, found 258.0547. (*Z*-isomer, minor): (125.0 mg, 27%). Yellow oil: ^1^H NMR (CDCl_3_, 500 MHz): δ = 3.69 (s, 3H), 5.12 (d, 1H, *J* = 7.1 Hz), 5.81 (s, 1H), 6.59 (d, 1H, *J* = 7.1 Hz), 7.14 (t, 2H, *J* = 7.9 Hz), 7.55 (t, 2H, *J* = 7.9 Hz). ^13^C NMR (CDCl_3_, 125 MHz): δ = 51.2, 71.2, 101.1, 115.5, 116.5 (d, 2C, JCF = 22.2 Hz), 127.3 (d, *J* = 2.8 Hz), 129.4 (d, 2C, *J* = 8.8 Hz), 152.7, 163.8 (d, *J* = 251.0 Hz), 164.4 ppm.

*Methyl 3-(cyano(4-(trifluoromethyl)phenyl)methoxy)acrylate* (**7k**). (*E*-isomer, major): (283.2 mg, 50%). White solid: ^1^H NMR (CDCl_3_, 400 MHz): δ = 3.72 (s, 3H), 5.55 (d, 1H, *J* = 12.5 Hz), 5.76 (s, 1H), 7.57 (d, 1H, *J* = 12.5 Hz), 7.65 (d, 2H, *J* = 8.3 Hz), 7.74 (d, 2H, *J* = 8.3 Hz). ^13^C NMR (CDCl_3_, 100 MHz): δ = 51.5, 69.3, 101.7, 114.7, 123.4 (q, *J* = 270.3 Hz), 126.4 (q, 2C, *J* = 3.6 Hz), 127.7 (2C), 132.8 (q, *J* = 33.5 Hz), 134.9, 157.8, 166.5 ppm. HRMS (ESI^+^): *m*/*z* [M + Na]^+^ calculated for C_13_H_10_F_3_NO_3_Na 308.0510, found 308.0510. (*Z*-isomer, minor): (88.0 mg, 15%). Yellow solid: ^1^H NMR (CDCl_3_, 400 MHz): δ = 3.71 (s, 3H), 5.18 (d, 1H, *J* = 6.9 Hz), 5.89 (s, 1H), 6.62 (d, 1H, *J* = 6.9 Hz), 7.73 (dd, 4H, *J* = 8.6 Hz and 4.6 Hz). ^13^C NMR (CDCl_3_, 100 MHz): δ = 51.2, 71.2, 101.8, 115.1, 123.4 (q, *J* = 273.8 Hz), 126.4 (q, 2C, *J* = 3.9 Hz), 127.5 (2C), 132.6 (q, *J* = 33.1 Hz), 135.2, 152.5, 164.3 ppm.

*Methyl 3-(cyano(naphthalen-2-yl)methoxy)acrylate* (**7l**). (*E*-isomer, major): (360.9 mg, 67%). White solid: ^1^H NMR (CDCl_3_, 400 MHz): δ = 3.71 (s, 3H), 5.57 (d, 1H, *J*= 12.6 Hz), 5.81 (s, 1H), 7.56 (d, 1H, *J* = 8.6 Hz), 7.55–7.58 (m, 2H), 7.60 (d, 1H, *J* = 12.6 Hz), 7.86–7.89 (m, 2H), 7.92 (d, 1H, *J* = 8.6 Hz), 7.99 (s, 1H). ^13^C NMR (CDCl_3_, 100 MHz): δ = 51.4, 70.5, 101.4, 115.3, 123.6, 127.2, 127.6, 127.7, 127.8, 128.3, 128.4, 129.6, 132.7, 133.9, 158.0, 166.7 ppm. HRMS (ESI^+^): *m*/*z* [M + Na]^+^ calculated for C_16_H_13_NO_3_Na 290.0793, found 290.0794. (*Z*-isomer, minor): (147.6 mg, 28%). Yellow solid: ^1^H NMR (CDCl_3_, 400 MHz): δ = 3.71 (s, 3H), 5.09 (d, 1H, *J* = 7.0 Hz), 6.00 (s, 1H), 6.64 (d, 1H, *J* = 7.0 Hz), 7.55–7.58 (m, 3H), 7.84–7.89 (m, 2H), 7.91 (d, 1H, *J* = 8.6 Hz), 8.05 (s, 1H). ^13^C NMR (CDCl_3_, 100 MHz): δ = 51.1, 72.1, 100.9, 115.6, 123.7, 127.1, 127.4, 127.6, 127.7, 128.4, 128.5, 129.7, 132.7, 133.9, 152.7, 164.5 ppm.

*Methyl 3-(cyano(3-methylnaphthalen-2-yl)methoxy)acrylate* (**7m**). (*E*-isomer, major): (233.8 mg, 46%). White solid: ^1^H NMR (CDCl_3_, 500 MHz): δ = 2.66 (s, 3H), 3.71 (s, 3H), 5.58 (d, 1H, *J* = 12.4 Hz), 6.40 (s, 1H), 7.33 (d, 1H, *J* = 8.4 Hz), 7.52 (t, 1H, *J* = 7.9 Hz), 7.57 (d, 1H, *J* = 12.4 Hz), 7.62 (t, 1H, *J* = 7.9 Hz), 7.87 (d, 2H, *J* = 8.4 Hz), 8.22 (d, 1H, *J* = 8.4 Hz). ^13^C NMR (CDCl_3_, 125 MHz): δ = 20.5, 51.4, 65.5, 100.9, 115.5, 123.1, 123.8, 125.9, 127.6, 128.0, 129.0, 130.6, 131.6, 132.8, 136.2, 158.2, 166.8 ppm. HRMS (ESI^+^): *m*/*z* [M + Na]^+^ calculated for C_17_H_15_NO_3_Na 304.0950, found 304.0955. (*Z*-isomer, minor): (137.6 mg, 27%). Brown solid: ^1^H NMR (CDCl_3_, 500 MHz): δ = 2.70 (s, 3H), 3.69 (s, 3H), 5.03 (d, 1H, *J* = 7.0 Hz), 6.44 (s, 1H), 6.56 (d, 1H, *J* = 7.0 Hz), 7.34 (d, 1H, *J* = 8.4 Hz), 7.52 (t, 1H, *J* = 7.4 Hz), 7.64 (t, 1H, *J* = 7.4 Hz), 7.86 (d, 2H, *J* = 8.4 Hz), 8.34 (d, 1H, *J* = 8.4 Hz). ^13^C NMR (CDCl_3_, 125 MHz): δ = 20.5, 51.3, 67.7, 100.8, 116.0, 123.4, 124.1, 125.9, 127.7, 128.9, 129.1, 130.8, 131.6, 132.9, 136.6, 153.2, 164.6 ppm.

*Methyl 3-(cyano(9H-fluoren-2-yl)methoxy)acrylate* (**7n**). (*E*-isomer, major): (412.1 mg, 67%). Yellow solid: ^1^H NMR (CDCl_3_, 500 MHz): δ = 3.72 (s, 3H), 3.92 (s, 2H), 5.55 (d, 1H, *J* = 12.5 Hz), 5.70 (s, 1H), 7.35–7.42 (m, 2H), 7.49 (d, 1H, *J* = 7.5 Hz), 7.57 (d, 1H, *J* = 7.5 Hz), 7.59 (d, 1H, *J* = 12.5 Hz), 7.68 (s, 1H), 7.80–7.84 (m, 2H). ^13^C NMR (CDCl_3_, 125 MHz): δ = 36.7, 51.4, 70.5, 101.1, 115.5, 120.4, 120.5, 124.1, 125.1, 126.4, 126.9, 127.7, 129.0, 140.1, 143.5, 144.1, 144.2, 158.2, 166.7 ppm. HRMS (ESI^+^): *m*/*z* [M + Na]^+^ calculated for C_19_H_15_NO_3_Na 328.0950, found 328.0951. (*Z*-isomer, minor): (162.2 mg, 27%). Orange solid: ^1^H NMR (CDCl_3_, 500 MHz): δ = 3.72 (s, 3H), 3.94 (s, 2H), 5.10 (d, 1H, *J* = 7.1 Hz), 5.90 (s, 1H), 6.65 (d, 1H, *J* = 7.1 Hz), 7.35–7.42 (m, 2H), 7.56 (t, 2H, *J* = 7.6 Hz), 7.74 (s, 1H), 7.81 (d, 1H, *J* = 7.6 Hz), 7.84 (d, 1H, *J* = 7.6 Hz). ^13^C NMR (CDCl_3_, 125 MHz): δ = 36.9, 51.2, 72.2, 100.8, 115.8, 120.4, 120.5, 124.2, 125.2, 126.4, 127.0, 127.8, 129.3, 140.3, 143.6, 144.2, 144.3, 152.8, 164.6 ppm.

*Methyl 3-(cyano(phenanthren-9-yl)methoxy)acrylate* (**7o**). (*E*-isomer, major): (434.8 mg, 69%). White solid: ^1^H NMR (CDCl_3_, 500 MHz): δ = 3.71 (s, 3H), 5.54 (d, 1H, *J* = 12.5 Hz), 6.25 (s, 1H), 7.35–7.42 (m, 4H), 7.66 (d, 1H, *J* = 12.5 Hz), 7.91–7.94 (m, 2H), 8.07 (s, 1H), 8.64 (d, 1H, *J* = 8.4 Hz), 8.71 (d, 1H, *J* = 8.4 Hz). ^13^C NMR (CDCl_3_, 125 MHz): δ = 51.4, 69.4, 101.5, 115.1, 122.6, 123.3, 123.5, 124.6, 127.4, 127.5, 127.6, 127.8, 128.7, 129.4, 129.5, 129.9, 130.6, 131.3, 157.6, 166.7 ppm. HRMS (ESI^+^): *m*/*z* [M + Na]^+^ calculated for C_20_H_15_NO_3_Na 340.0950, found 340.0946. (*Z*-isomer, minor): (177.2 mg, 28%). White solid: ^1^H NMR (CDCl_3_, 500 MHz): δ = 3.68 (s, 3H), 5.03 (d, 1H, *J* = 7.1 Hz), 6.39 (s, 1H), 6.68 (d, 1H, *J* =7.1 Hz), 7.62 (t, 1H, *J* = 6.9 Hz), 7.65–7.72 (m, 3H), 7.90 (d, 1H, *J* = 7.9 Hz), 8.08 (s, 1H), 8.10 (d, 1H, *J* = 6.9 Hz), 8.60 (d, 1H, *J* = 8.4 Hz), 8.66 (d, 1H, *J* = 8.4 Hz). ^13^C NMR (CDCl_3_, 125 MHz): δ = 51.1, 71.1, 100.8, 115.5, 122.5, 123.3, 123.8, 124.8, 127.3, 127.5, 127.6, 127.8, 128.6, 129.4, 129.8, 129.8, 130.8, 131.3, 151.9, 164.4 ppm.

*Methyl 3-(cyano(thiophen-2-yl)methoxy)acrylate* (**7p**). (*E*-isomer, major): (282.0 mg, 64%). Orange oil: ^1^H NMR (CDCl_3_, 400 MHz): δ = 3.70 (s, 3H), 5.52 (d, 1H, *J* = 12.6 Hz), 5.91 (s, 1H), 7.04 (dd, 1H, *J* = 5.1 and 3.6 Hz), 7.33 (d, 1H, *J* = 3.6 Hz), 7.47 (d, 1H, *J* = 5.1 Hz), 7.52 (d, 1H, *J* = 12.6 Hz). ^13^C NMR (CDCl_3_, 100 MHz): δ = 51.4, 65.5, 101.6, 114.6, 127.3, 129.4, 129.5, 132.9, 157.3, 166.6 ppm. HRMS (ESI^+^): *m*/*z* [M + Na]^+^ calculated for C_10_H_9_NO_3_SNa 246.0201, found 246.0201. (*Z*-isomer, minor): (115.1 mg, 26%). Orange oil: ^1^H NMR (CDCl_3_, 400 MHz): δ = 3.70 (s, 3H), 5.11 (d, 1H, *J* = 7.2 Hz), 6.06 (s, 1H), 6.63 (d, 1H, *J* = 7.2 Hz), 7.06 (dd, 1H, *J* = 5.1 and 3.7 Hz), 7.38 (d, 1H, *J* = 3.7 Hz), 7.50 (d, 1H, *J* = 5.1 Hz). ^13^C NMR (CDCl_3_, 100 MHz): δ = 51.2, 67.0, 101.3, 114.9, 127.4, 129.7, 129.8, 133.2, 151.6, 164.4 ppm.

*Methyl 3-(benzo[b]thiophen-3-yl(cyano)methoxy)acrylate* (**7q**). (*E*-isomer, major): (347.8 mg, 64%). Yellow solid: ^1^H NMR (CDCl_3_, 500 MHz): δ = 3.71 (s, 3H), 5.59 (d, 1H, *J* = 12.5 Hz), 6.00 (s, 1H), 7.43–7.49 (m, 2H), 7.60 (d, 1H, *J* = 12.5 Hz), 7.80 (d, 1H, *J* = 7.2 Hz), 7.83 (s, 1H), 7.90 (d, 1H, *J* = 7.2 Hz), ^13^C NMR (CDCl_3_, 125 MHz): δ = 51.5, 65.4, 101.6, 114.6, 121.5, 123.1, 125.2, 125.5, 125.6, 129.4, 135.4, 140.7, 157.5, 166.6 ppm. HRMS (ESI^+^): *m*/*z* [M + Na]^+^ calculated for C_14_H_11_NO_5_SNa 296.0357, found 296.0349. (*Z*-isomer, minor): (152.5 mg, 28%). Yellow oil: ^1^H NMR (CDCl_3_, 500 MHz): δ = 3.69 (s, 3H), 5.07 (d, 1H, *J* = 7.1 Hz), 6.16 (s, 1H), 6.65 (d, 1H, *J* = 7.1 Hz), 7.42–7.48 (m, 2H), 7.86 (s, 1H), 7.88 (d, 1H, *J* = 7.0 Hz), 7.95 (d, 1H, *J* = 7.0 Hz), ^13^C NMR (CDCl_3_, 125 MHz): δ = 51.2, 67.2, 101.0, 115.0, 122.0, 123.0, 125.3, 125.6, 125.9, 129.2, 135.4, 140.7, 152.0, 164.4 ppm.

*Methyl 3-(1-cyanopropoxy)acrylate* (**3r**). (*E*-isomer, major): (207.5 mg, 62%). Transparent liquid: ^1^H NMR (CDCl_3_, 400 MHz): δ = 1.11 (t, 3H, *J* = 7.4 Hz), 1.97–2.04 (m, 2H), 3.70 (s, 3H), 4.54 (t, 1H, *J* = 6.5 Hz), 5.41 (d, 1H, *J* = 12.6 Hz), 7.56 (d, 1H, *J* = 12.6 Hz). ^13^C NMR (CDCl_3_, 100 MHz): δ = 8.8, 26.5, 51.4, 69.4, 100.3, 115.9, 158.7, 166.8 ppm. HRMS (ESI^+^): *m*/*z* [M + Na]^+^ calculated for C_8_H_11_NO_3_Na 192.0637, found 192.0633. (*Z*-isomer, minor): (99.0 mg, 29%). Transparent liquid: ^1^H NMR (CDCl_3_, 400 MHz): δ = 1.13 (t, 3H, *J* = 7.4 Hz), 1.99–2.07 (m, 2H), 3.68 (s, 3H), 4.60 (t, 1H, *J* = 6.5 Hz), 5.07 (d, 1H, *J* = 7.0 Hz), 6.58 (d, 1H, *J* = 7.0 Hz). ^13^C NMR (CDCl_3_, 100 MHz): δ = 8.8, 26.7, 51.1, 72.1, 100.1, 116.4, 153.8, 164.5 ppm.

*Methyl 3-(1-cyanobutoxy)acrylate* (**7s**). (*E*-isomer, major): (219.4 mg, 59%). Transparent liquid: ^1^H NMR (CDCl_3_, 400 MHz): δ = 0.95 (t, 3H, *J* = 7.2 Hz), 1.46–1.55 (m, 2H), 1.87–1.94 (m, 2H), 3.66 (s, 3H), 4.58 (t, 1H, *J* = 6.5 Hz), 5.39 (d, 1H, *J* = 12.6 Hz), 7.46 (d, 1H, *J* = 12.6 Hz). ^13^C NMR (CDCl_3_, 100 MHz): δ = 13.1, 17.6, 34.6, 51.2, 68.1, 100.2, 116.1, 158.7, 166.7 ppm. HRMS (ESI^+^): *m*/*z* [M + Na]^+^ calculated for C_9_H_13_NO_3_Na 206.0793, found 206.0792. (*Z*-isomer, minor): (113.6 mg, 31%). Transparent liquid: ^1^H NMR (CDCl_3_, 400 MHz): δ = 0.98 (t, 3H, *J* = 7.1 Hz), 1.54–1.63 (m, 2H), 1.93–2.03 (m, 2H), 3.69 (s, 3H), 4.64 (t, 1H, *J* = 6.5 Hz), 5.07 (d, 1H, *J* = 7.1 Hz), 6.57 (d, 1H, *J* = 7.1 Hz). ^13^C NMR (CDCl_3_, 100 MHz): δ = 13.2, 17.8, 35.0, 51.1, 70.9, 100.1, 116.5, 153.8, 164 ppm.

*Methyl 3-((1-cyanopentyl)oxy)acrylate* (**7t**). (*E*-isomer, major): (169.3 mg, 43%). Transparent oil: ^1^H NMR (CDCl_3_, 500 MHz): δ = 0.90 (t, 3H, *J* = 7.72 Hz), 1.31–1.39 (m, 2H), 1.44–1.50 (m, 2H), 1.92–1.96 (m, 2H), 3.68 (s, 3H), 4.58 (t, 1H, *J* = 6.50 Hz), 5.40 (d, 1H, *J* = 12.8 Hz), 7.47 (d, 1H, *J* = 12.8 Hz). ^13^C NMR (CDCl_3_, 125 MHz): δ = 13.5, 21.8, 26.3, 32.5, 51.3, 68.3, 100.2, 116.1, 158.7, 166.8 ppm. HRMS (ESI^+^): *m*/*z* [M + Na]^+^ calculated for C_10_H_15_NO_3_ 220.0950, found 220.0952. (*Z*-isomer, minor): (88.2 mg, 22%). Transparent oil: ^1^H NMR (CDCl_3_, 500 MHz): δ = 0.92 (t, 3H, *J* = 7.2 Hz), 1.34–1.41 (m, 2H), 1.48–1.54 (m, 2H), 1.94–2.02 (m, 2H), 3.67 (s, 3H), 4.63 (t, 1H, *J* = 6.5 Hz), 5.06 (d, 1H, *J* = 7.14 Hz), 6.57 (d, 1H, *J* = 7.1 Hz). ^13^C NMR (CDCl_3_, 125 MHz): δ = 13.6, 21.8, 26.3, 32.7, 51.0, 71.1, 99.9, 116.5, 153.8, 164.5 ppm.

*Methyl 3-((1-cyanoheptyl)oxy)acrylate* (**7u**). (*E*-isomer, major): (173.3 mg, 39%). Transparent oil: ^1^H NMR (CDCl_3_, 500 MHz): δ = 0.84 (t, 3H, *J* = 6.9 Hz), 1.25–1.33 (m, 6H), 1.43–1.49 (m, 2H), 1.90–1.95 (m, 2H), 3.66 (s, 3H), 4.58 (t, 1H, *J* = 6.5 Hz), 5.39 (d, 1H, *J* = 12.2 Hz), 7.47 (d, 1H, *J* = 12.2 Hz). ^13^C NMR (CDCl_3_, 125 MHz): δ = 13.7, 22.2, 24.1, 28.2, 31.2, 32.7, 51.2, 68.3, 100.1, 116.0, 158.6, 166.7 ppm. HRMS (ESI^+^): *m*/*z* [M + Na]^+^ calculated for C_12_H_19_NO_3_Na 248.1263, found 248.1260. (*Z*-isomer, minor): (73.0 mg, 16%). Transparent oil: ^1^H NMR (CDCl_3_, 500 MHz): δ = 0.87 (t, 3H, *J* = 6.9 Hz), 1.27–1.31 (m, 4H), 1.32.−1.38 (m, 2H), 1.50–1.56 (m, 2H), 1.95–2.03 (m, 2H), 3.68 (s, 3H), 4.64 (t, 1H, *J* = 6.8 Hz), 5.06 (d, 1H, *J* = 7.1 Hz), 6.58 (d, 1H, *J* = 7.1 Hz). ^13^C NMR (CDCl_3_, 125 MHz): δ = 13.9, 22.4, 24.3, 28.3, 31.3, 33.0, 51.1, 71.1, 100.1, 153.8, 164.5 ppm.

*Methyl 3-(1-cyano-2-methylpropoxy)acrylate* (**7v**). (*E*-isomer, major): (181.7 mg, 50%). Transparent oil: ^1^H NMR (CDCl_3_, 400 MHz): δ = 1.05 (d, 3H, *J* = 6.7 Hz), 1.08 (d, 3H, *J* = 6.7 Hz), 2.15–2.21 (m, 1H), 3.66 (s, 3H), 4.40 (d, 1H, *J* = 5.8 Hz), 5.46 (d, 1H, *J* = 12.7 Hz), 7.47 (d, 1H, *J* = 12.7 Hz). ^13^C NMR (CDCl_3_, 100 MHz): δ = 17.0, 17.4, 51.2, 73.7, 100.0, 115.2, 158.9, 166.8 ppm. HRMS (ESI^+^): *m*/*z* [M + Na]^+^ calculated for C_9_H_13_NO_3_Na 206.0793, found 206.0792. (*Z*-isomer, minor): (85.5 mg, 23%). Yellow oil: ^1^H NMR (CDCl_3_, 400 MHz): δ = 1.11 (d, 3H, *J* = 6.7 Hz), 1.14 (d, 3H, *J* = 6.7 Hz), 2.20–2.29 (m, 1H), 3.68 (s, 3H), 4.39 (d, 1H, *J* = 6.9 Hz), 5.05 (d, 1H, *J* = 7.0 Hz), 6.56 (d, 1H, *J* = 7.0 Hz). ^13^C NMR (CDCl_3_, 100 MHz): δ = 17.3, 17.6, 51.1, 76.7, 100.0, 115.7, 154.1, 164.6 ppm.

*Methyl 3-(1-cyano-2,2-dimethylpropoxy)acrylate* (**7w**). (*E*-isomer, major): (534 mg, 68%). Transparent oil: ^1^H NMR (CDCl_3_, 500 MHz): δ = 1.11 (s, 9H), 3.71 (s, 3H), 4.19 (s, 1H), 5.44 (d, 1H, *J* = 12.7 Hz), 7.52 (d, 1H, *J* = 12.7 Hz). ^13^C NMR (CDCl_3_, 125 MHz): δ = 25.0 (3C), 35.4, 51.4, 77.5, 100.0, 115.2, 159.2, 166.9 ppm. HRMS (ESI^+^): *m*/*z* [M + Na]^+^ calculated for C_10_H_15_NO_3_Na 220.0950, found 220.0951. (*Z*-isomer, minor): (213.5 mg, 27%). White solid: ^1^H NMR (CDCl_3_, 500 MHz): δ = 1.11 (s, 9H), 3.66 (s, 3H), 4.21 (s, 1H), 5.01 (d, 1H, *J* = 6.8 Hz), 6.56 (d, 1H, *J* = 6.8 Hz). ^13^C NMR (CDCl_3_, 125 MHz): δ = 25.0 (3C), 35.6, 50.9, 80.3, 99.5, 115.7, 154.8, 164.5 ppm.

## 4. Conclusions

We have shown that the organocatalytic multicomponent cyanovinylation of aldehydes is a powerful synthetic tool for the synthesis of conjugated cyanomethyl vinyl ethers **3**. The power of this synthetic tool was demonstrated in the synthesis of an array of 3-(cyanomethoxy)acrylates **7** endowed with a diverse substitution pattern at the position C-3. The reaction was performed in a three-component format using as inputs an aldehyde, acetone cyanohydrin (**5**), methyl propiolate (**4**) and a catalytic amount of *N*-methylmorpholine. The 3CR was performed at room temperature in *n*-hexanes to deliver (*E*/*Z*)-3-substituted-3-(cyanomethoxy)acrylates **7** in excellent yields and with a preponderance of the *E*-isomer. The reaction is highly tolerant to the structure and composition of the aldehyde (aliphatic, aromatic, heteroaromatic), and it is instrumentally simple (one batch, open atmospheres), economical (2.5 mol% catalyst, stoichiometric reagents), environmentally friendly (no toxic waste), and sustainable (easy scalability).

## Figures and Tables

**Table 1:**
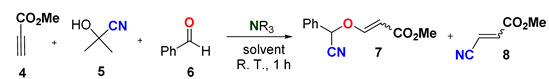
Optimization of the 3CR manifold ^a^.

Entry	Catalyst	Solvent	7 (%) ^b^	E/Z ^b^	8 (%)
1	DABCO (10 mol%)	CH_2_Cl_2_	78	65/13	14
2	DABCO (10 mol%)	*n-*Hexane	83	68/15	1
3	DABCO (10 mol%)	CHCl_3_	74	64/10	5
4	DABCO (10 mol%)	Benzene	84	74/10	3
5	DABCO (10 mol%)	THF	59	57/2	9
6	DABCO (10 mol%)	MeCN	68	64/4	10
7	Et_3_N (20 mol%)	*n-*Hexane	15	12/3	-
8	NMM (20 mol%)	*n-*Hexane	99	74/25	1
9	DIPEA (20 mol%)	*n-*Hexane	-	-	-
10	DCMA (20 mol%)	*n-*Hexane	-	-	-
11	Et_3_N (20 mol%)	CH_2_Cl_2_	75	65/10	16
12	DIPEA (20 mol%)	CH_2_Cl_2_	-		-
13	NMM (20 mol%)	CH_2_Cl_2_	89	71/18	8
14	NMM (20 mol%)	THF	55	45/10	1
15	NMM (20 mol%)	MeCN		64/8	4
16	NMM (20 mol%)	Benzene	-	-	-
17	NMM (20 mol%)	H_2_O	38	25/13	4
18	NMM (20 mol%)	Methanol	54	48/26	5
19	NMM (10 mol%)	*n-*Hexane	96	72/25	1
20	NMM (5 mol%)	*n-*Hexane	98	72/26	1
21	NMM (2.5 mol%)	*n-*Hexane	94	70/27	1
22	NMM (2.5 mol%)	*n-*Hexane ^c^	97	70/27	-
23	NMM (2.5 mol%)	*n-*Hexane ^d^	38	26/12	-
24	-	*n-*Hexane	n.o.r.	-	-

^a^ Experimental conditions: benzaldehyde (**6a**) (2 mmol), acetone cyanohydrin (**5**) (2 mmol), methyl propiolate (**4**) (2 mmol), *N*-methylmorpholine (0.05 mmol), *n*-hexanes (6 mL), R. T., 1 h. ^b^ Yields and *E*/*Z* ratios were calculated by ^1^H NMR of crude reaction mixtures. ^c^ 0 °C. ^d^ −78 °C. DABCO = 1,4-diazabicyclo[2.2.2]octane. NMM = *N*-methylmorpholine. DIPEA = **-diisopropylethylamine. DCMA = *N,N*-dicyclohexylmethylamine. THF = tetrahydrofuran. n.o.r. = no observed reaction.

## Supplementary Materials

^1^H and ^13^C NMR spectra for compounds **7** are available online.

## Sample Availability

Samples of the compounds **7a–w** are available from the authors.
