# Supplementary material for: Cyanovinylation of Aldehydes: Organocatalytic Multicomponent Synthesis of Conjugated Cyanomethyl Vinyl Ethers
Source: Molecules. 2021 Jul 6;26(14):4120. doi: 10.3390/molecules26144120 (PMC8304223; doi:10.3390/molecules26144120)
Supplement: Supplementary file 1 [file molecules-26-04120-s001.zip › molecules-1287838-supplementary.pdf]

Supplementary material

Cyanovinylation of aldehydes. Organocatalytic multicomponent synthesis of conjugated cyanomethyl vinyl ethers.

Samuel Delgado-Hernández, [1,2] Fernando García-Tellado [1,*] and David Tejedor [1,*]

[1] *Instituto de Productos Naturales y Agrobiología, Consejo Superior de Investigaciones Científicas, Astrofísico Francisco Sánchez 3, 38206 La Laguna, Tenerife, Spain.*
[2] *Doctoral and Postgraduate School, Universidad de La Laguna, Apartado Postal 456, 38200 La Laguna, Tenerife, Spain.*
* Correspondence: dtejedor@ipna.csic.es; fgarcia@ipna.csic.es

Table of contents **Pages**

1. ^{1}H NMR and ^{13}C NMR spectra of 3-(cyanomethoxy)acrylates **7** S2-46

1. NMR spectra.

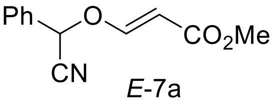

E-7a

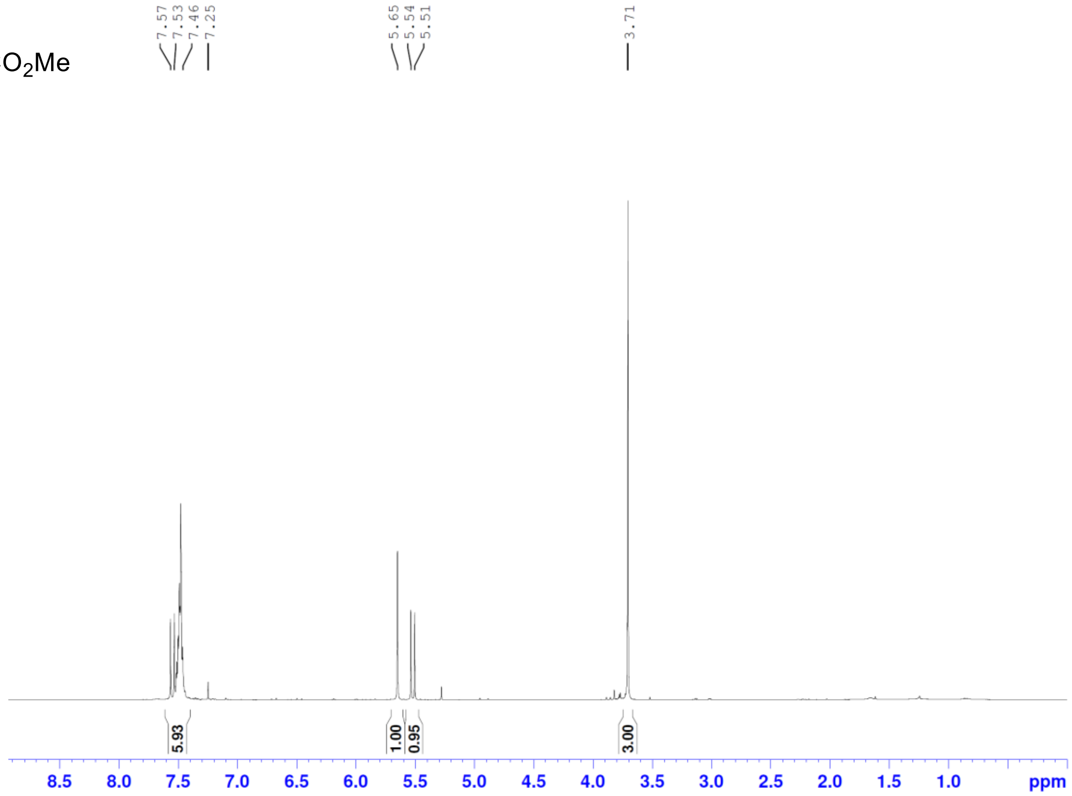

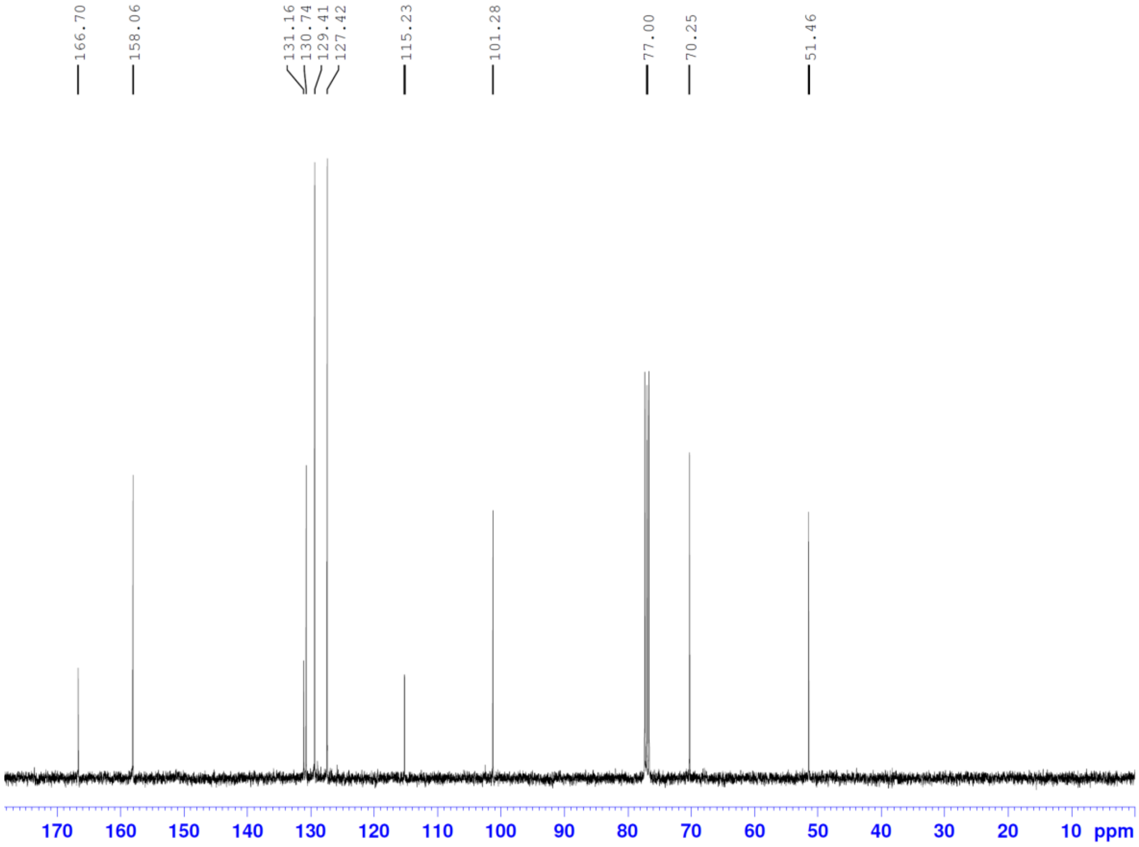

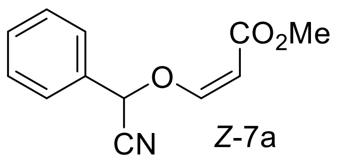

Z-7a

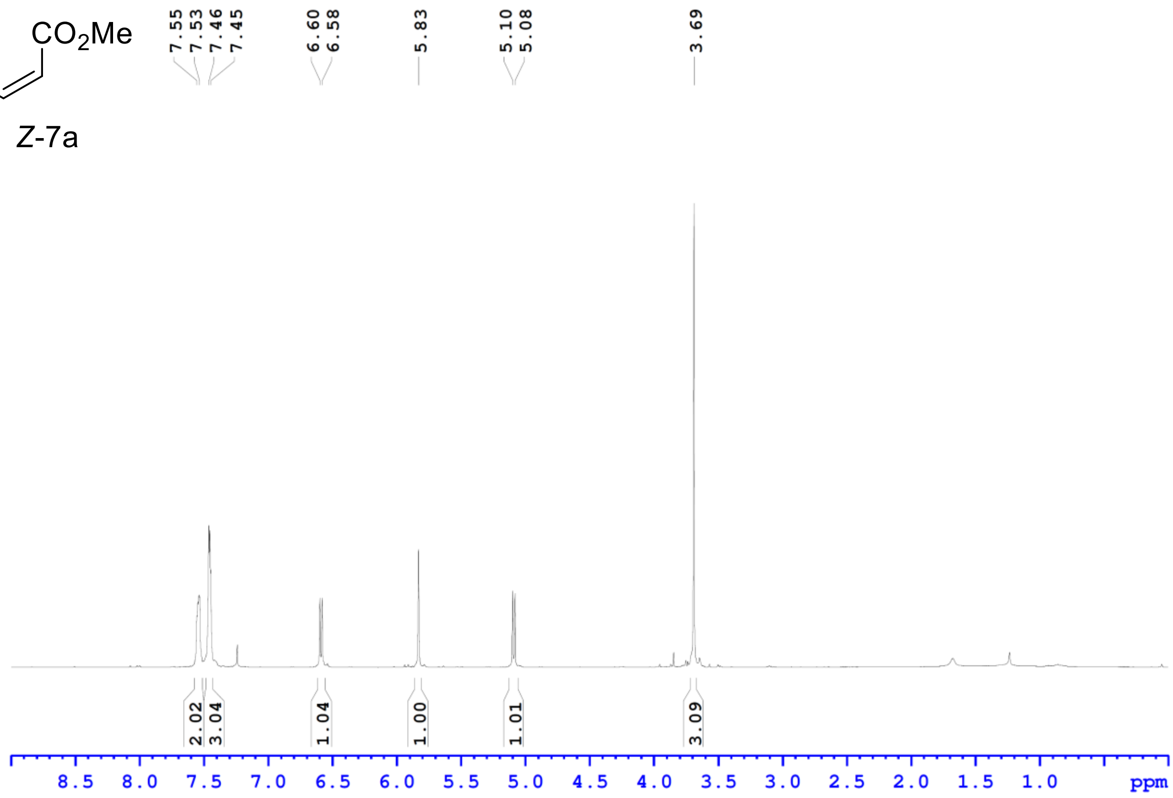

7.55
7.53
7.46
7.45
6.60
6.58
5.83
5.10
5.08
3.69
2.02
3.04
1.04
1.00
1.01
3.09
8.5 8.0 7.5 7.0 6.5 6.0 5.5 5.0 4.5 4.0 3.5 3.0 2.5 2.0 1.5 1.0 ppm

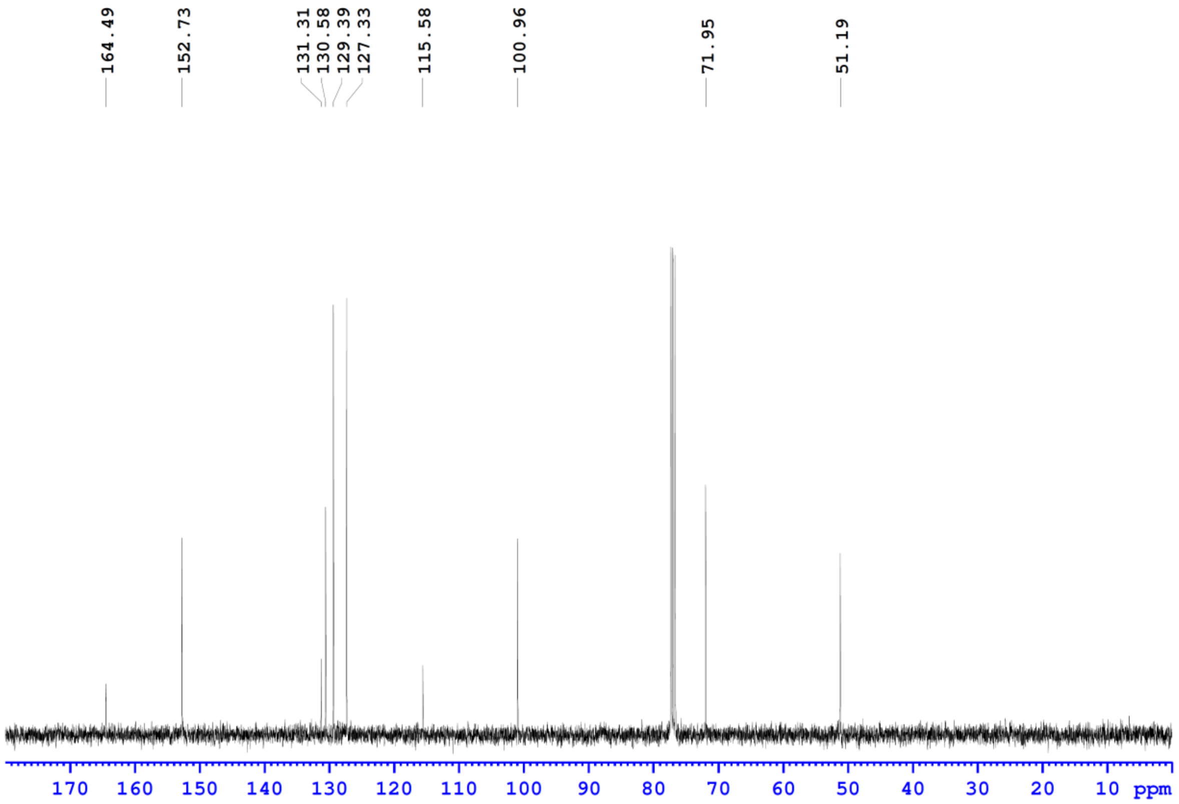

164.49
152.73
131.31
130.58
129.39
127.33
115.58
100.96
71.95
51.19
170 160 150 140 130 120 110 100 90 80 70 60 50 40 30 20 10 ppm

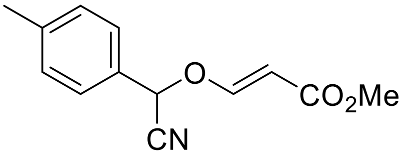
O
CO_2Me
CN

E-7b

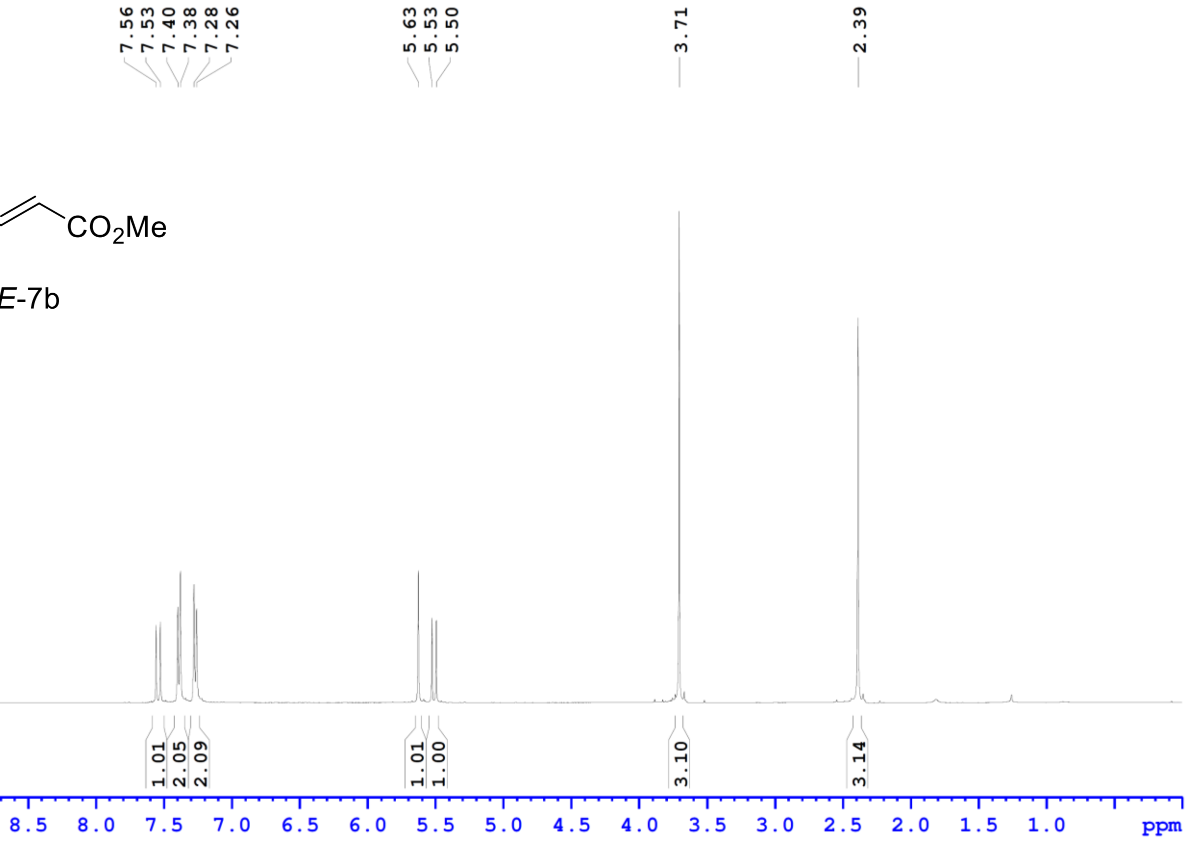
7.56
7.53
7.40
7.38
7.28
7.26
5.63
5.53
5.50
3.71
2.39
1.01
2.05
2.09
1.01
1.00
3.10
3.14
8.5 8.0 7.5 7.0 6.5 6.0 5.5 5.0 4.5 4.0 3.5 3.0 2.5 2.0 1.5 1.0 ppm

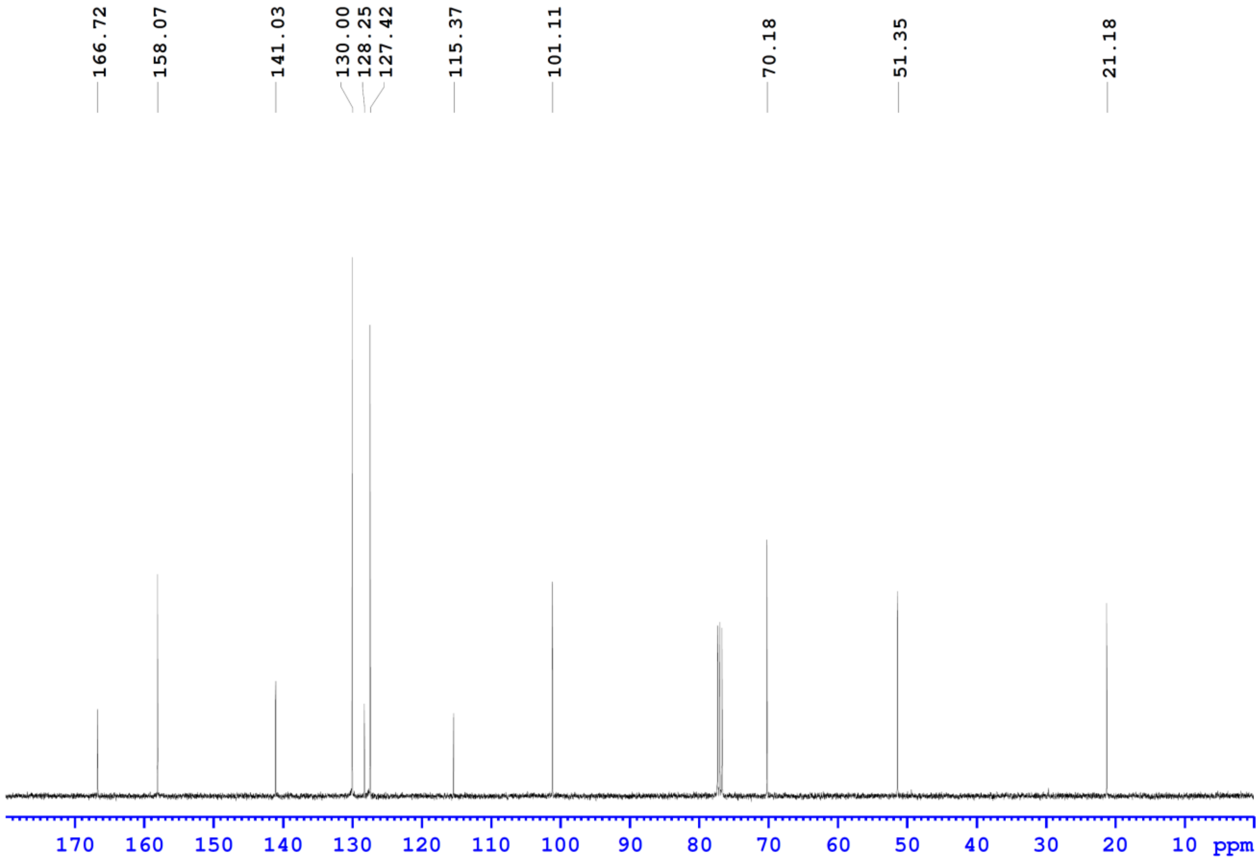
166.72
158.07
141.03
130.00
128.25
127.42
115.37
101.11
70.18
51.35
21.18
170 160 150 140 130 120 110 100 90 80 70 60 50 40 30 20 10 ppm

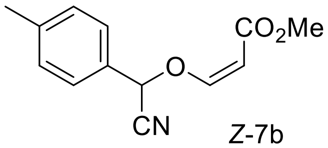
CO2Me
O
CN

Z-7b

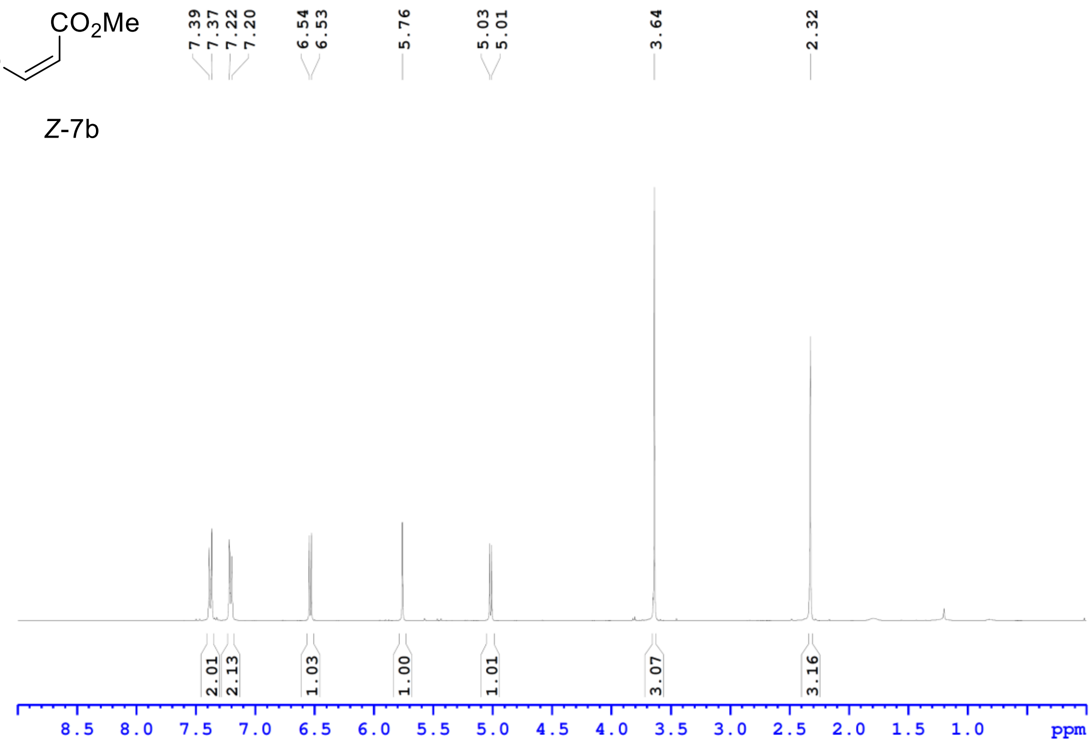
7.39
7.37
7.22
7.20
6.54
6.53
5.76
5.03
5.01
3.64
2.32
2.01
2.13
1.03
1.00
1.01
3.07
3.16
8.5 8.0 7.5 7.0 6.5 6.0 5.5 5.0 4.5 4.0 3.5 3.0 2.5 2.0 1.5 1.0 ppm

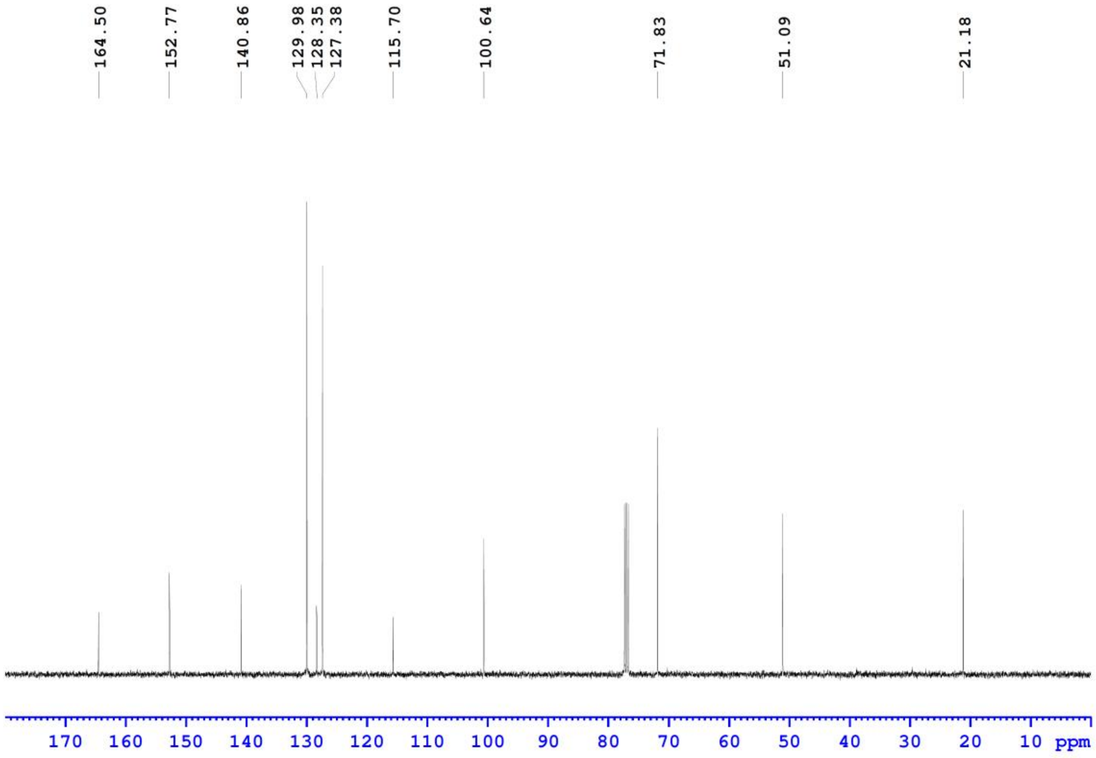
164.50
152.77
140.86
129.98
128.35
127.38
115.70
100.64
71.83
51.09
21.18
170 160 150 140 130 120 110 100 90 80 70 60 50 40 30 20 10 ppm

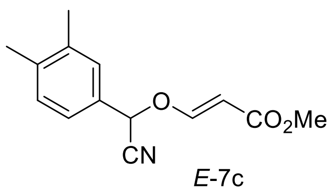

E-7c

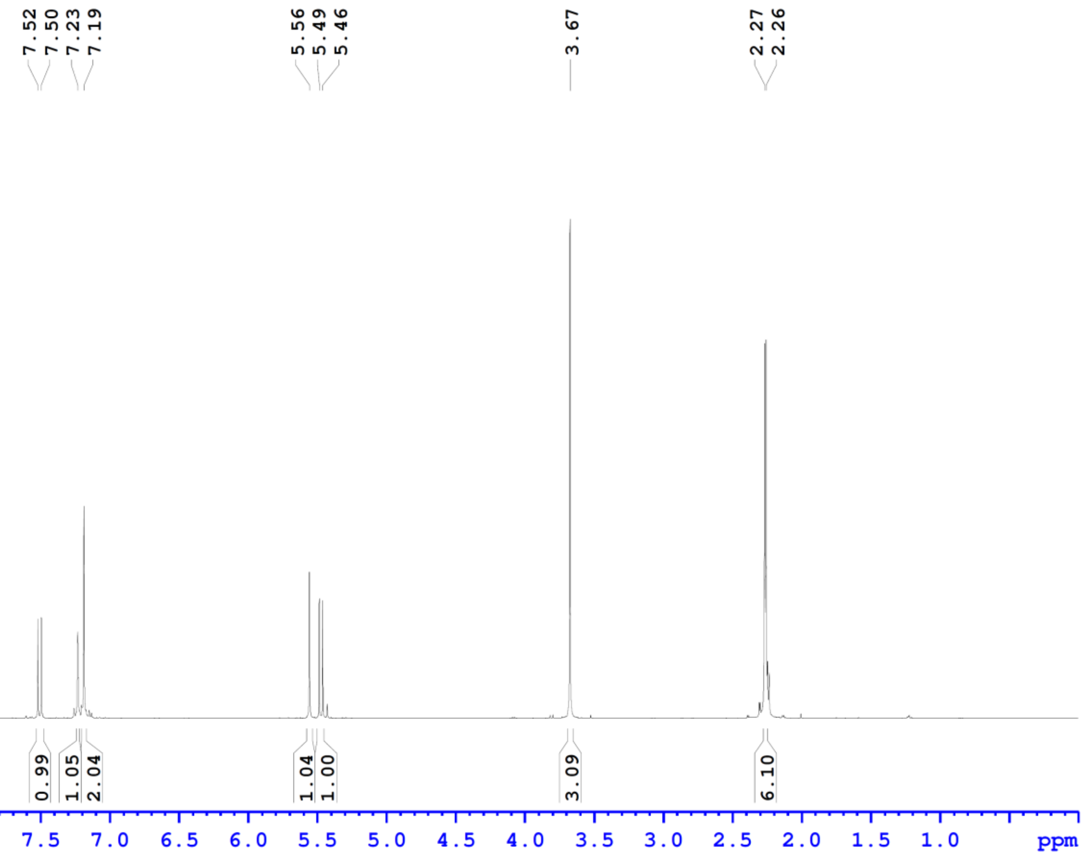

7.52
7.50
7.23
7.19
5.56
5.49
5.46
3.67
2.27
2.26
0.99
1.05
2.04
1.04
1.00
3.09
6.10
8.5 8.0 7.5 7.0 6.5 6.0 5.5 5.0 4.5 4.0 3.5 3.0 2.5 2.0 1.5 1.0 ppm

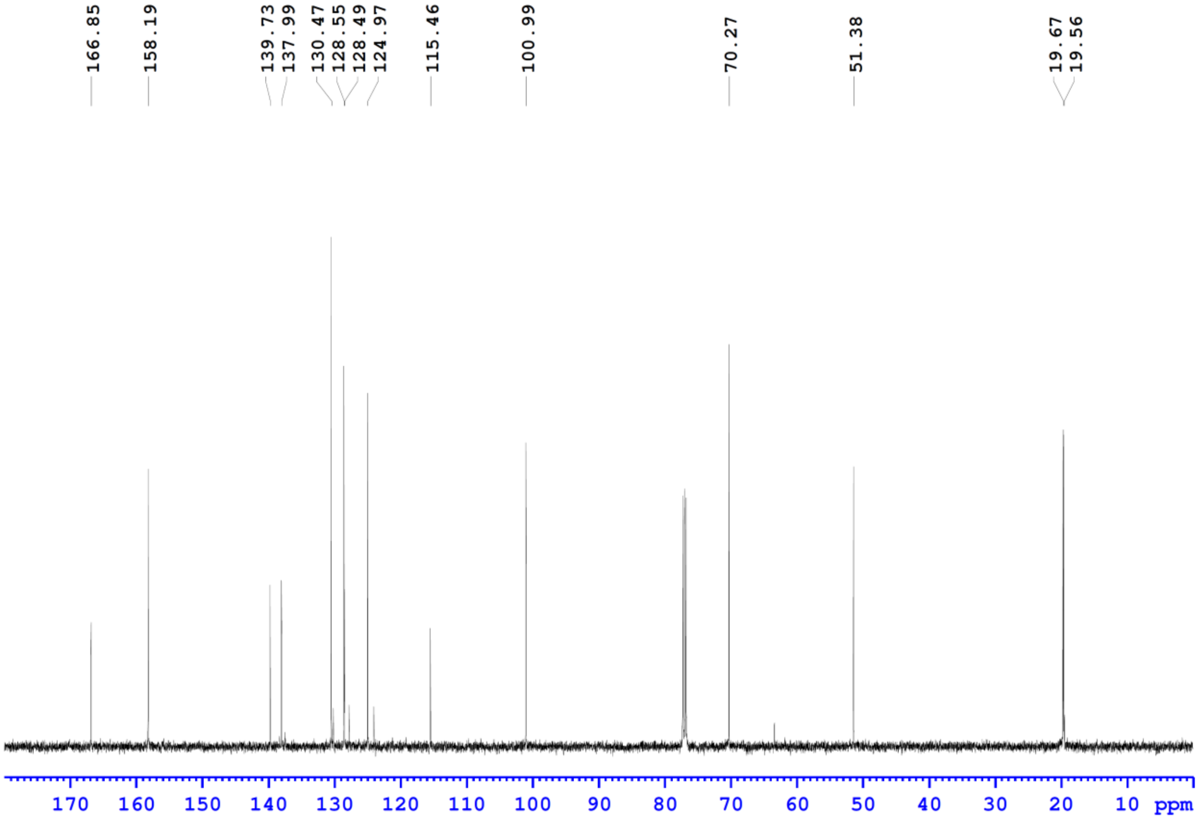

166.85
158.19
139.73
137.99
130.47
128.55
128.49
124.97
115.46
100.99
70.27
51.38
19.67
19.56
170 160 150 140 130 120 110 100 90 80 70 60 50 40 30 20 10 ppm

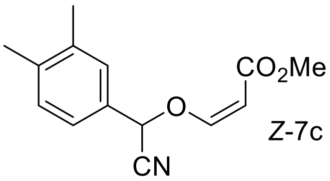
CO2Me
O
CN

Z-7c

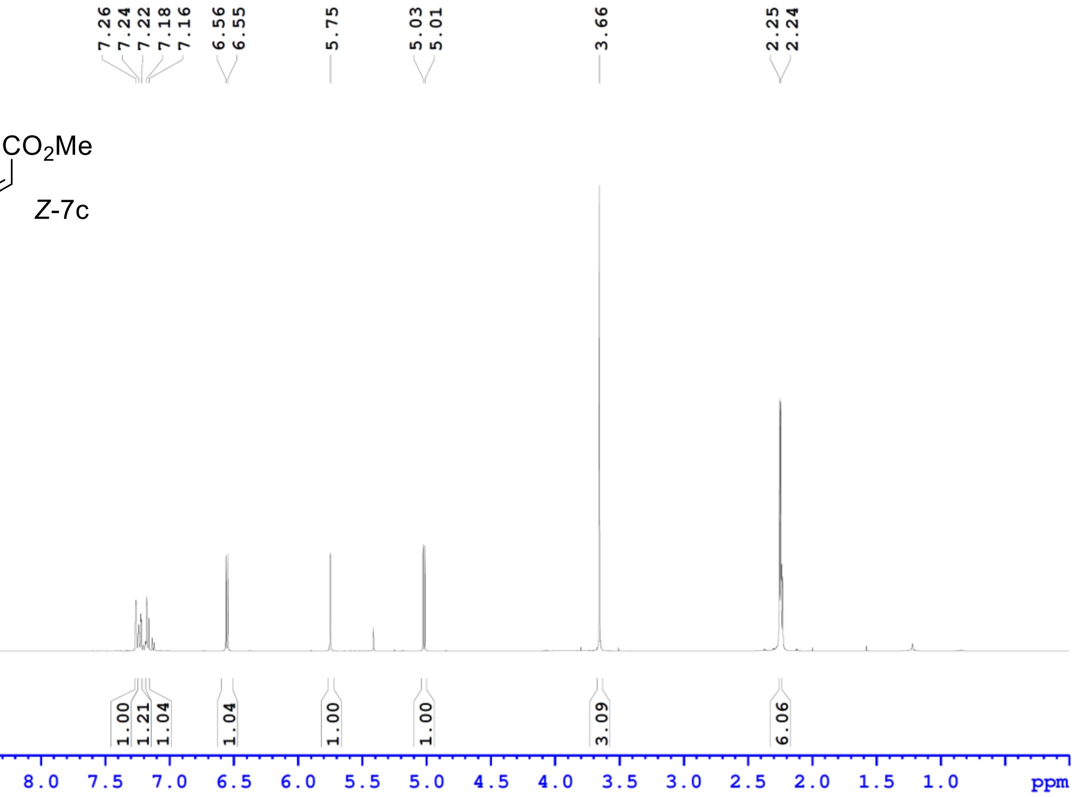
7.26
7.24
7.22
7.18
7.16
6.56
6.55
5.75
5.03
5.01
3.66
2.25
2.24
1.00
1.21
1.04
1.04
1.00
1.00
3.09
6.06
8.5 8.0 7.5 7.0 6.5 6.0 5.5 5.0 4.5 4.0 3.5 3.0 2.5 2.0 1.5 1.0 ppm

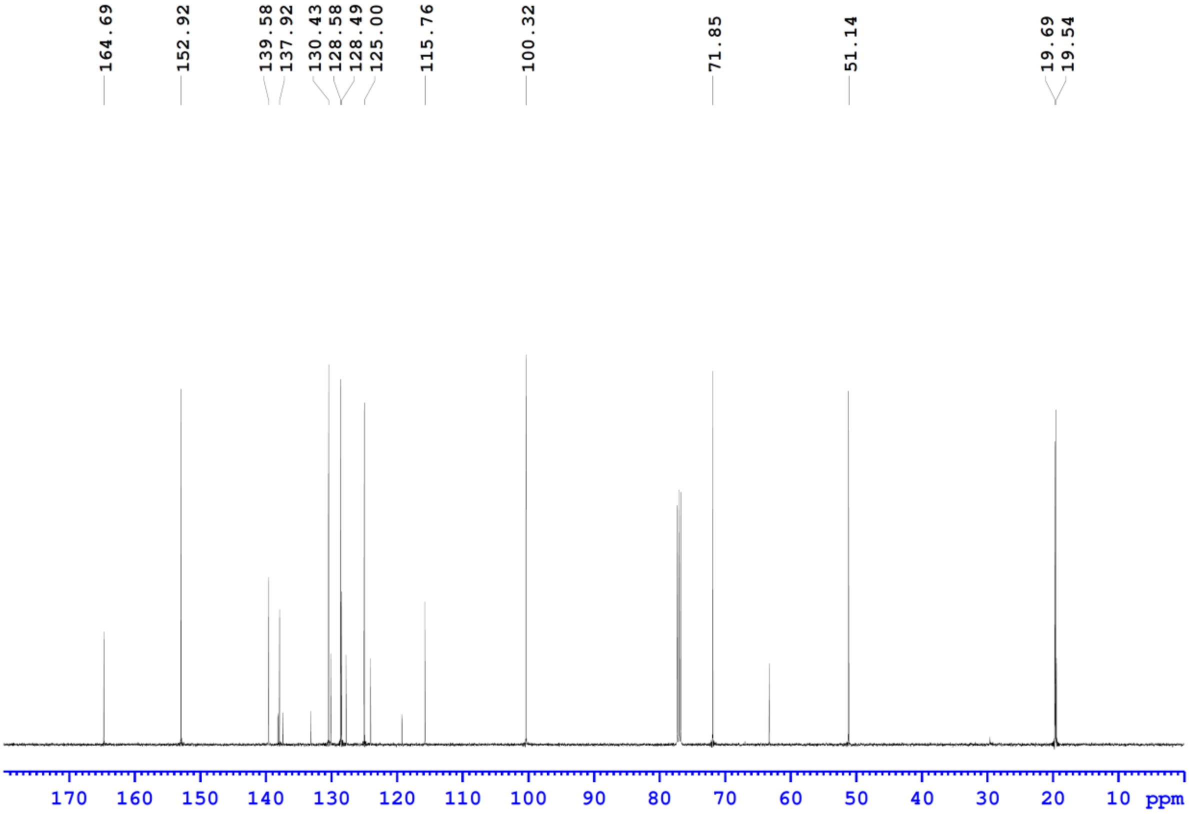
164.69
152.92
139.58
137.92
130.43
128.58
128.49
125.00
115.76
100.32
71.85
51.14
19.69
19.54
170 160 150 140 130 120 110 100 90 80 70 60 50 40 30 20 10 ppm

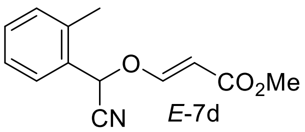
CN
CO2Me

E-7d

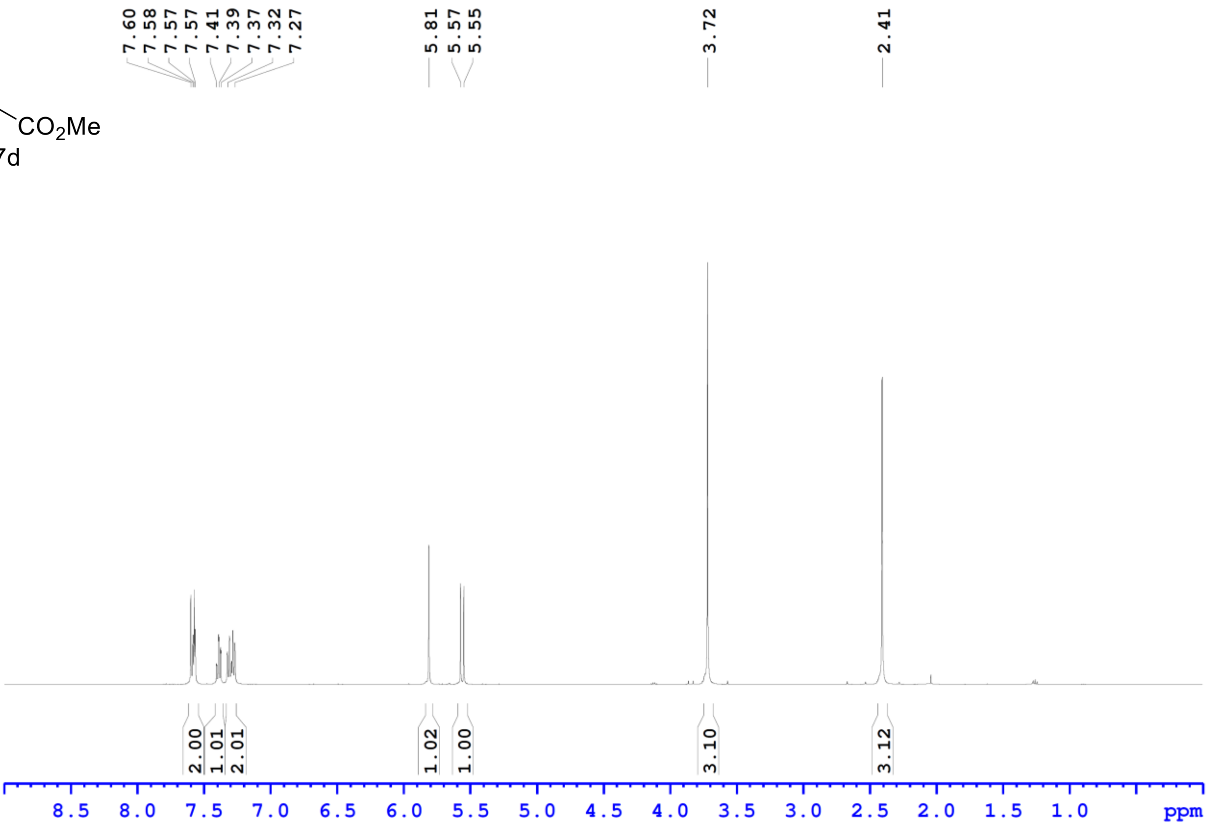
7.60
7.58
7.57
7.57
7.41
7.39
7.37
7.32
7.27
5.81
5.57
5.55
3.72
2.41
2.00
1.01
2.01
1.02
1.00
3.10
3.12
8.5 8.0 7.5 7.0 6.5 6.0 5.5 5.0 4.5 4.0 3.5 3.0 2.5 2.0 1.5 1.0 ppm

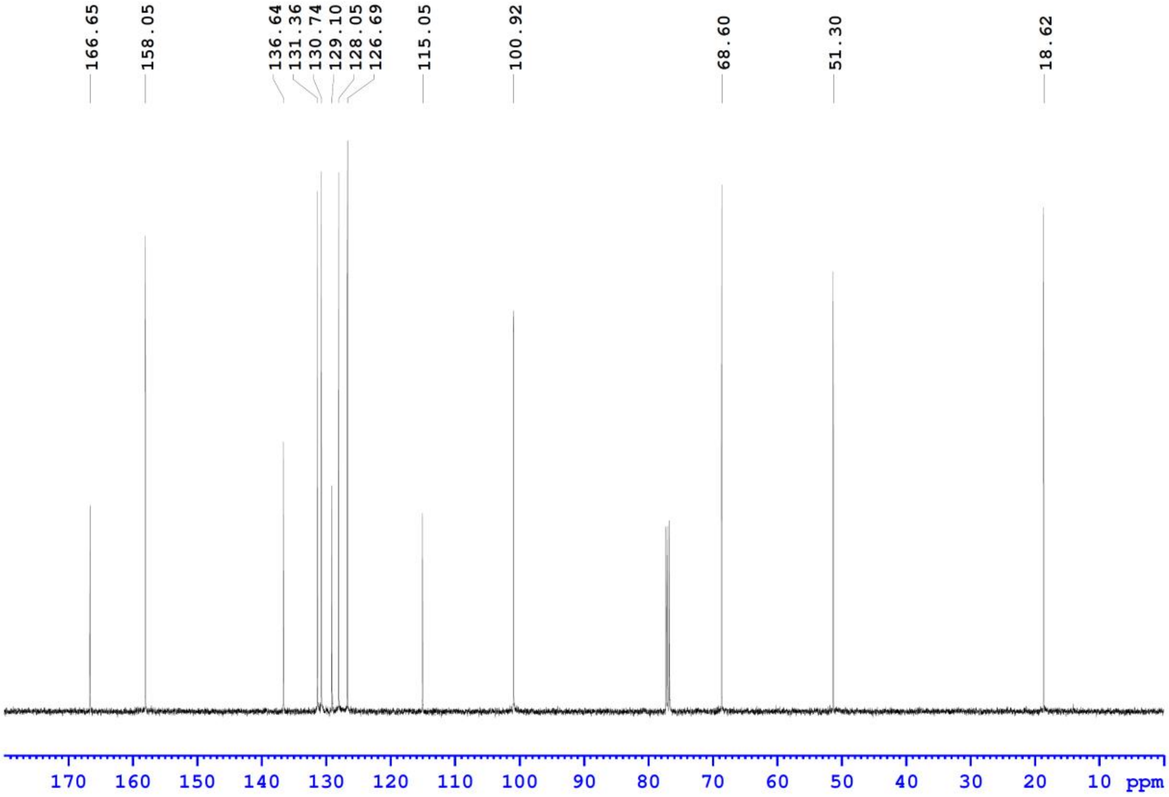
166.65
158.05
136.64
131.36
130.74
129.10
128.05
126.69
115.05
100.92
68.60
51.30
18.62
170 160 150 140 130 120 110 100 90 80 70 60 50 40 30 20 10 ppm

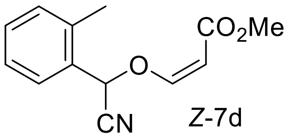
CO2Me
O
CN

Z-7d

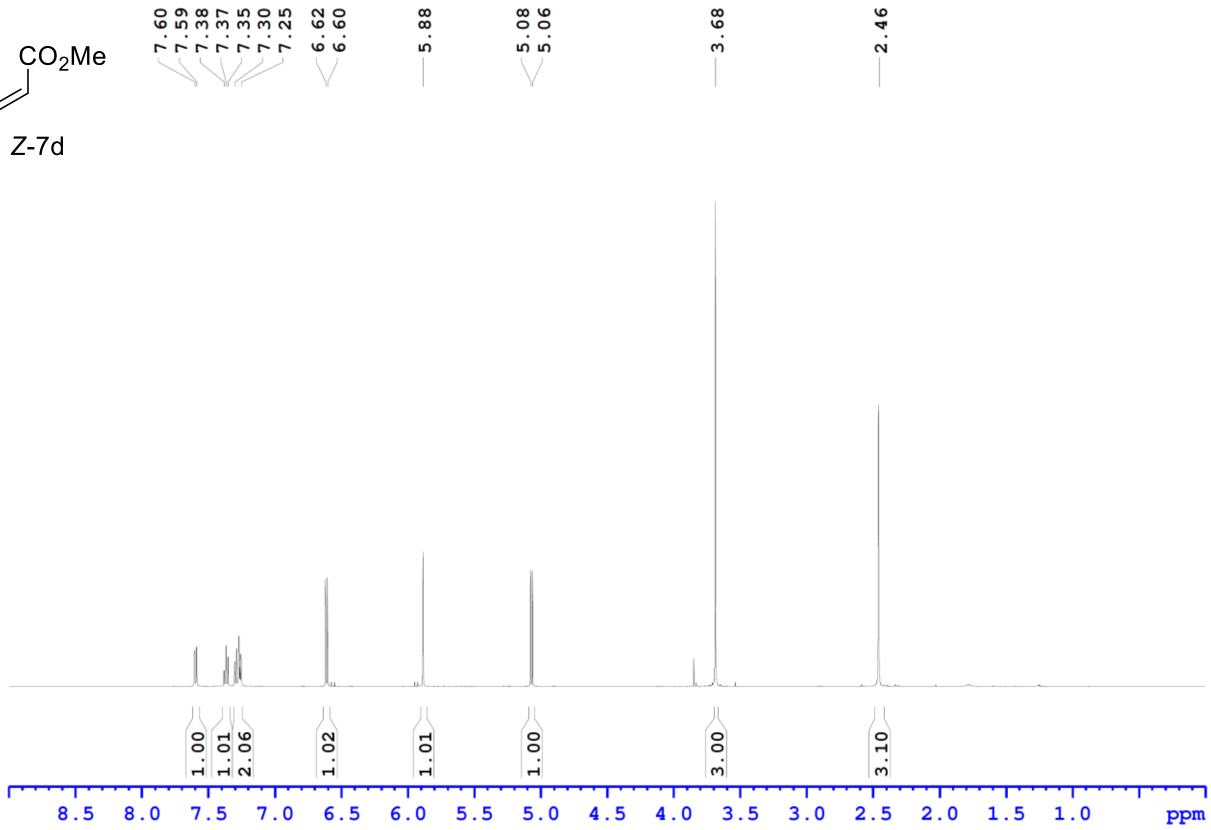
7.60
7.59
7.38
7.37
7.35
7.30
7.25
6.62
6.60
5.88
5.08
5.06
3.68
2.46
1.00
1.01
2.06
1.02
1.01
1.00
3.00
3.10
8.5 8.0 7.5 7.0 6.5 6.0 5.5 5.0 4.5 4.0 3.5 3.0 2.5 2.0 1.5 1.0 ppm

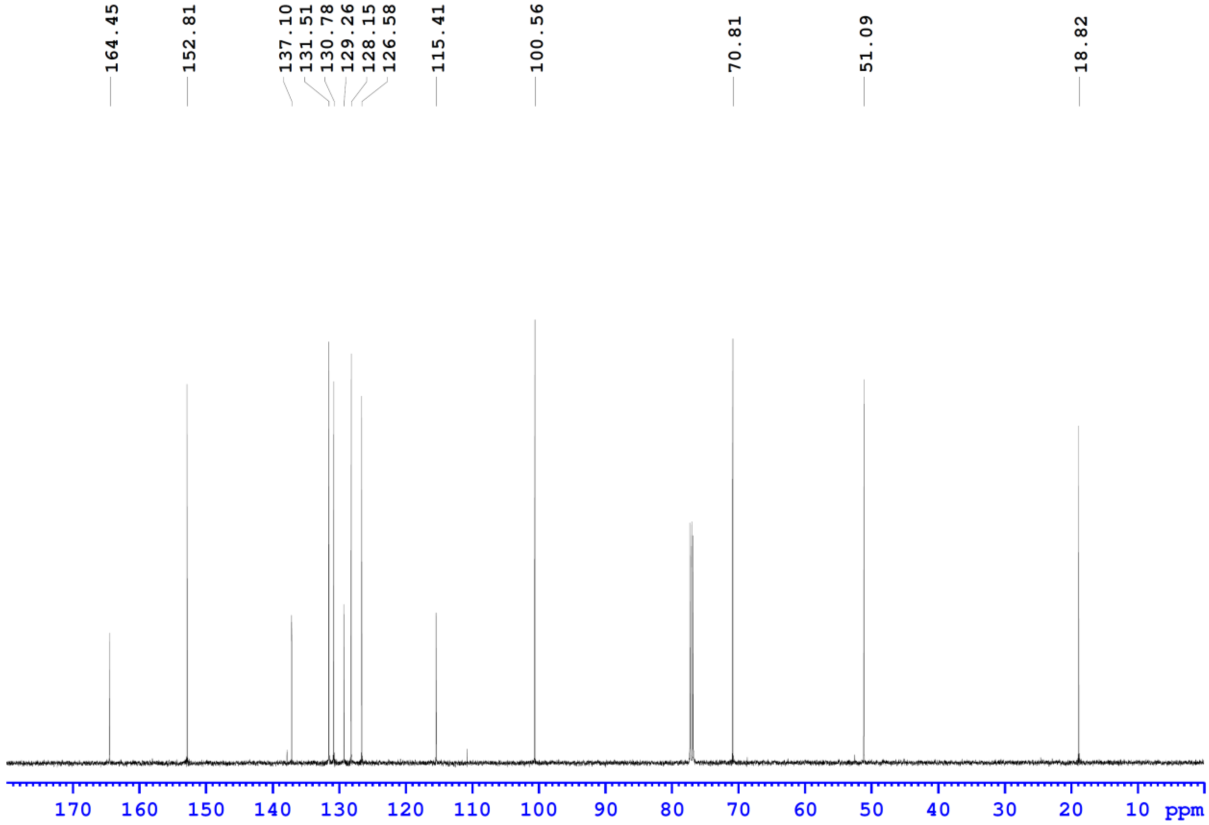
164.45
152.81
137.10
131.51
130.78
129.26
128.15
126.58
115.41
100.56
70.81
51.09
18.82
170 160 150 140 130 120 110 100 90 80 70 60 50 40 30 20 10 ppm

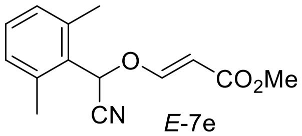

E-7e

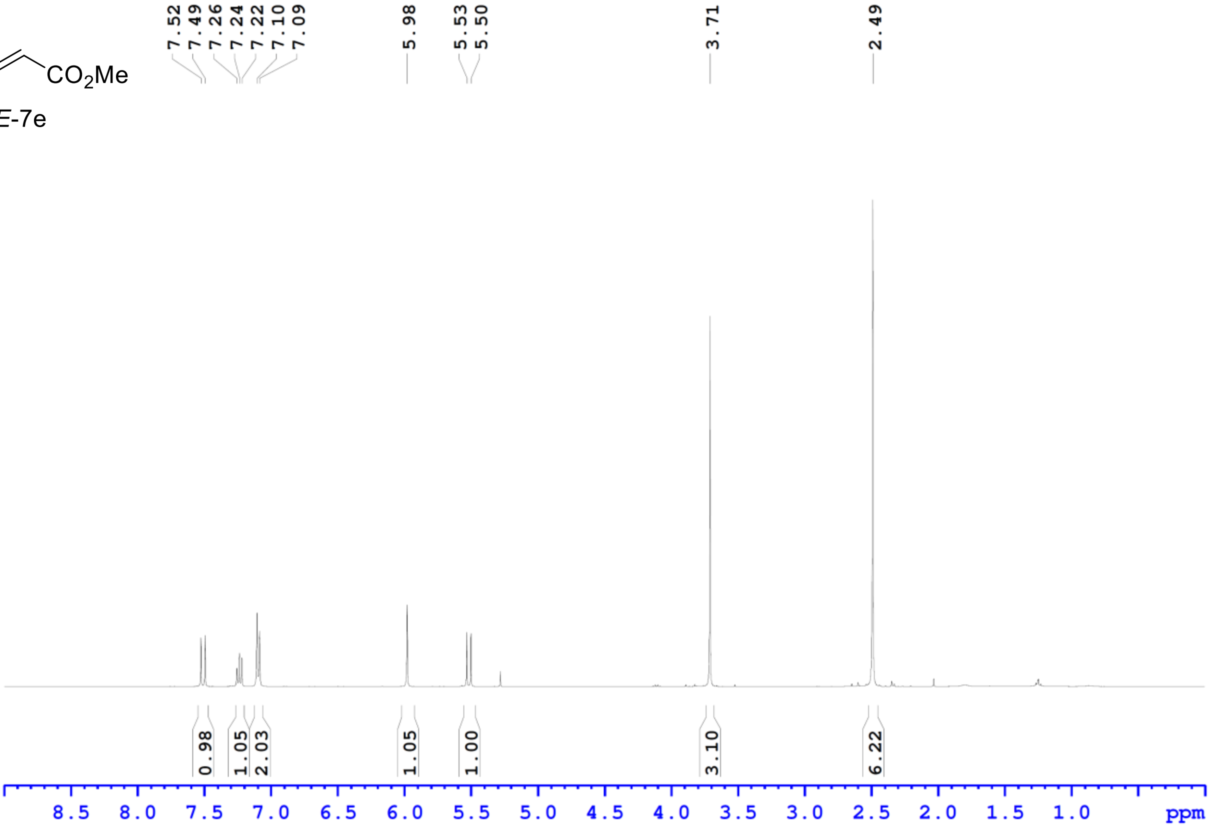

7.52
7.49
7.26
7.24
7.22
7.10
7.09
5.98
5.53
5.50
3.71
2.49
0.98
1.05
2.03
1.05
1.00
3.10
6.22
8.5 8.0 7.5 7.0 6.5 6.0 5.5 5.0 4.5 4.0 3.5 3.0 2.5 2.0 1.5 1.0 ppm

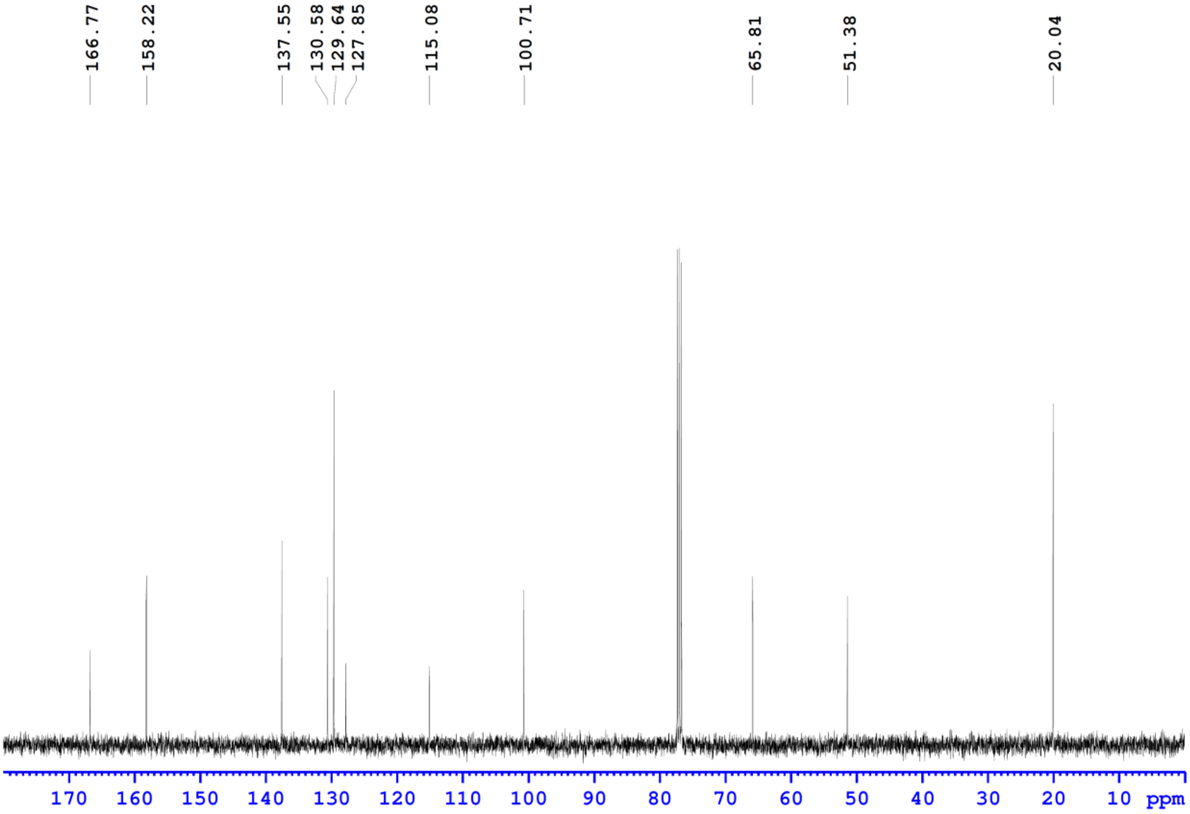

166.77
158.22
137.55
130.58
129.64
127.85
115.08
100.71
65.81
51.38
20.04
170 160 150 140 130 120 110 100 90 80 70 60 50 40 30 20 10 ppm

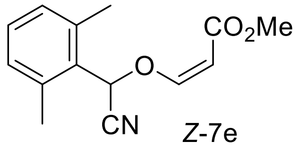
CO2Me
O
CN

Z-7e

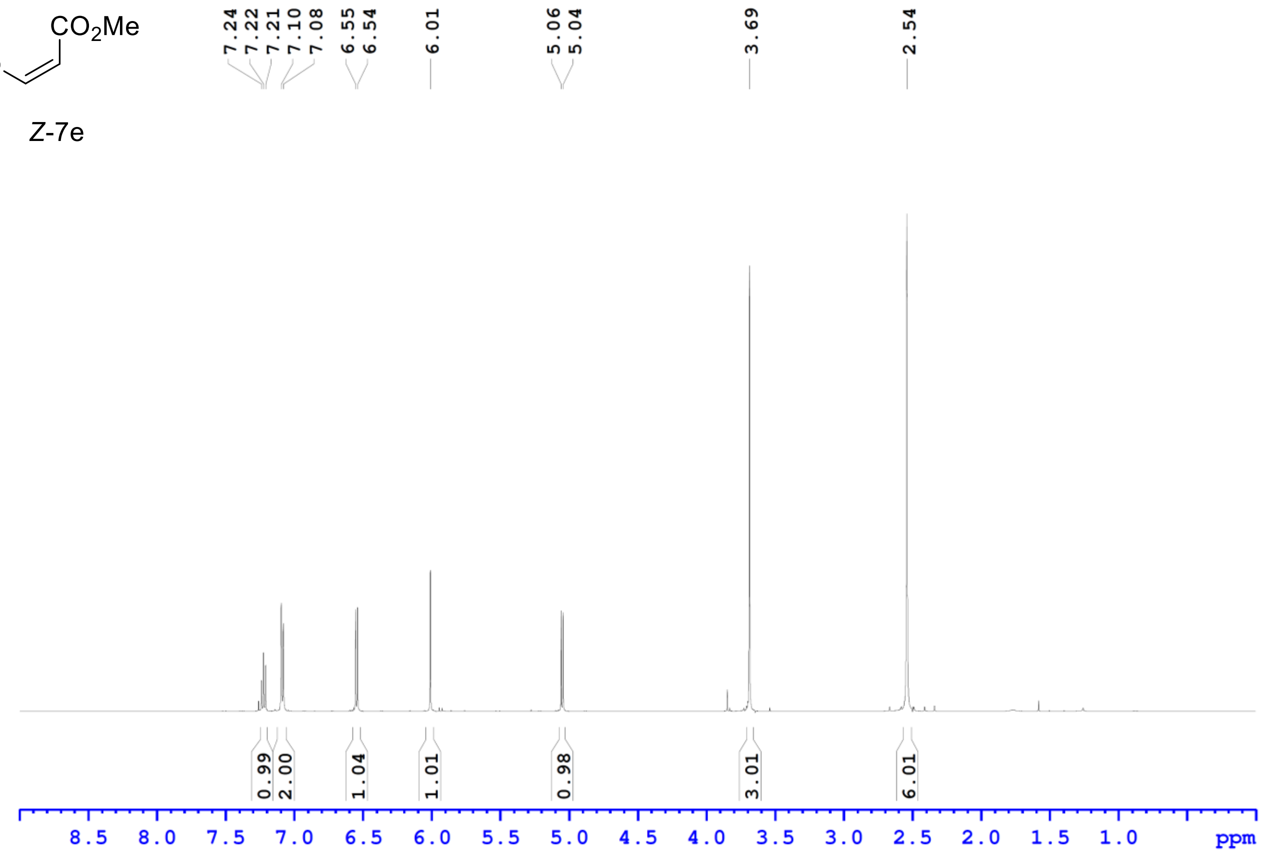
7.24
7.22
7.21
7.10
7.08
6.55
6.54
6.01
5.06
5.04
3.69
2.54
0.99
2.00
1.04
1.01
0.98
3.01
6.01
8.5 8.0 7.5 7.0 6.5 6.0 5.5 5.0 4.5 4.0 3.5 3.0 2.5 2.0 1.5 1.0 ppm

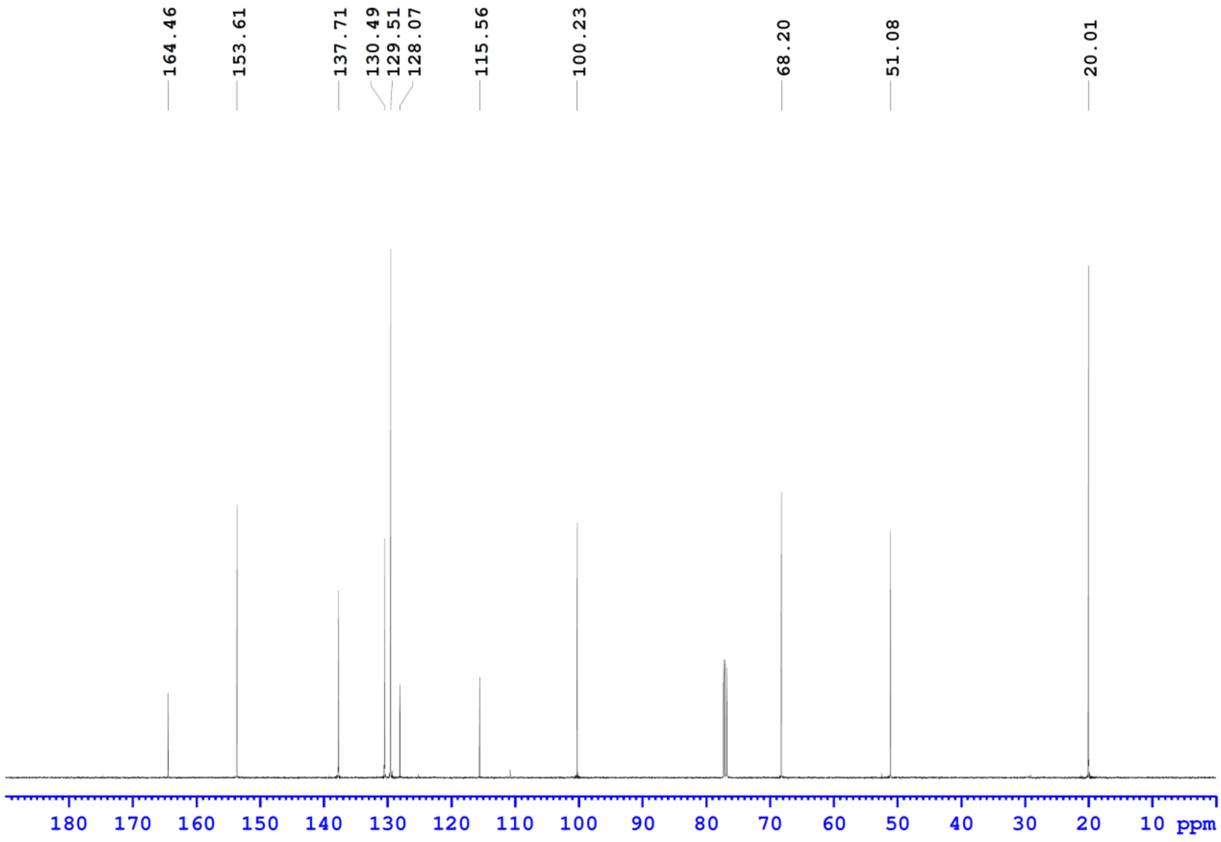
164.46
153.61
137.71
130.49
129.51
128.07
115.56
100.23
68.20
51.08
20.01
180 170 160 150 140 130 120 110 100 90 80 70 60 50 40 30 20 10 ppm

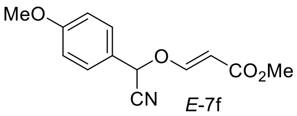

E-7f

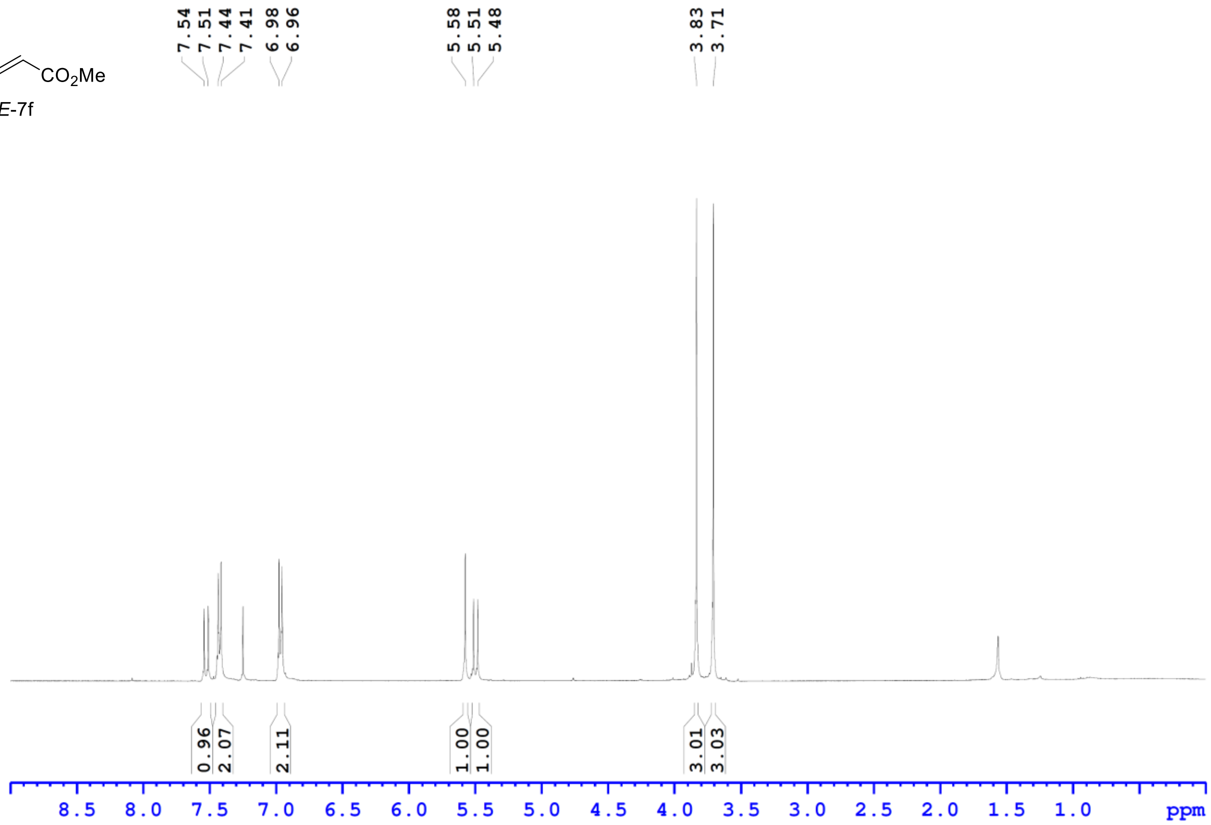
7.54
7.51
7.44
7.41
6.98
6.96
5.58
5.51
5.48
3.83
3.71
0.96
2.07
2.11
1.00
1.00
3.01
3.03
8.5 8.0 7.5 7.0 6.5 6.0 5.5 5.0 4.5 4.0 3.5 3.0 2.5 2.0 1.5 1.0 ppm

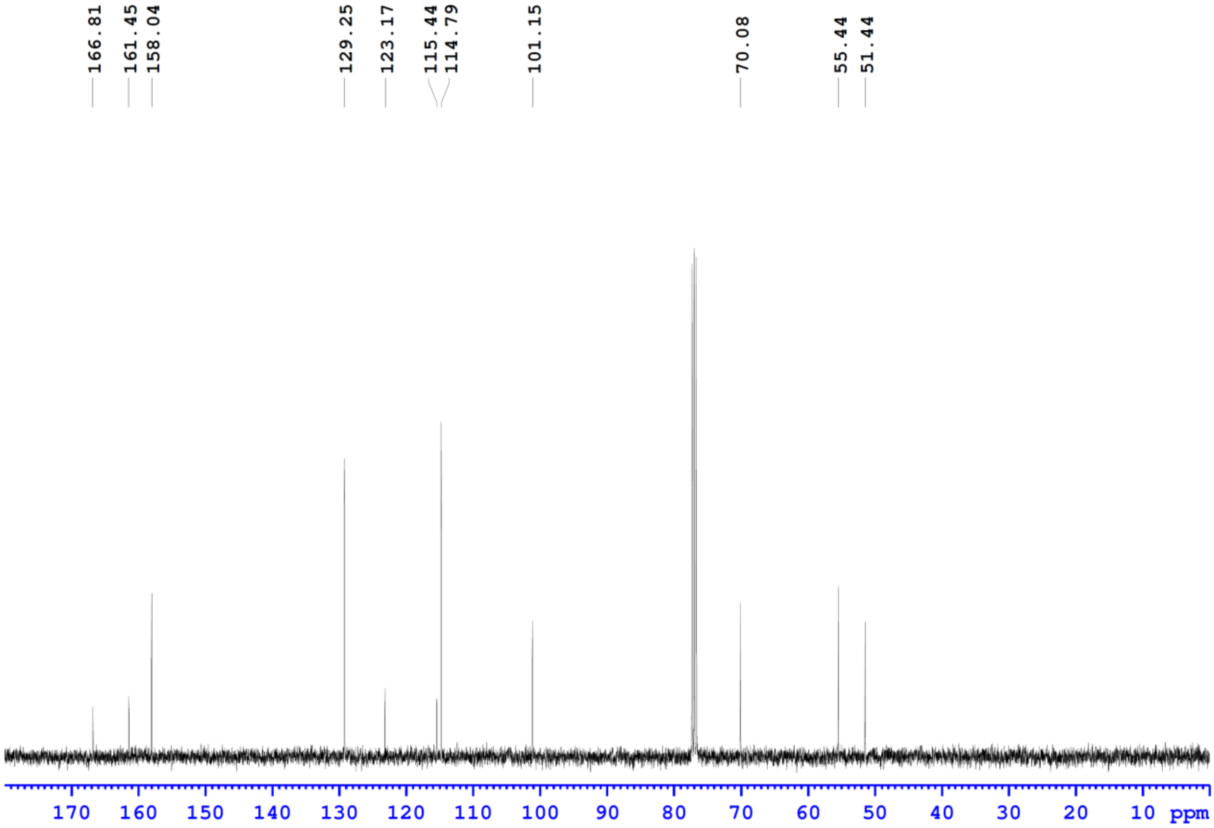
166.81
161.45
158.04
129.25
123.17
115.44
114.79
101.15
70.08
55.44
51.44
170 160 150 140 130 120 110 100 90 80 70 60 50 40 30 20 10 ppm

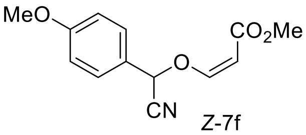
MeO
CO2Me
O
CN

Z-7f

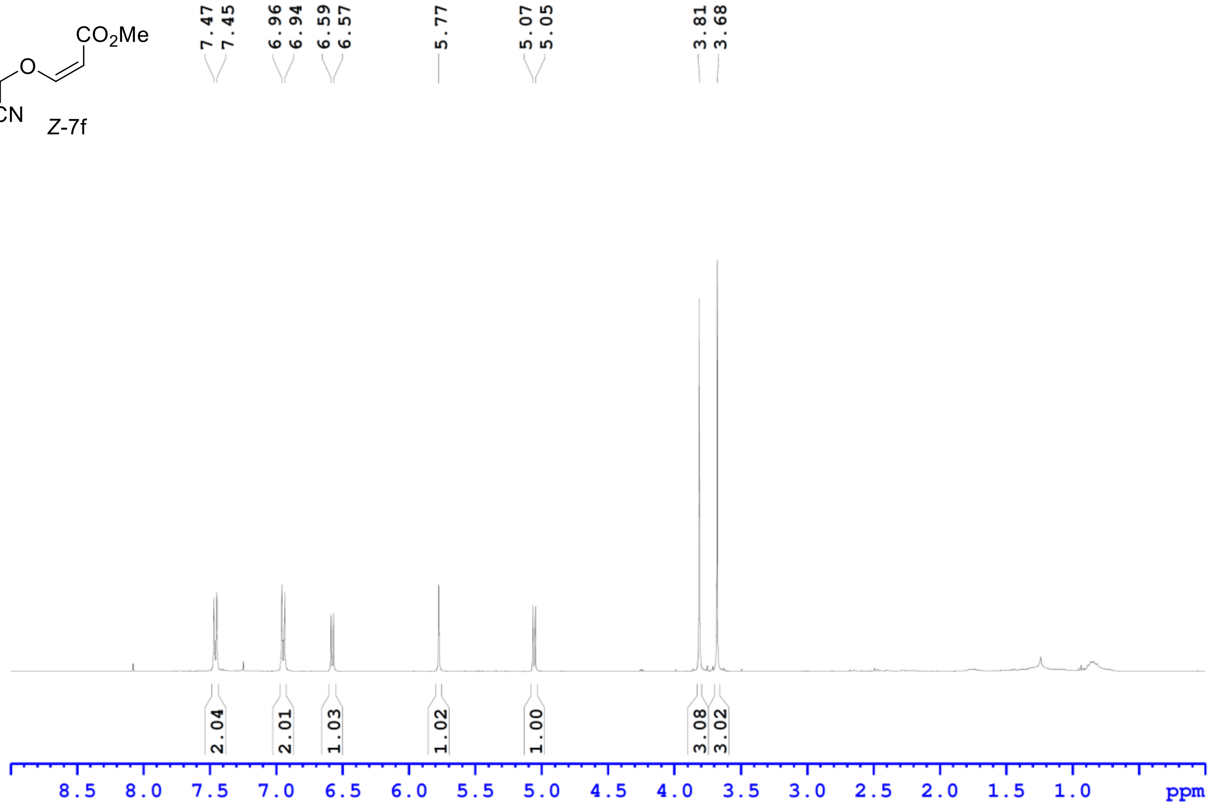
7.47
7.45
6.96
6.94
6.59
6.57
5.77
5.07
5.05
3.81
3.68
2.04
2.01
1.03
1.02
1.00
3.08
3.02
ppm

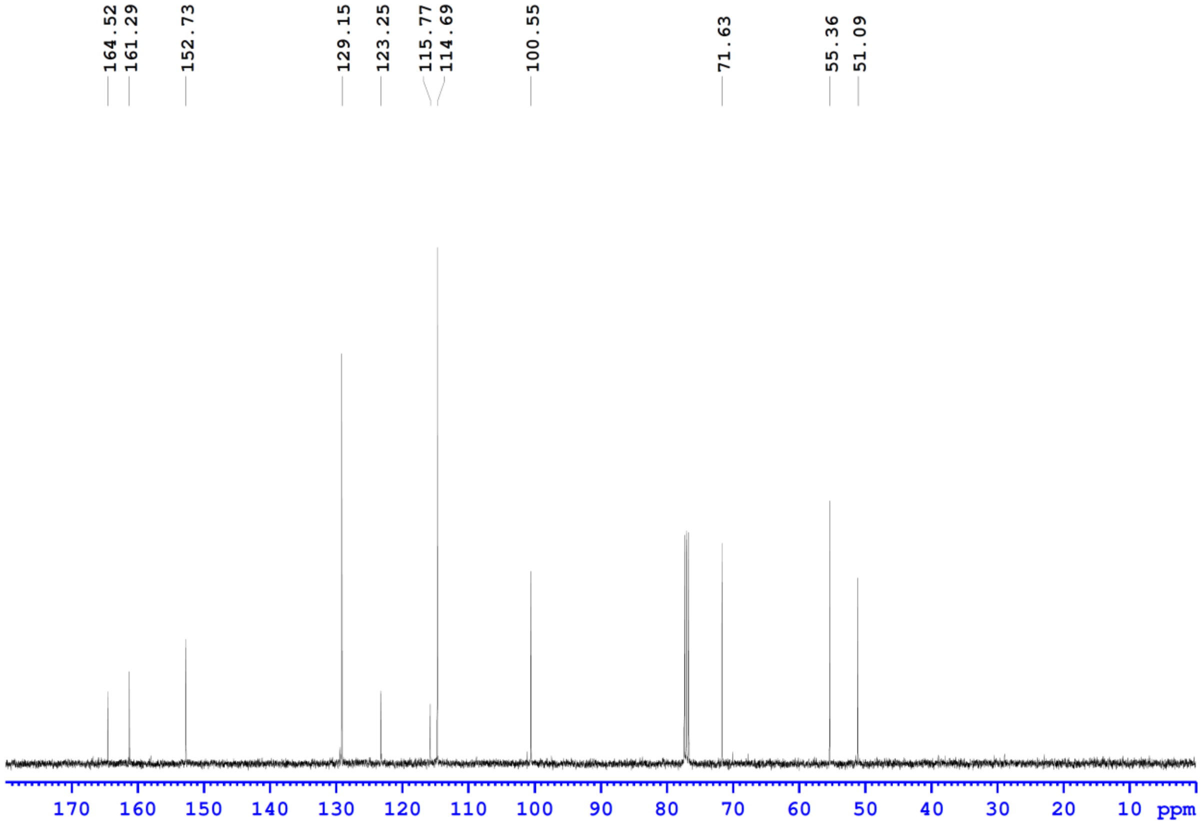
164.52
161.29
152.73
129.15
123.25
115.77
114.69
100.55
71.63
55.36
51.09
ppm

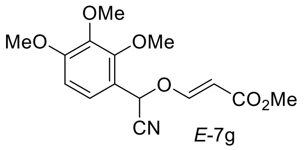
OMe
MeO
OMe
O
CO2Me
CN

E-7g

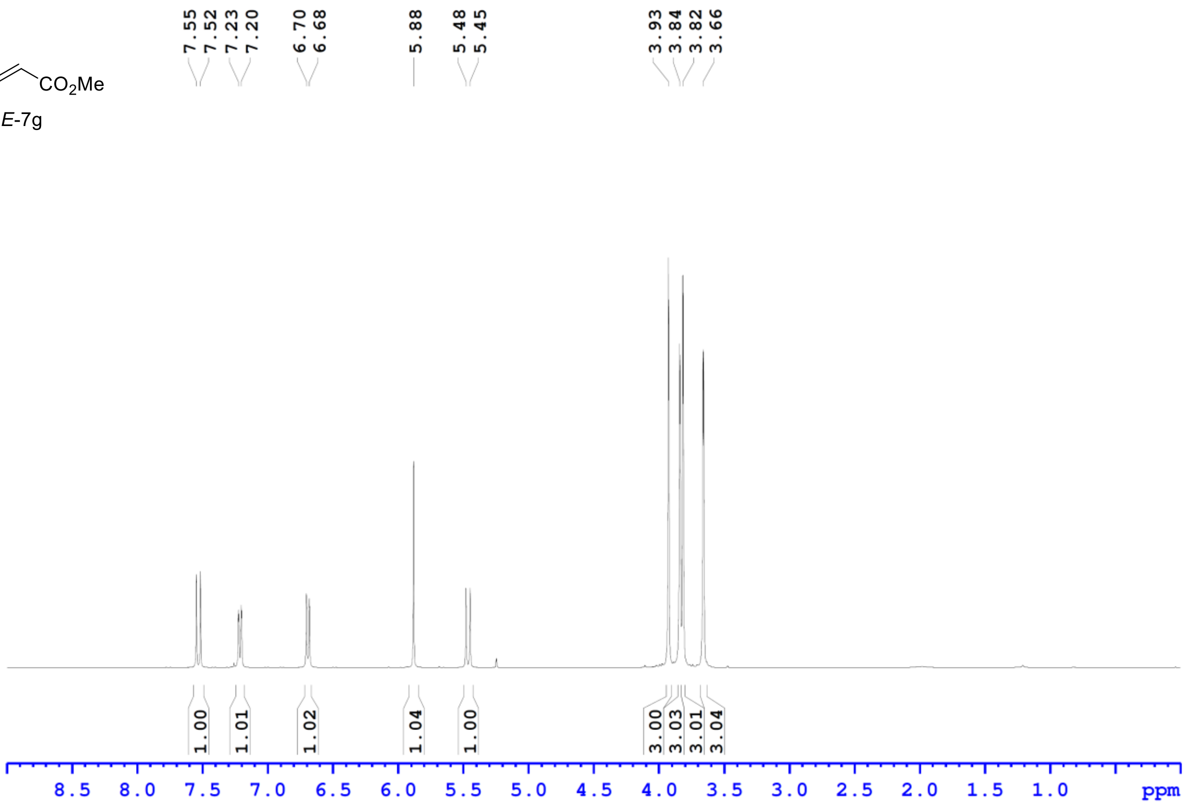
7.55
7.52
7.23
7.20
6.70
6.68
5.88
5.48
5.45
3.93
3.84
3.82
3.66
1.00
1.01
1.02
1.04
1.00
3.00
3.03
3.01
3.04
8.5
8.0
7.5
7.0
6.5
6.0
5.5
5.0
4.5
4.0
3.5
3.0
2.5
2.0
1.5
1.0
ppm

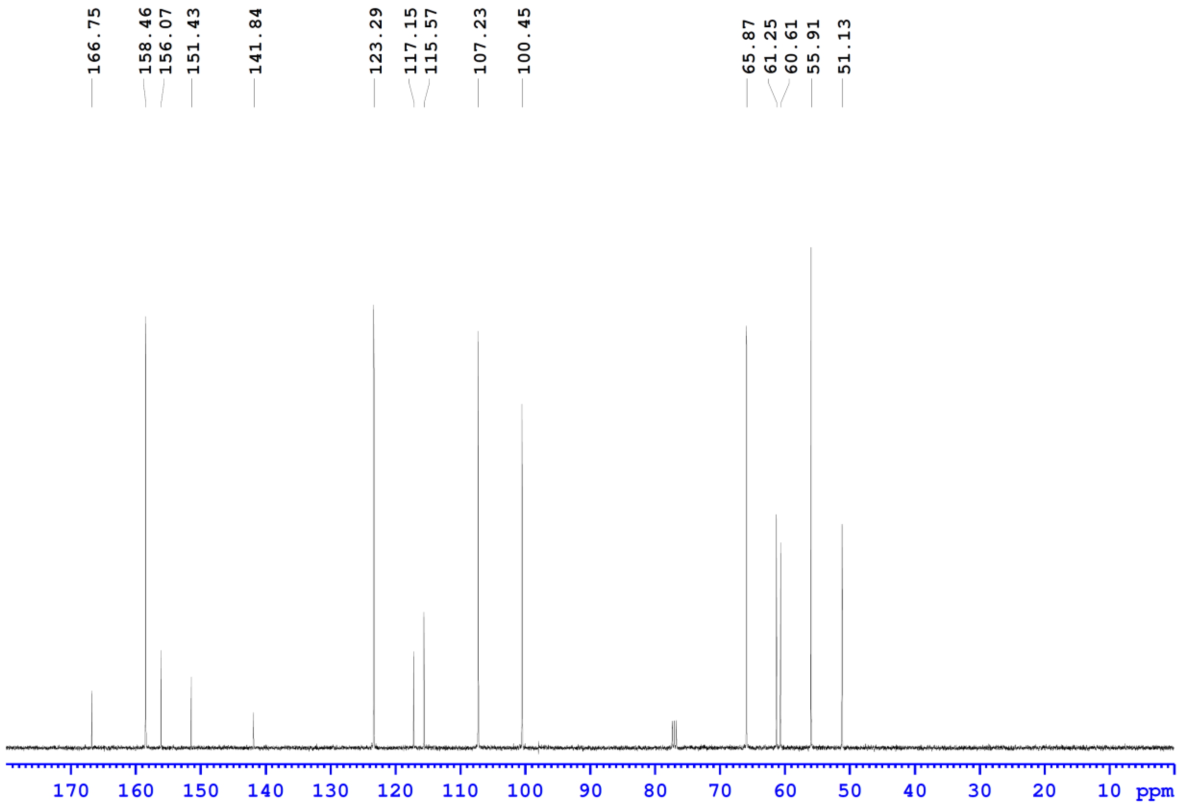
166.75
158.46
156.07
151.43
141.84
123.29
117.15
115.57
107.23
100.45
65.87
61.25
60.61
55.91
51.13
170
160
150
140
130
120
110
100
90
80
70
60
50
40
30
20
10
ppm

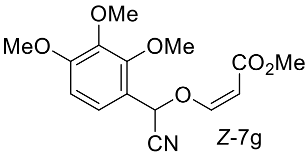
OMe
MeO
OMe
CO2Me
O
CN
Z-7g

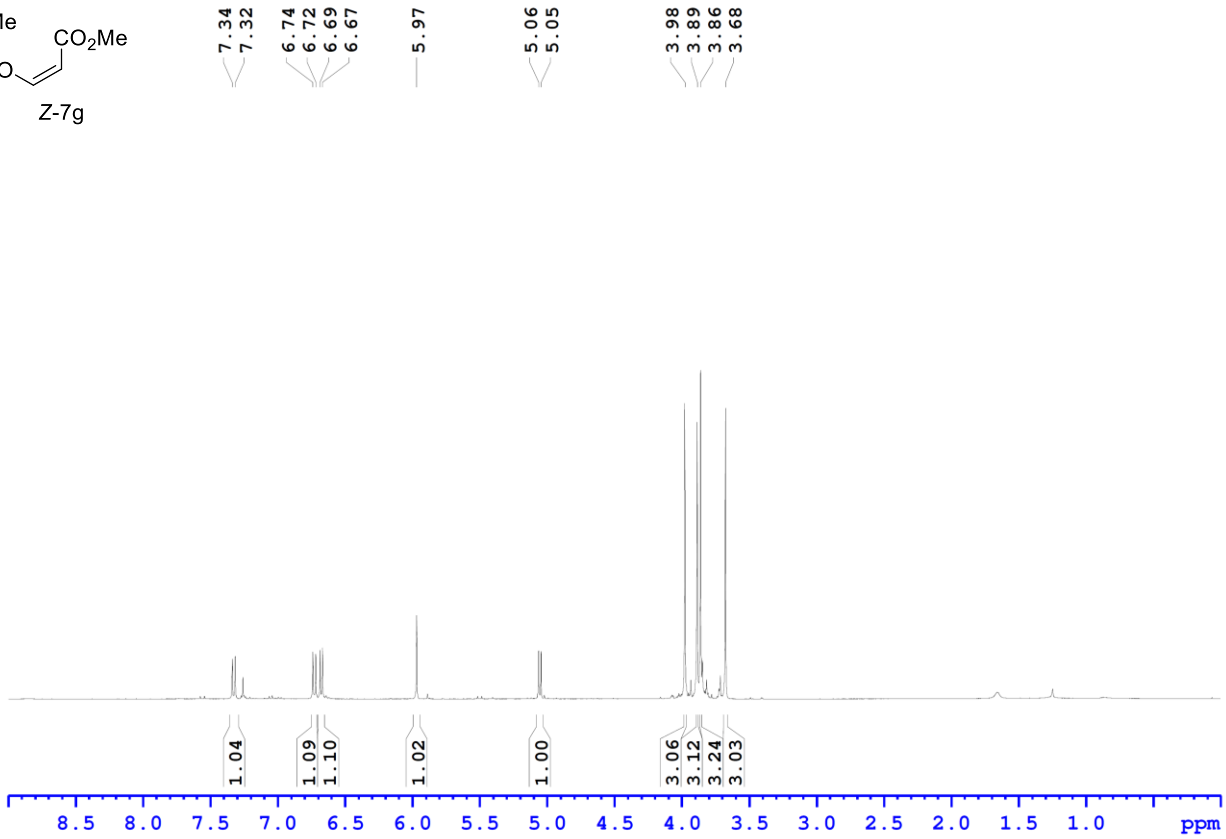
7.34
7.32
6.74
6.72
6.69
6.67
5.97
5.06
5.05
3.98
3.89
3.86
3.68
1.04
1.09
1.10
1.02
1.00
3.06
3.12
3.24
3.03
8.5 8.0 7.5 7.0 6.5 6.0 5.5 5.0 4.5 4.0 3.5 3.0 2.5 2.0 1.5 1.0 ppm

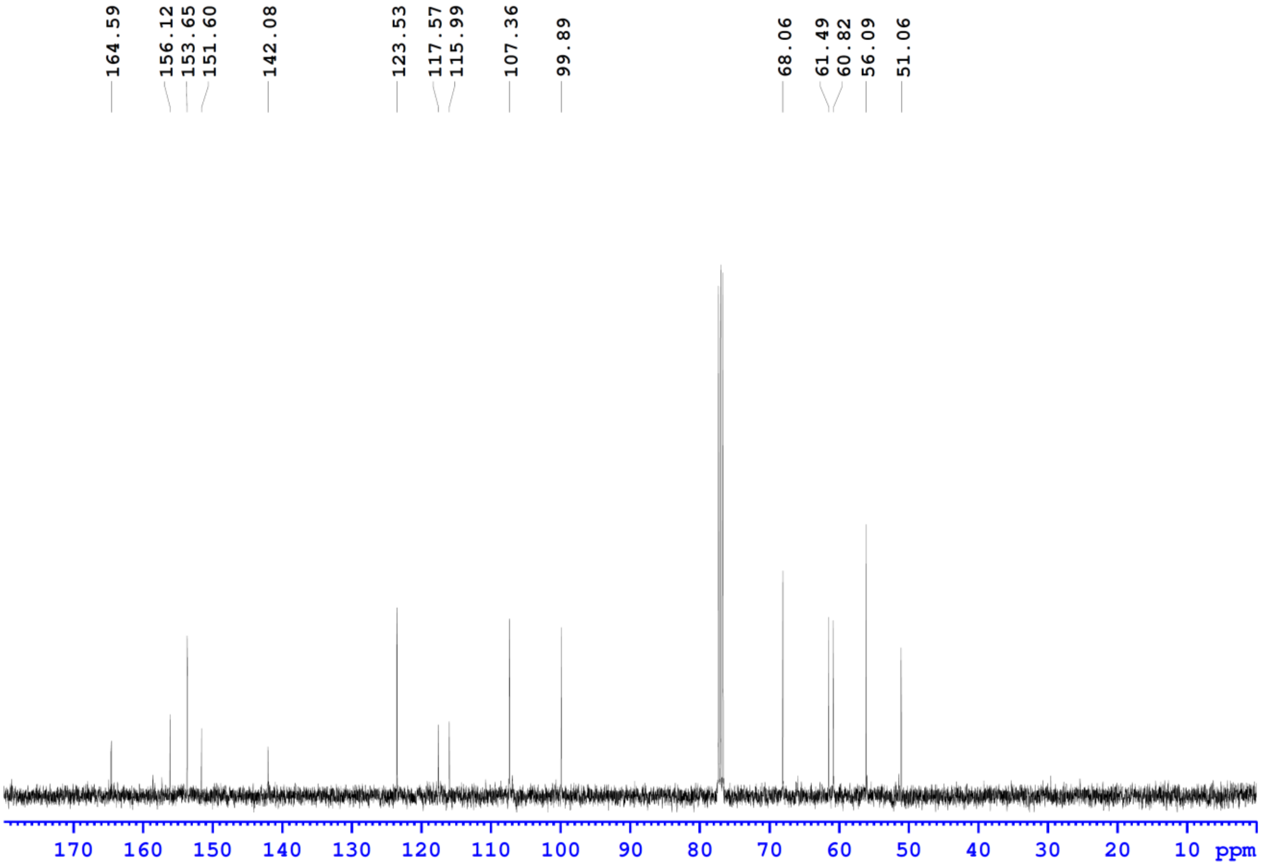
164.59
156.12
153.65
151.60
142.08
123.53
117.57
115.99
107.36
99.89
68.06
61.49
60.82
56.09
51.06
170 160 150 140 130 120 110 100 90 80 70 60 50 40 30 20 10 ppm

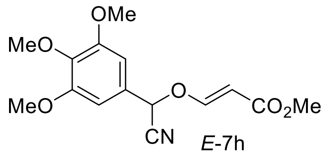
OMe
MeO
MeO
O
CO2Me
CN

E-7h

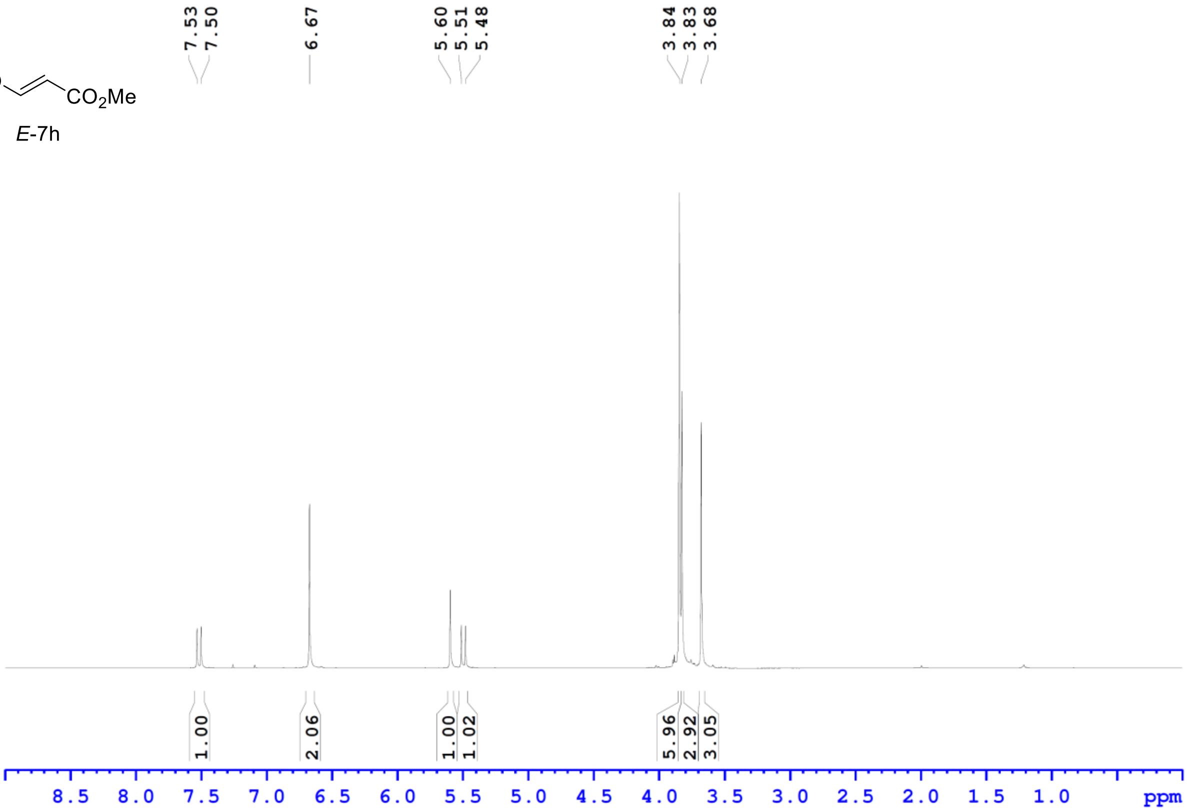
7.53
7.50
6.67
5.60
5.51
5.48
3.84
3.83
3.68
1.00
2.06
1.00
1.02
5.96
2.92
3.05
8.5 8.0 7.5 7.0 6.5 6.0 5.5 5.0 4.5 4.0 3.5 3.0 2.5 2.0 1.5 1.0 ppm

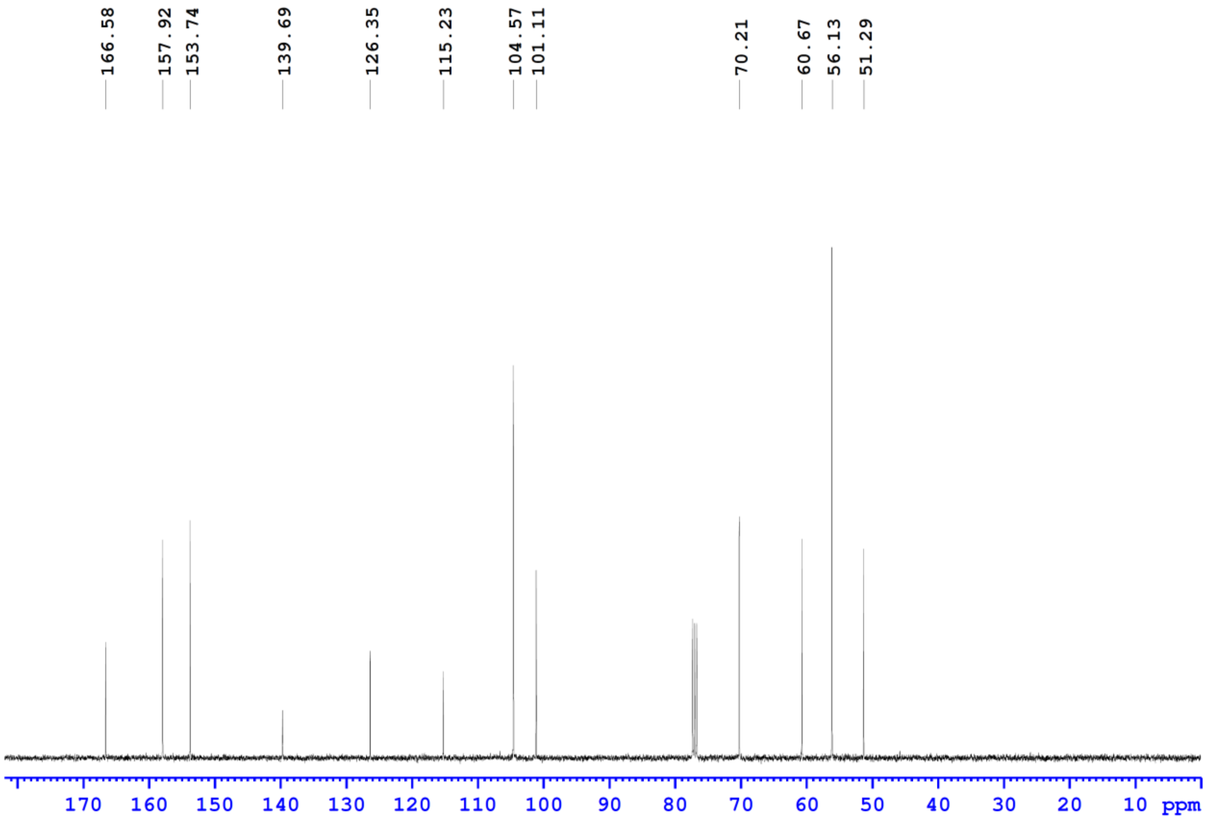
166.58
157.92
153.74
139.69
126.35
115.23
104.57
101.11
70.21
60.67
56.13
51.29
170 160 150 140 130 120 110 100 90 80 70 60 50 40 30 20 10 ppm

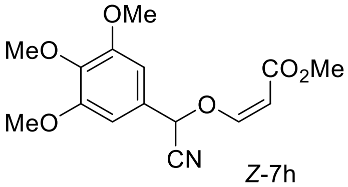
OMe
MeO
MeO
CO2Me
O
CN

Z-7h

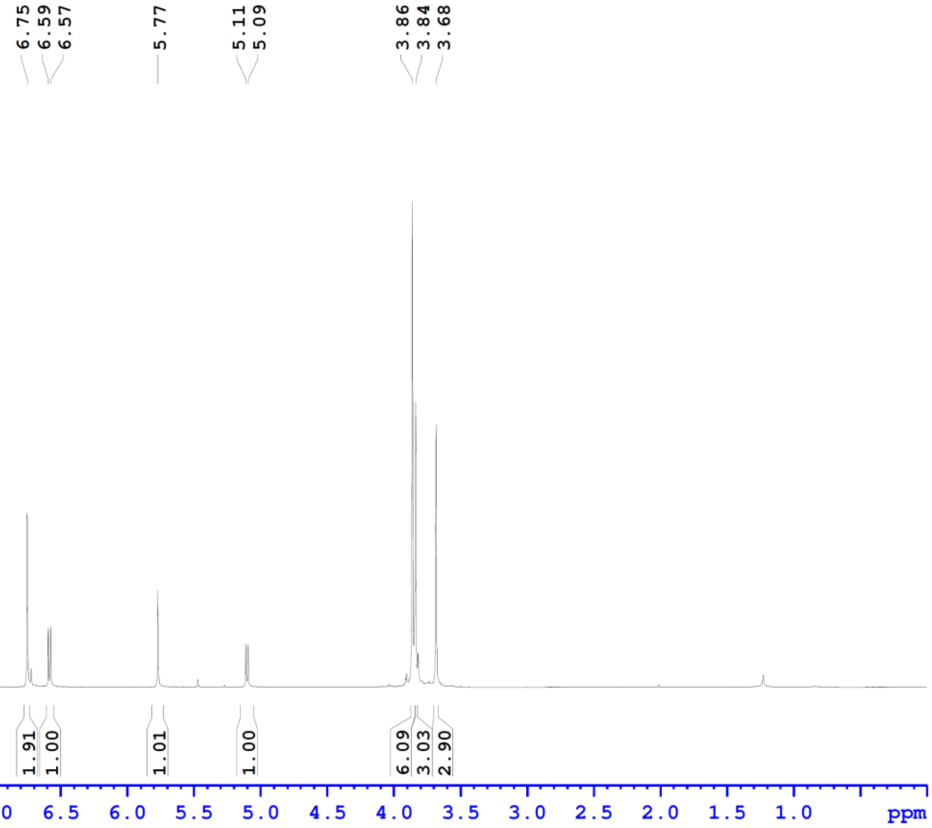
6.75
6.59
6.57
5.77
5.11
5.09
3.86
3.84
3.68
1.91
1.00
1.01
1.00
6.09
3.03
2.90
8.5 8.0 7.5 7.0 6.5 6.0 5.5 5.0 4.5 4.0 3.5 3.0 2.5 2.0 1.5 1.0 ppm

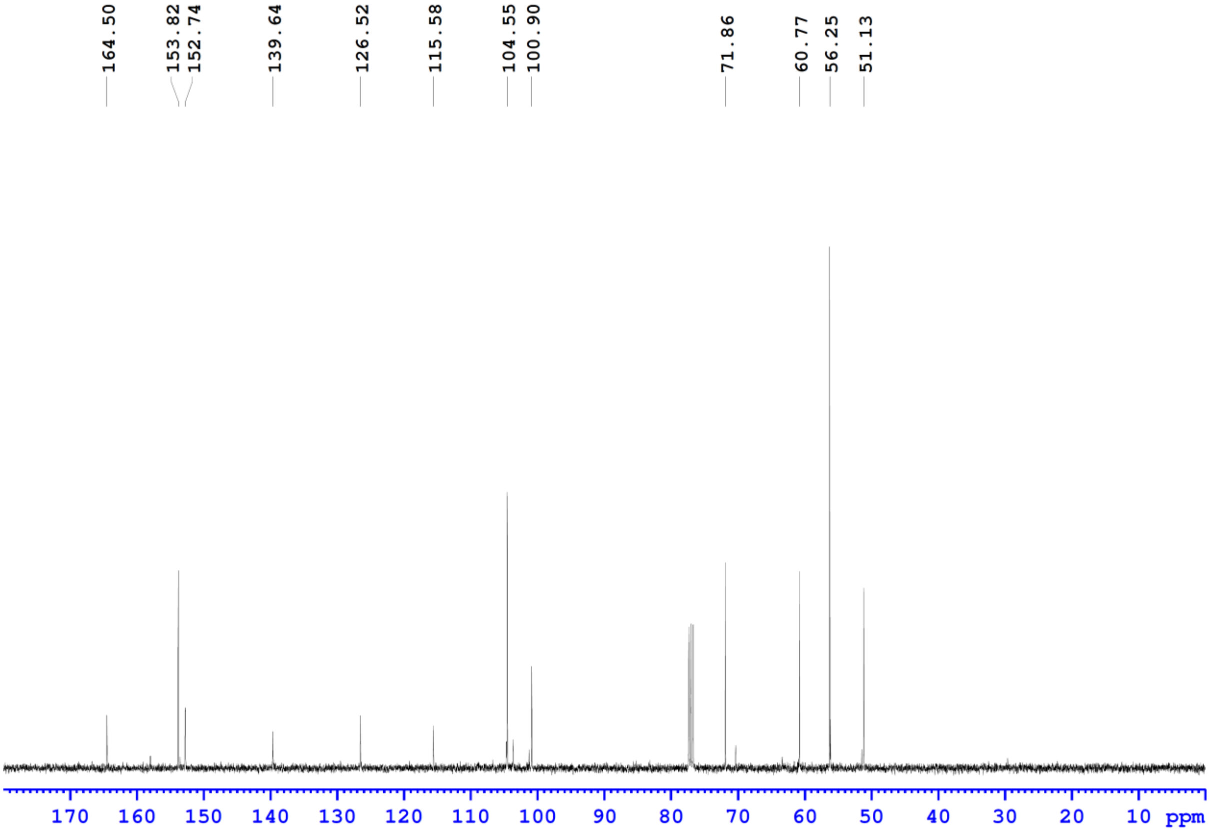
164.50
153.82
152.74
139.64
126.52
115.58
104.55
100.90
71.86
60.77
56.25
51.13
170 160 150 140 130 120 110 100 90 80 70 60 50 40 30 20 10 ppm

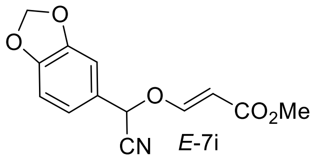
O
O
O
CO2Me
CN

E-7i

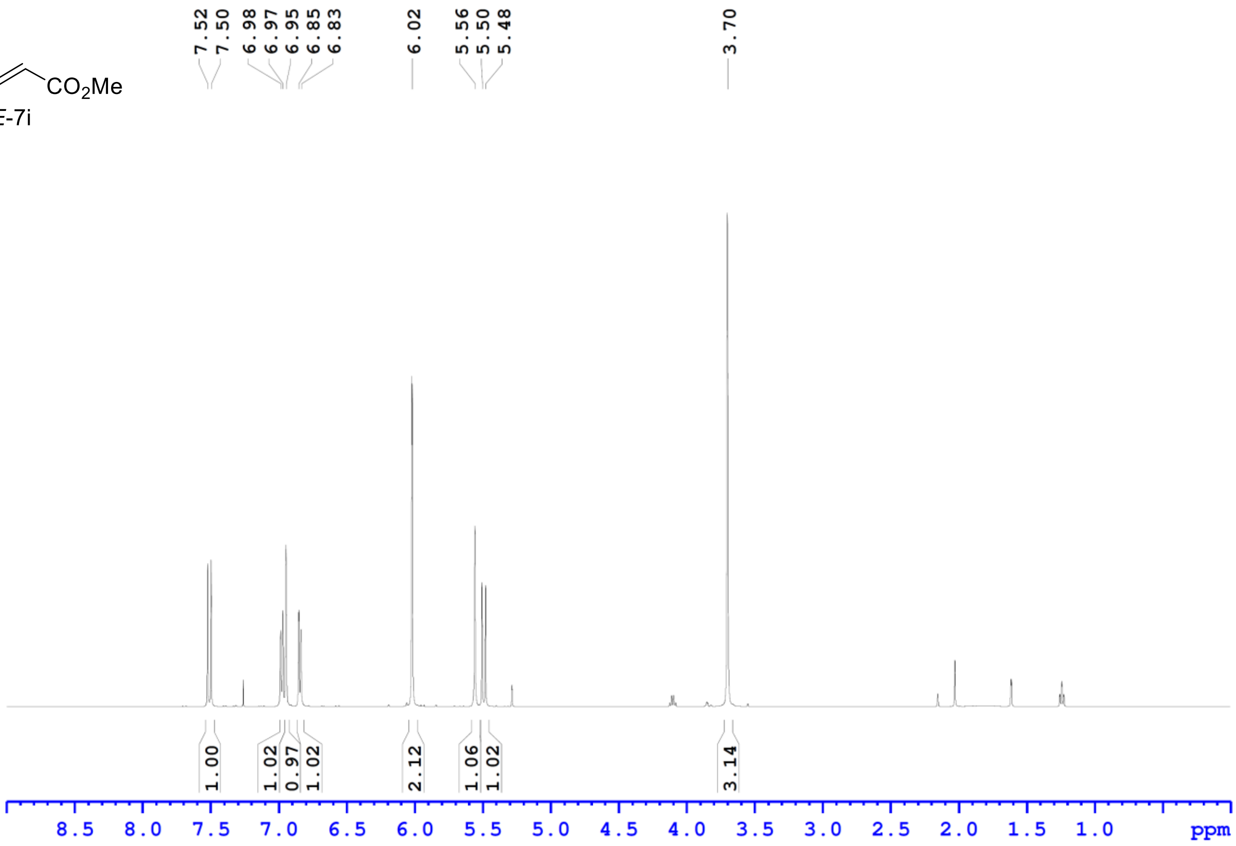
7.52
7.50
6.98
6.97
6.95
6.85
6.83
6.02
5.56
5.50
5.48
3.70
1.00
1.02
0.97
1.02
2.12
1.06
1.02
3.14
ppm

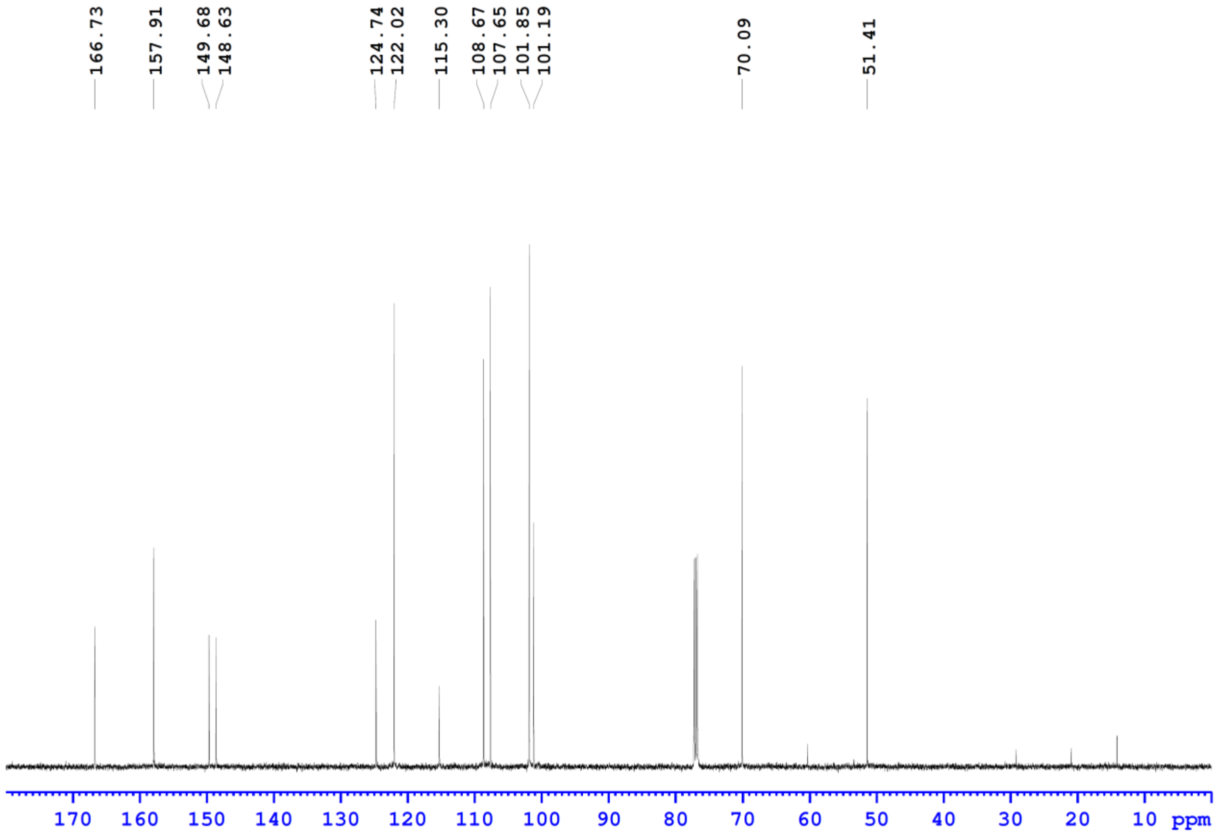
166.73
157.91
149.68
148.63
124.74
122.02
115.30
108.67
107.65
101.85
101.19
70.09
51.41
ppm

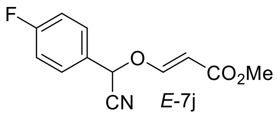
F
O
CO2Me
CN
E-7j

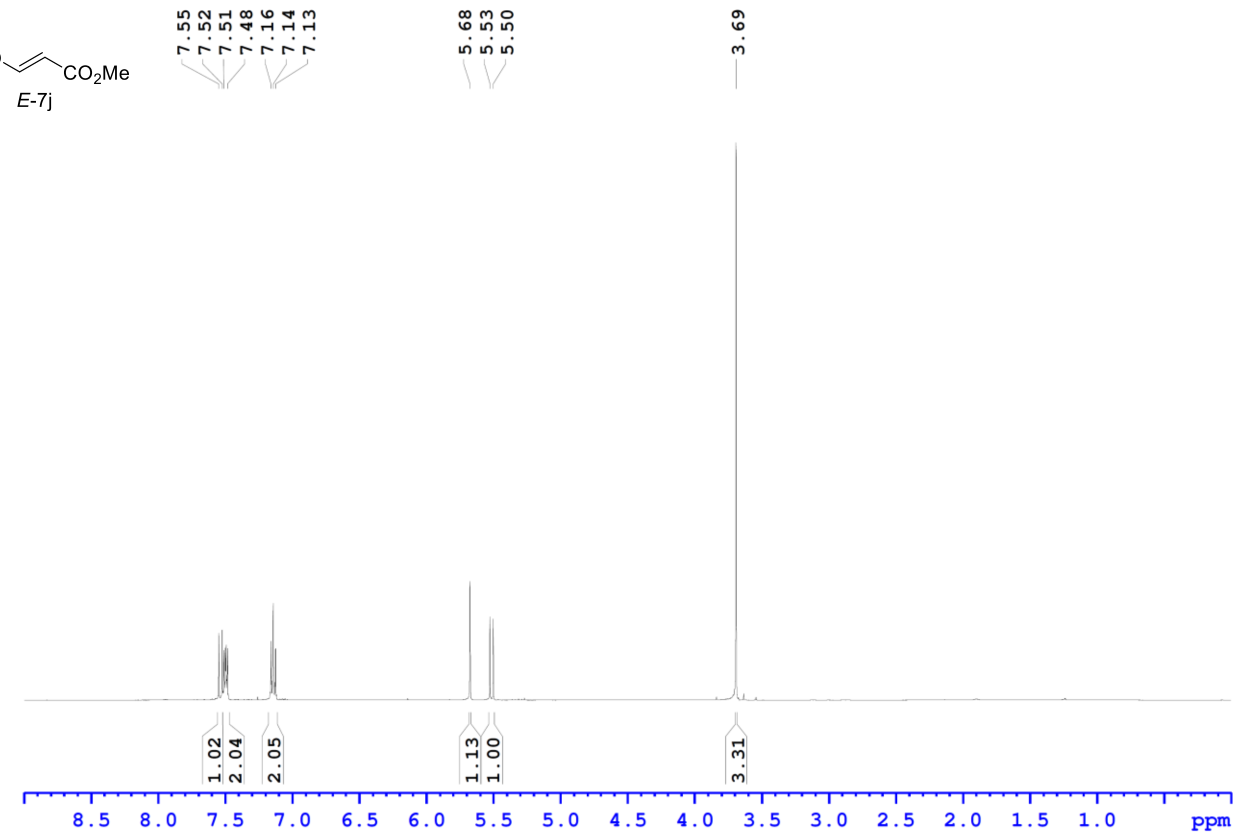
7.55
7.52
7.51
7.48
7.16
7.14
7.13
5.68
5.53
5.50
3.69
1.02
2.04
2.05
1.13
1.00
3.31
8.5 8.0 7.5 7.0 6.5 6.0 5.5 5.0 4.5 4.0 3.5 3.0 2.5 2.0 1.5 1.0 ppm

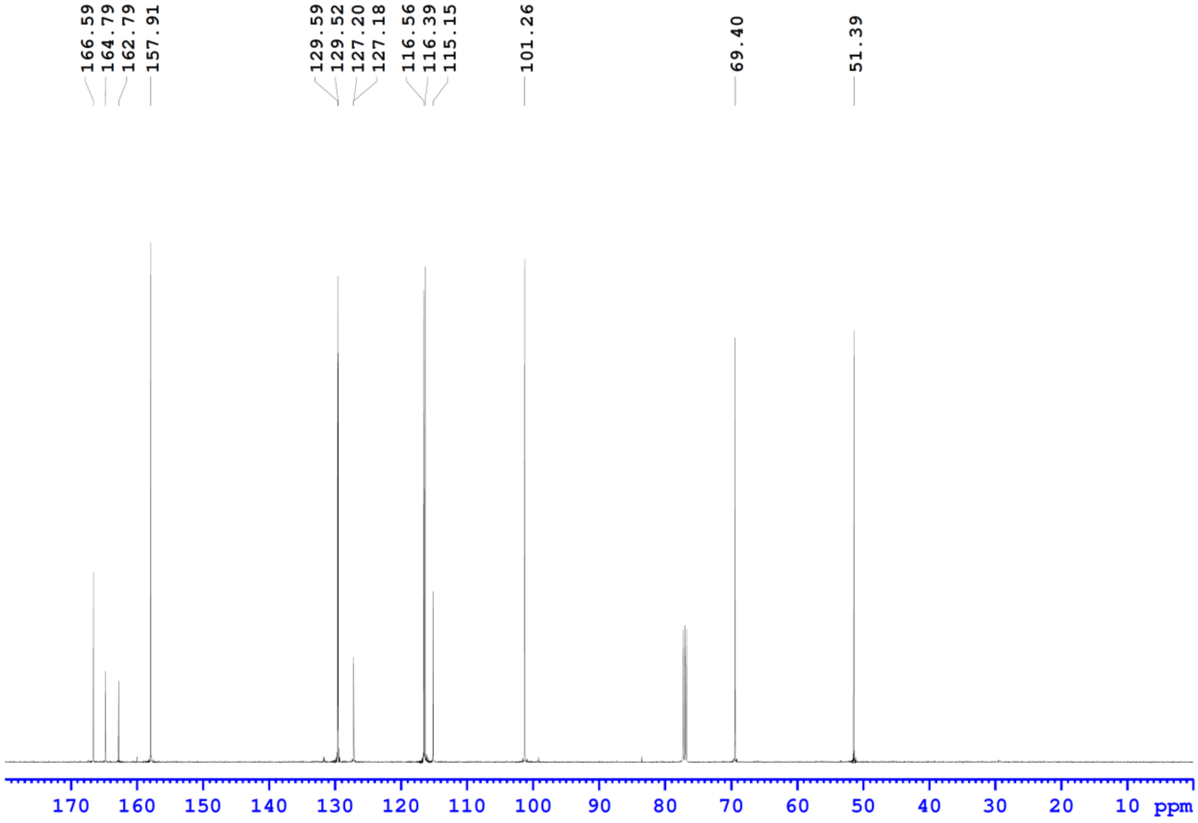
166.59
164.79
162.79
157.91
129.59
129.52
127.20
127.18
116.56
116.39
115.15
101.26
69.40
51.39
170 160 150 140 130 120 110 100 90 80 70 60 50 40 30 20 10 ppm

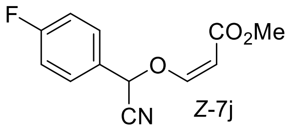
F
CO2Me
O
CN

Z-7j

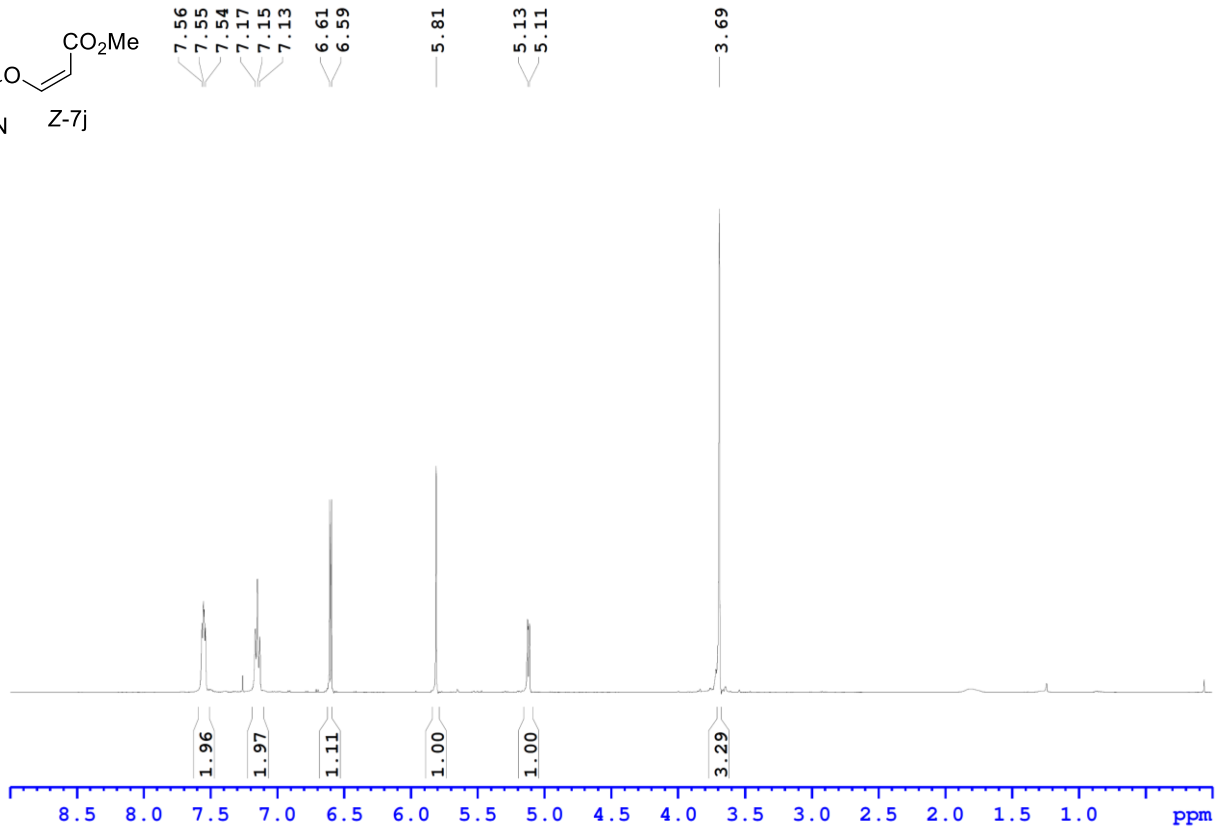
7.56
7.55
7.54
7.17
7.15
7.13
6.61
6.59
5.81
5.13
5.11
3.69
1.96
1.97
1.11
1.00
1.00
3.29
8.5 8.0 7.5 7.0 6.5 6.0 5.5 5.0 4.5 4.0 3.5 3.0 2.5 2.0 1.5 1.0 ppm

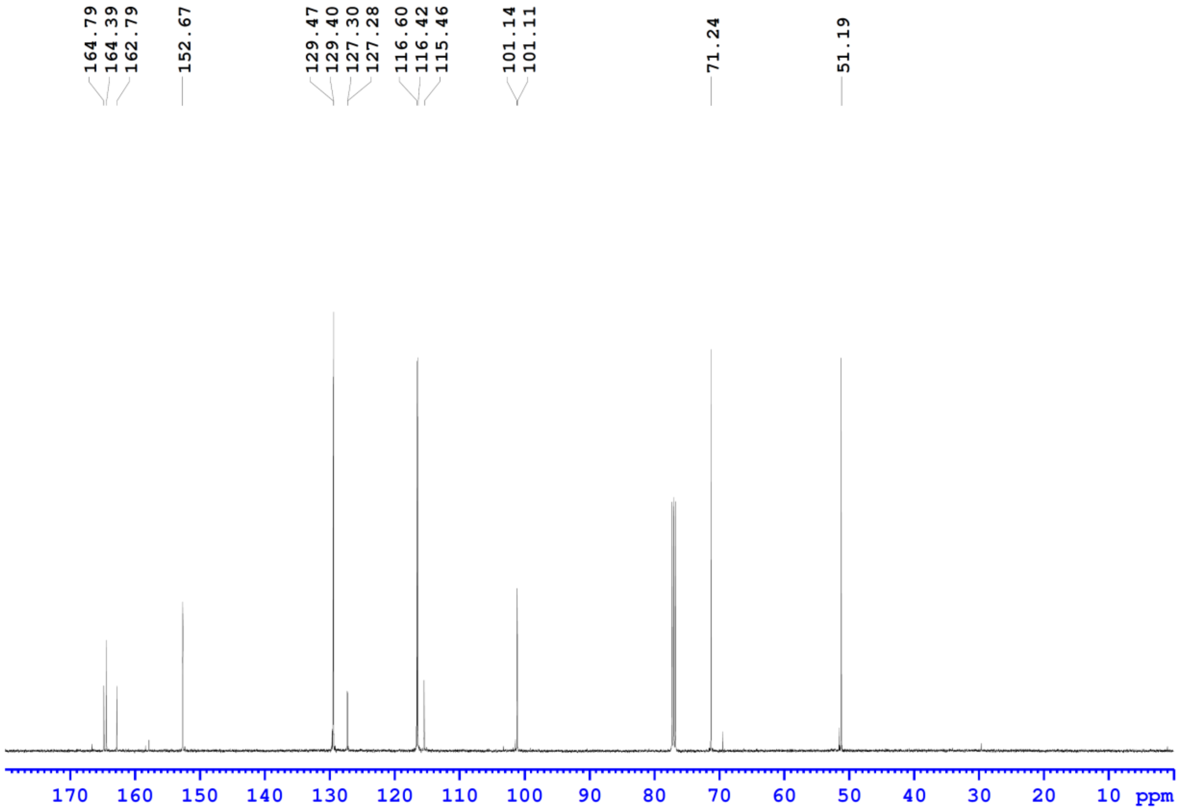
164.79
164.39
162.79
152.67
129.47
129.40
127.30
127.28
116.60
116.42
115.46
101.14
101.11
71.24
51.19
170 160 150 140 130 120 110 100 90 80 70 60 50 40 30 20 10 ppm

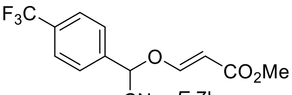
F3C
O
CO2Me
CN

E-7k

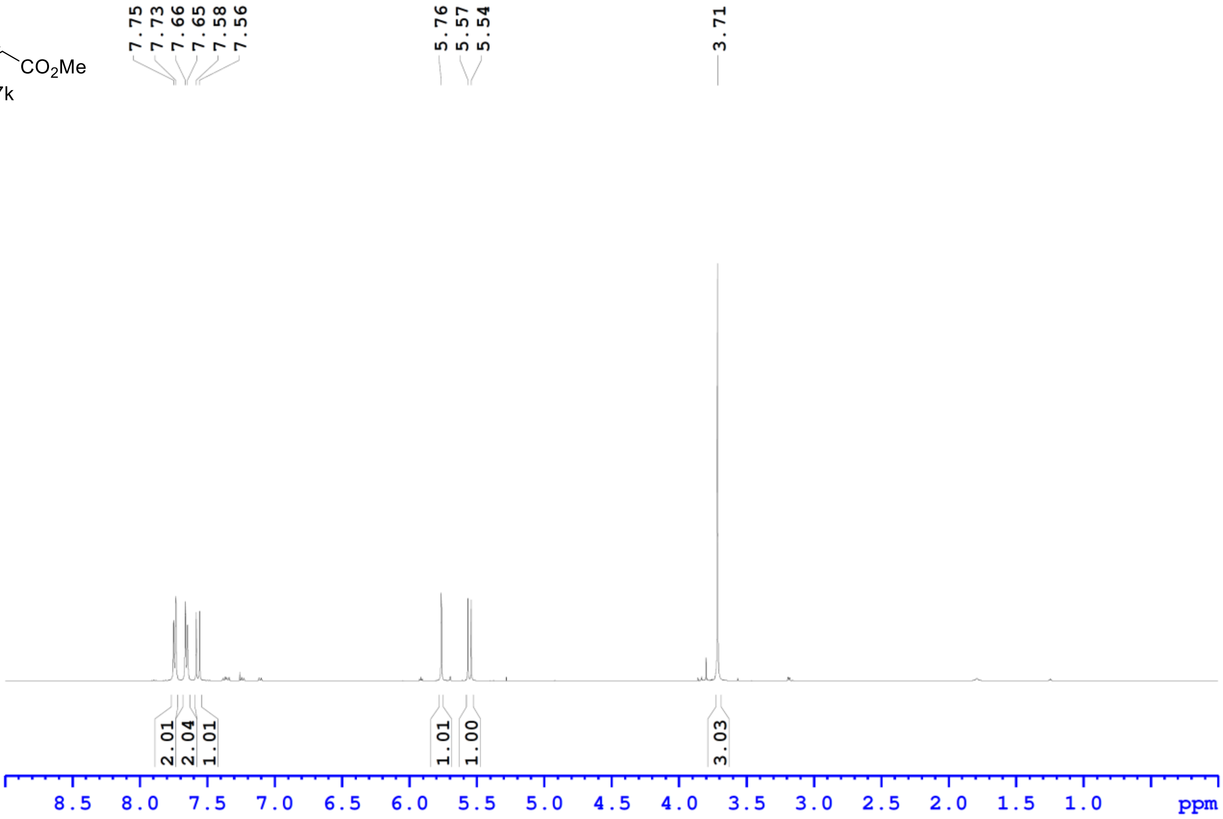
7.75
7.73
7.66
7.65
7.58
7.56
5.76
5.57
5.54
3.71
2.01
2.04
1.01
1.01
1.00
3.03
8.5 8.0 7.5 7.0 6.5 6.0 5.5 5.0 4.5 4.0 3.5 3.0 2.5 2.0 1.5 1.0 ppm

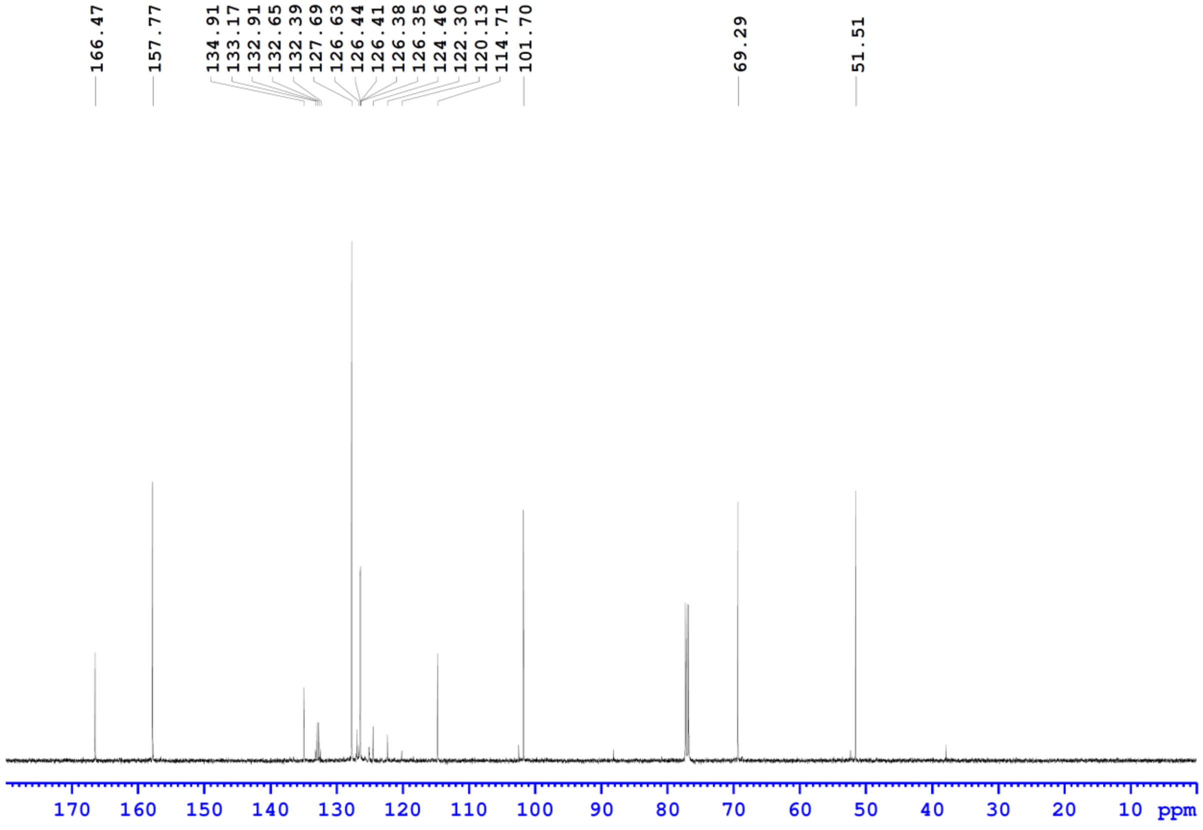
166.47
157.77
134.91
133.17
132.91
132.65
132.39
127.69
126.63
126.44
126.41
126.38
126.35
124.46
122.30
120.13
114.71
101.70
69.29
51.51
170 160 150 140 130 120 110 100 90 80 70 60 50 40 30 20 10 ppm

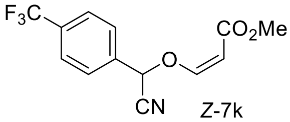
F3C
CO2Me
O
CN

Z-7k

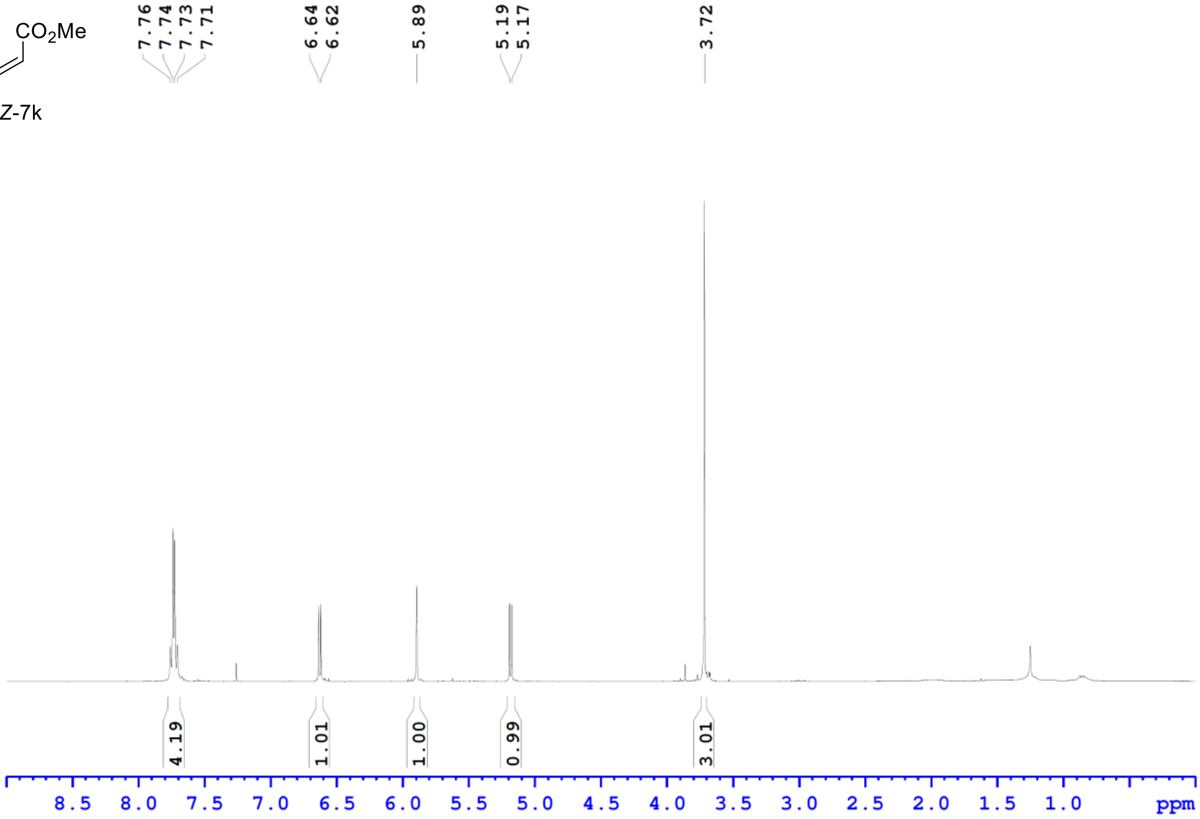
7.76
7.74
7.73
7.71
6.64
6.62
5.89
5.19
5.17
3.72
4.19
1.01
1.00
0.99
3.01
8.5 8.0 7.5 7.0 6.5 6.0 5.5 5.0 4.5 4.0 3.5 3.0 2.5 2.0 1.5 1.0 ppm

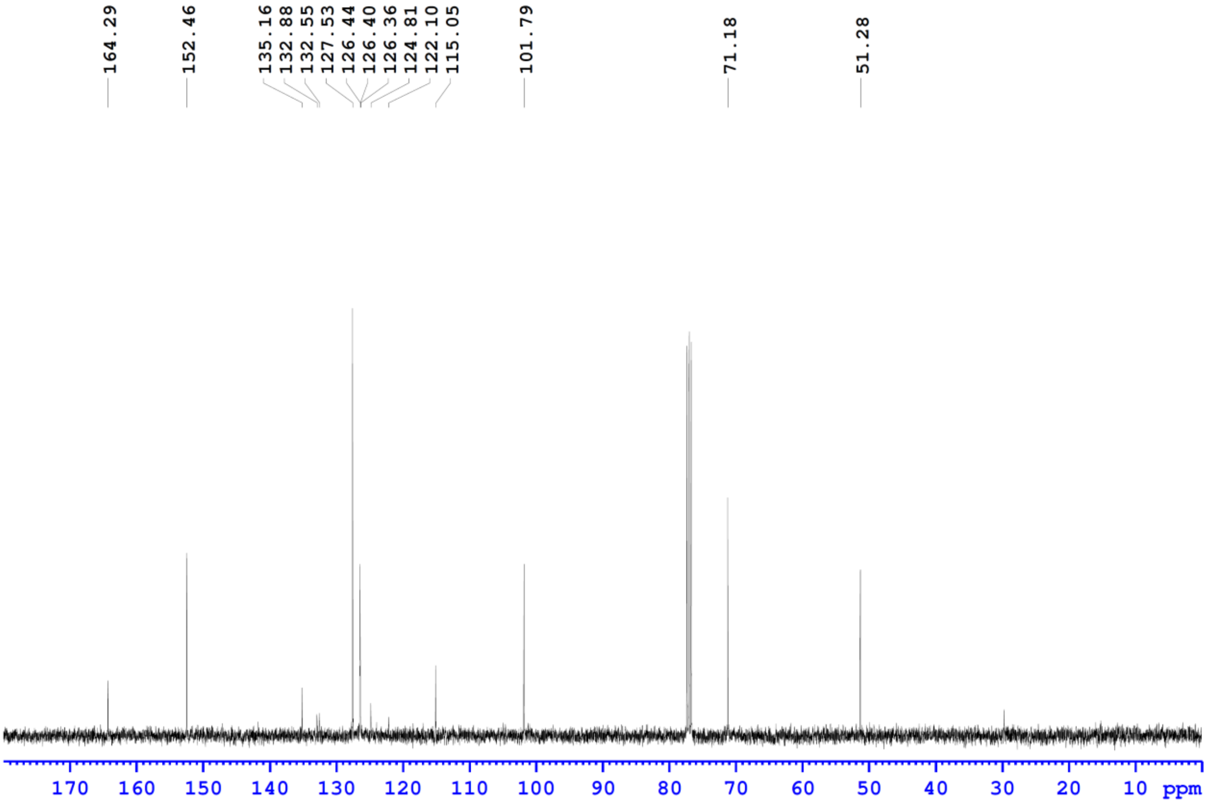
164.29
152.46
135.16
132.88
132.55
127.53
126.44
126.40
126.36
124.81
122.10
115.05
101.79
71.18
51.28
170 160 150 140 130 120 110 100 90 80 70 60 50 40 30 20 10 ppm

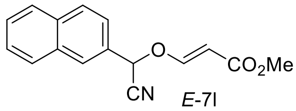
CO2Me
CN
O

E-7l

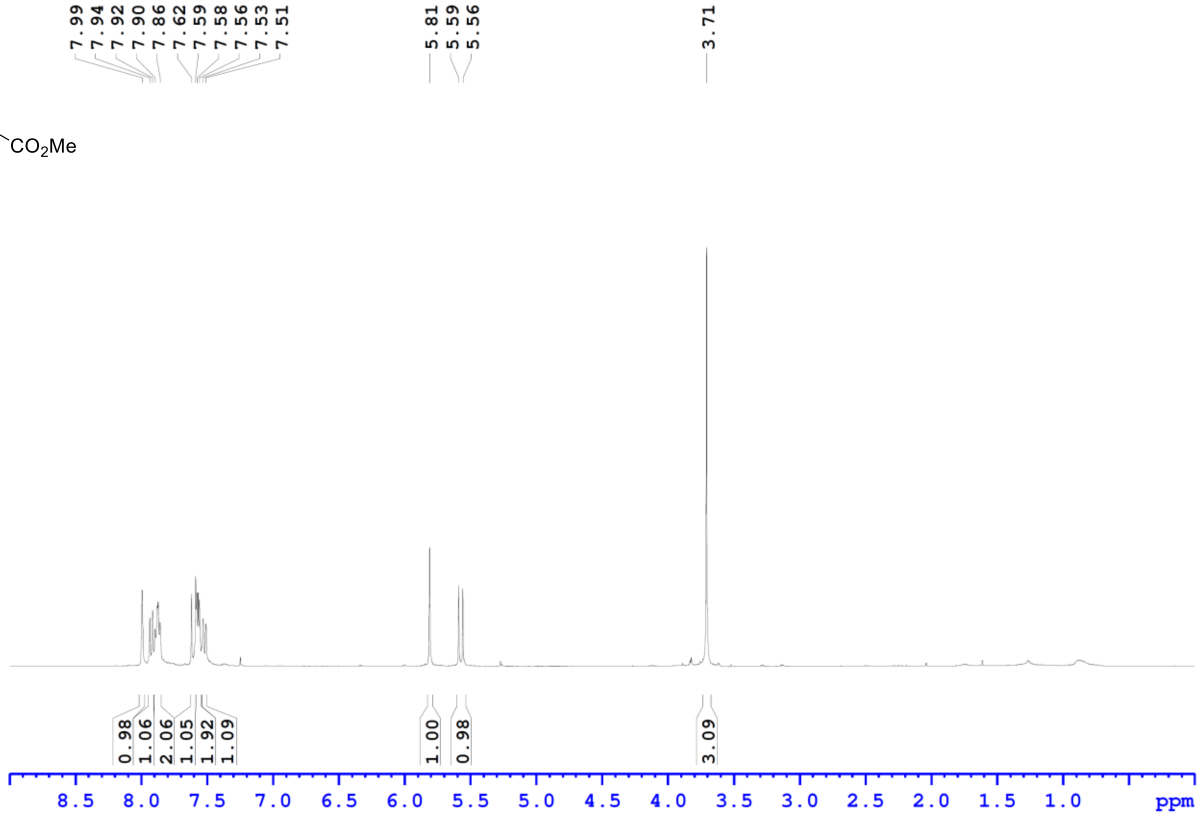
7.99
7.94
7.92
7.90
7.86
7.62
7.59
7.58
7.56
7.53
7.51
5.81
5.59
5.56
3.71
0.98
1.06
2.06
1.05
1.92
1.09
1.00
0.98
3.09
8.5 8.0 7.5 7.0 6.5 6.0 5.5 5.0 4.5 4.0 3.5 3.0 2.5 2.0 1.5 1.0 ppm

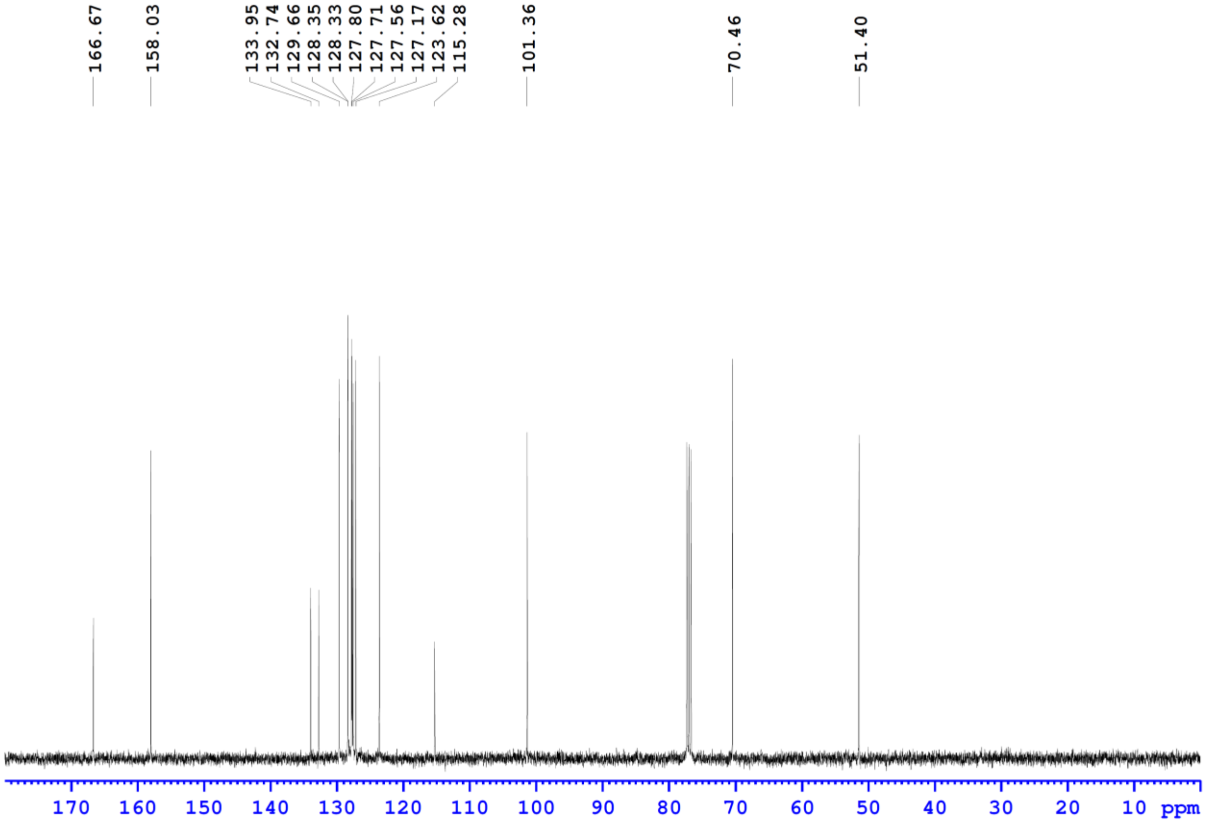
166.67
158.03
133.95
132.74
129.66
128.35
128.33
127.80
127.71
127.56
127.17
123.62
115.28
101.36
70.46
51.40
170 160 150 140 130 120 110 100 90 80 70 60 50 40 30 20 10 ppm

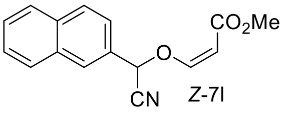
CO2Me
O
CN

Z-7l

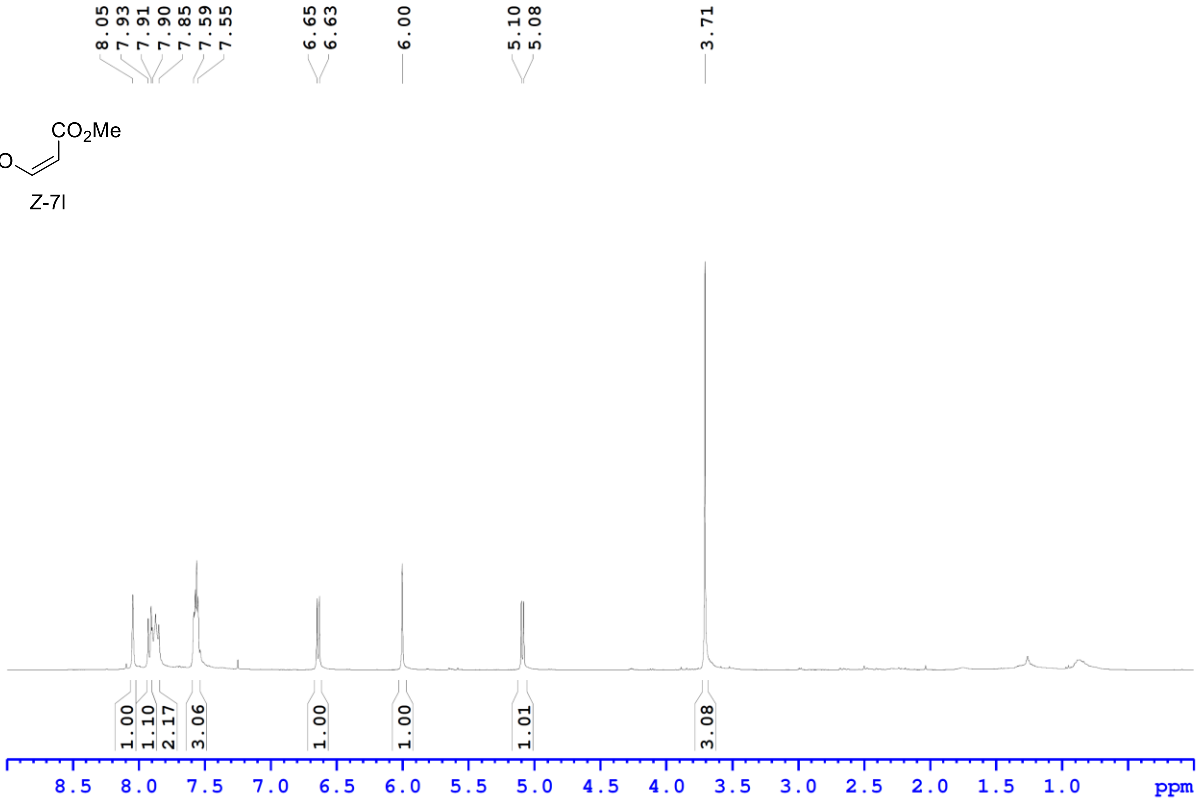
8.05
7.93
7.91
7.90
7.85
7.59
7.55
6.65
6.63
6.00
5.10
5.08
3.71
1.00
1.10
2.17
3.06
1.00
1.00
1.01
3.08
8.5 8.0 7.5 7.0 6.5 6.0 5.5 5.0 4.5 4.0 3.5 3.0 2.5 2.0 1.5 1.0 ppm

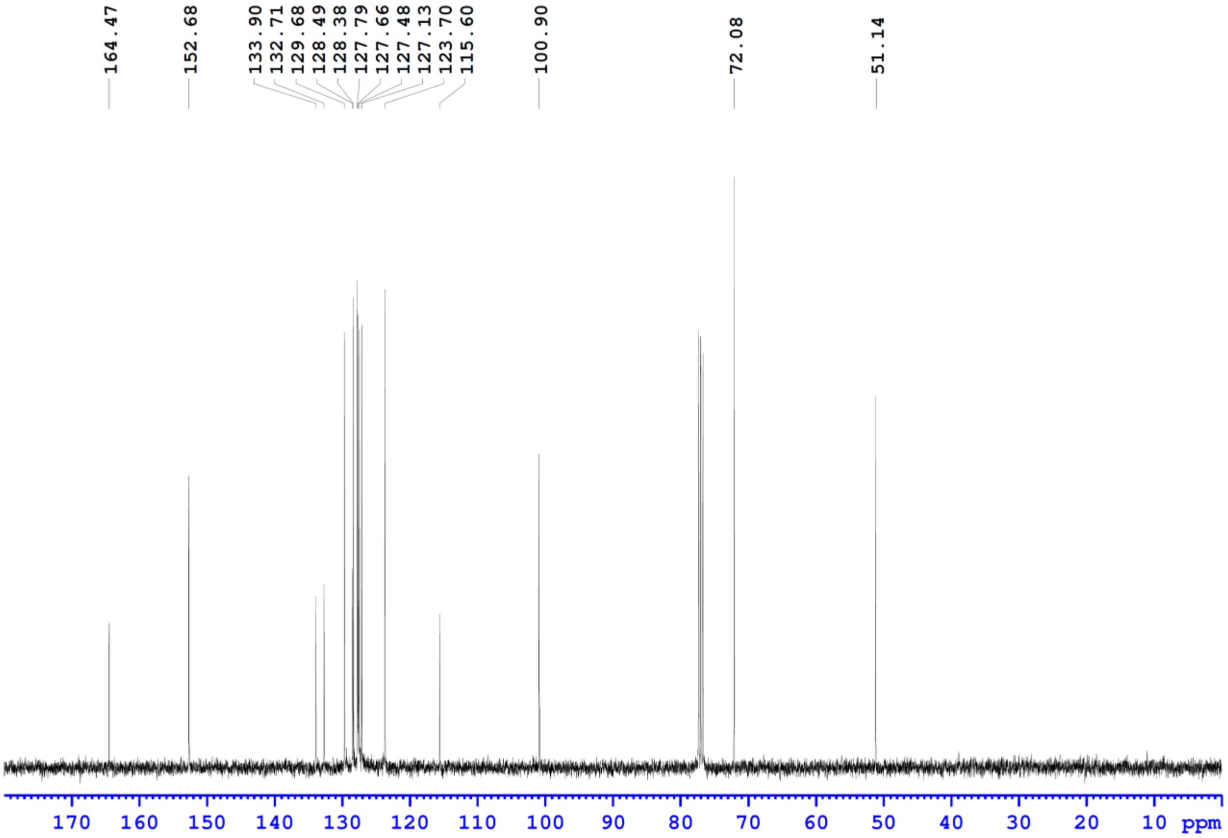
164.47
152.68
133.90
132.71
129.68
128.49
128.38
127.79
127.66
127.48
127.13
123.70
115.60
100.90
72.08
51.14
170 160 150 140 130 120 110 100 90 80 70 60 50 40 30 20 10 ppm

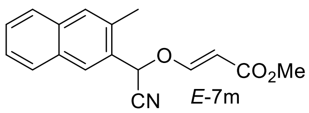

E-7m

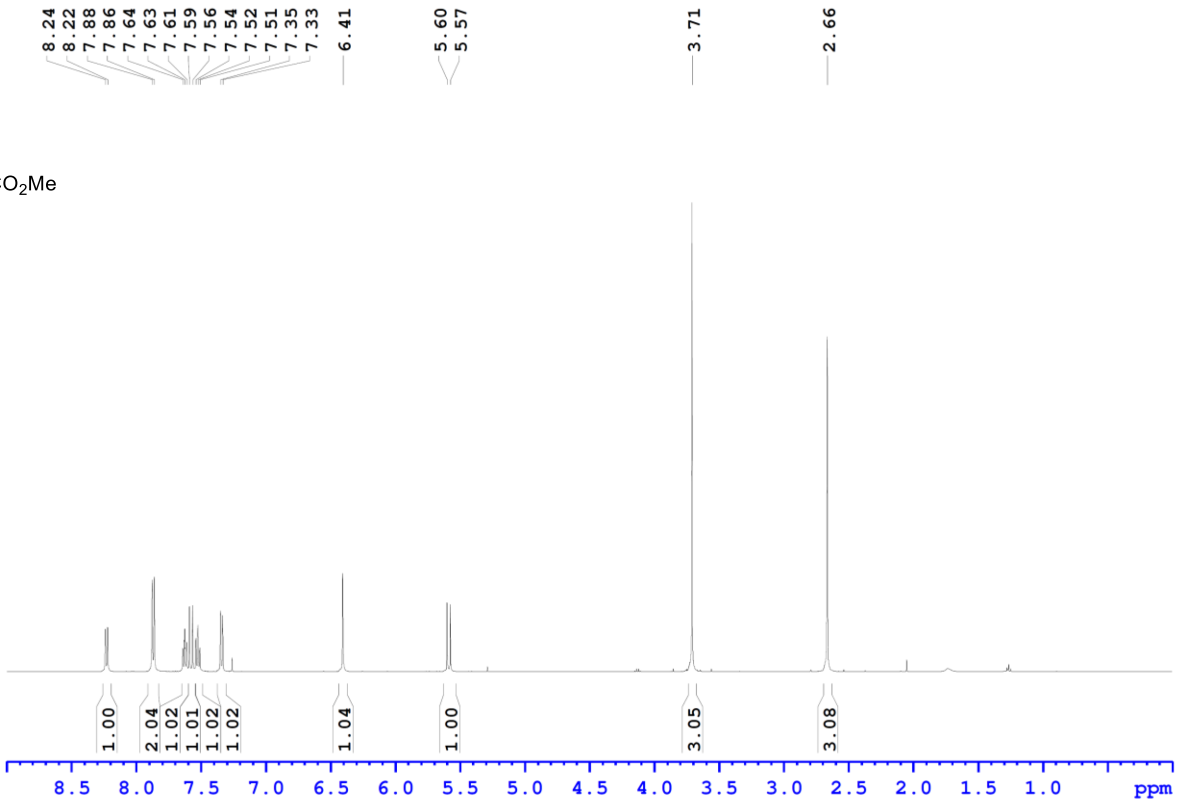

8.24
8.22
7.88
7.86
7.64
7.63
7.61
7.59
7.56
7.54
7.52
7.51
7.35
7.33
6.41
5.60
5.57
3.71
2.66
1.00
2.04
1.02
1.01
1.02
1.02
1.04
1.00
3.05
3.08
8.5 8.0 7.5 7.0 6.5 6.0 5.5 5.0 4.5 4.0 3.5 3.0 2.5 2.0 1.5 1.0 ppm

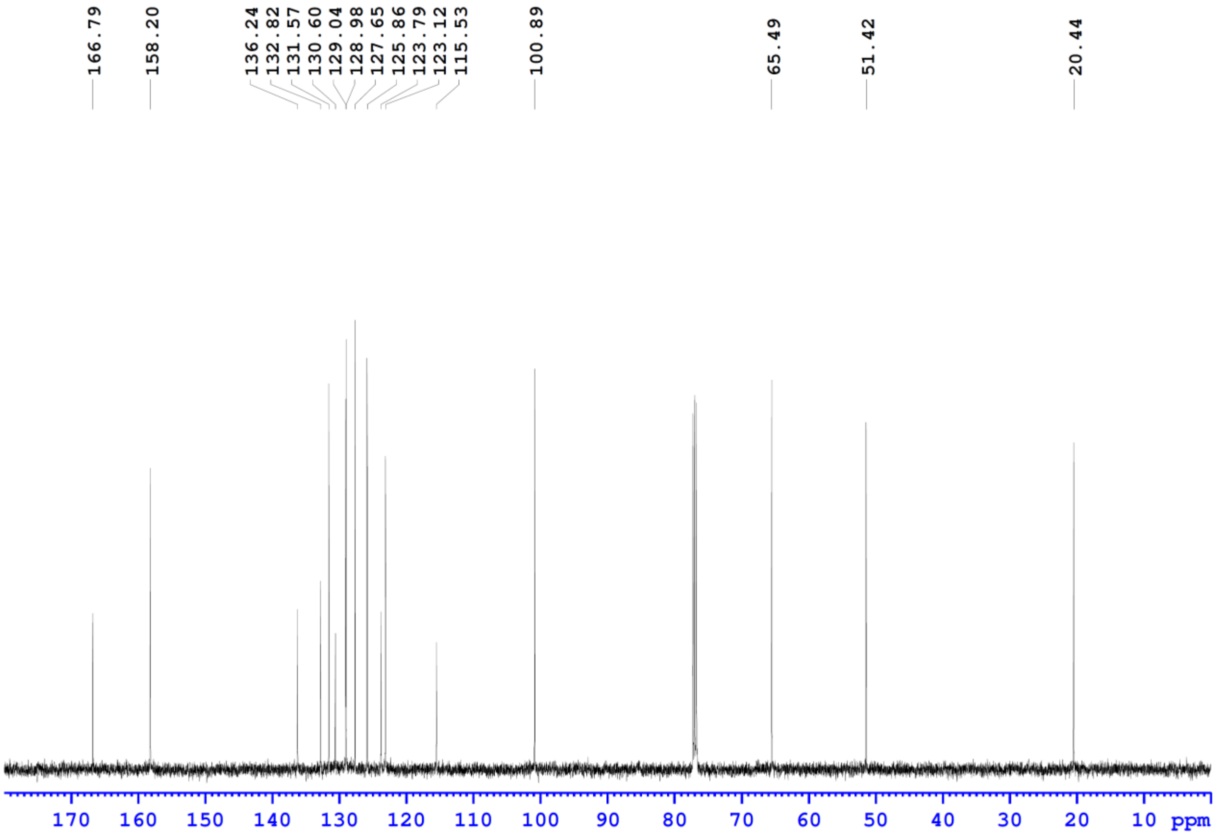

166.79
158.20
136.24
132.82
131.57
130.60
129.04
128.98
127.65
125.86
123.79
123.12
115.53
100.89
65.49
51.42
20.44
170 160 150 140 130 120 110 100 90 80 70 60 50 40 30 20 10 ppm

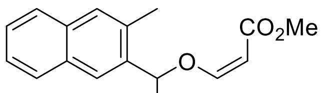
CO2Me
O
CN

Z-7m

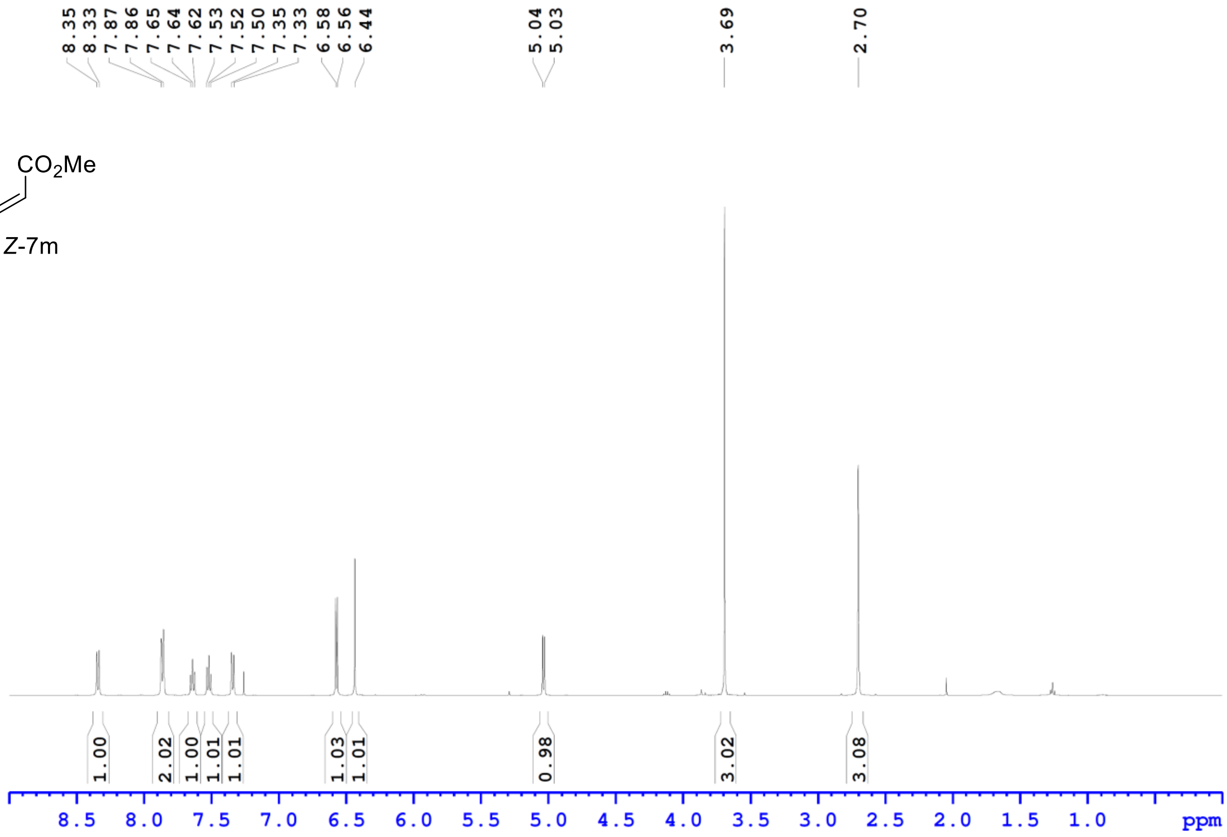
8.35 8.33 7.87 7.86 7.65 7.64 7.62 7.53 7.52 7.50 7.35 7.33 6.58 6.56 6.44
5.04 5.03
3.69
2.70
1.00 2.02 1.00 1.01 1.01 1.03 1.01 0.98 3.02 3.08
8.5 8.0 7.5 7.0 6.5 6.0 5.5 5.0 4.5 4.0 3.5 3.0 2.5 2.0 1.5 1.0 ppm

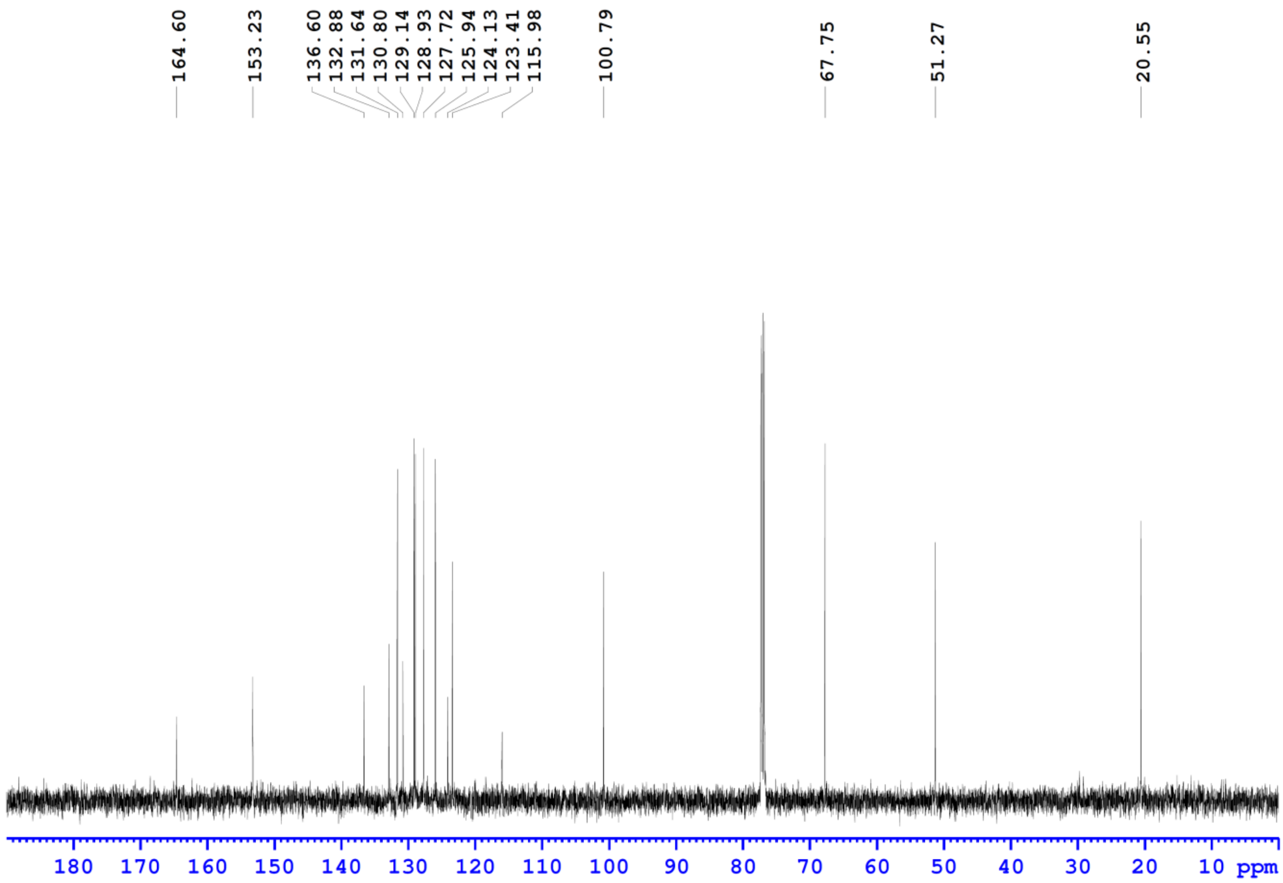
164.60
153.23
136.60 132.88 131.64 130.80 129.14 128.93 127.72 125.94 124.13 123.41 115.98
100.79
67.75
51.27
20.55
180 170 160 150 140 130 120 110 100 90 80 70 60 50 40 30 20 10 ppm

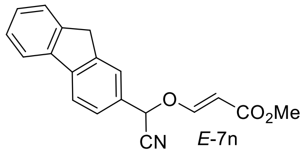
CO2Me
CN

E-7n

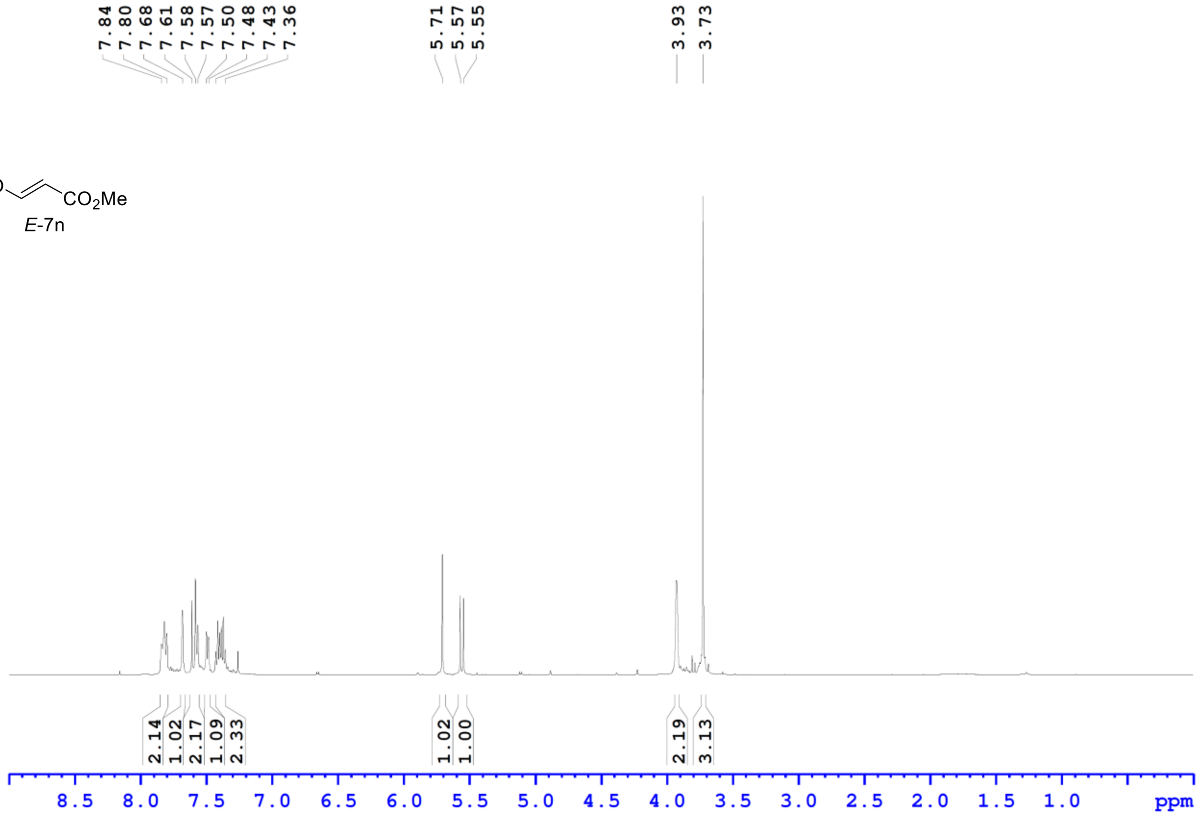
7.84 7.80 7.68 7.61 7.58 7.57 7.50 7.48 7.43 7.36
5.71 5.57 5.55
3.93 3.73
2.14 1.02 2.17 1.09 2.33 1.02 1.00 2.19 3.13
8.5 8.0 7.5 7.0 6.5 6.0 5.5 5.0 4.5 4.0 3.5 3.0 2.5 2.0 1.5 1.0 ppm

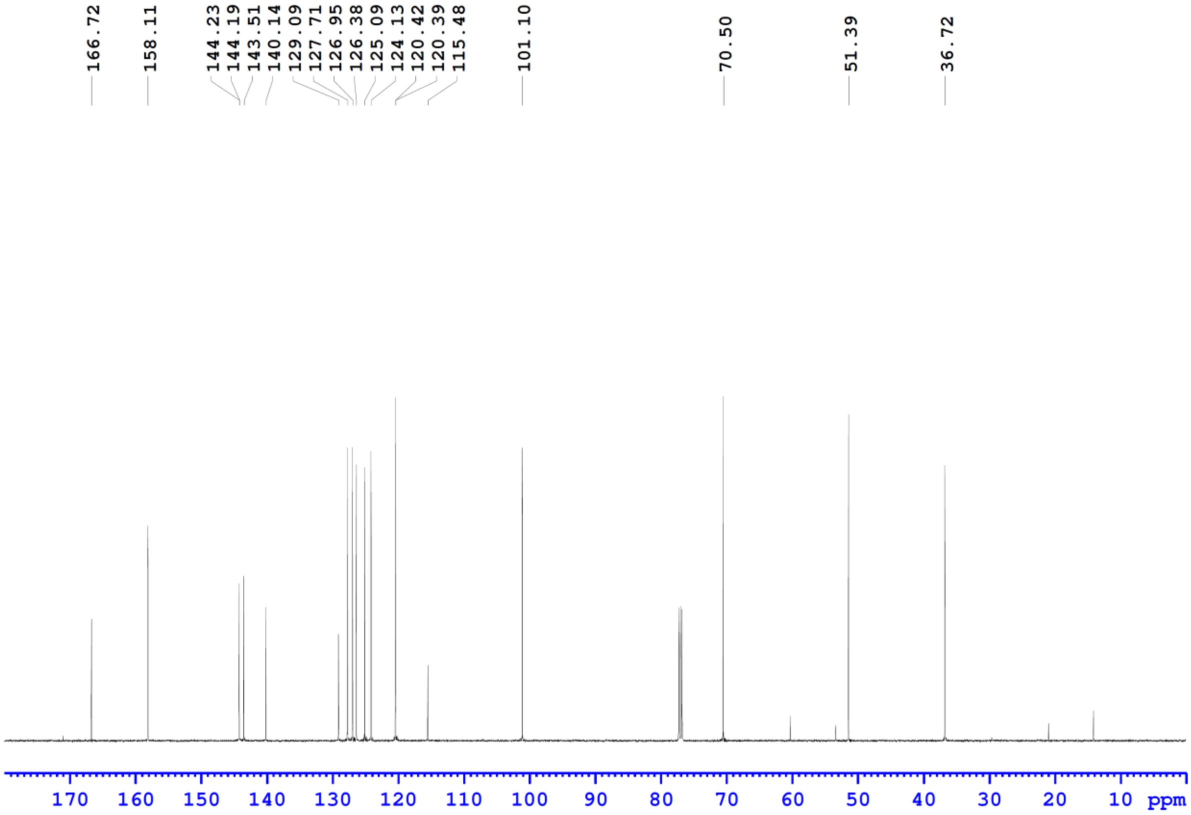
166.72
158.11
144.23 144.19 143.51 140.14 129.09 127.71 126.95 126.38 125.09 124.13 120.42 120.39 115.48
101.10
70.50
51.39
36.72
170 160 150 140 130 120 110 100 90 80 70 60 50 40 30 20 10 ppm

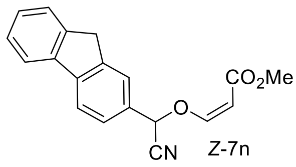
CO2Me
CN
Z-7n

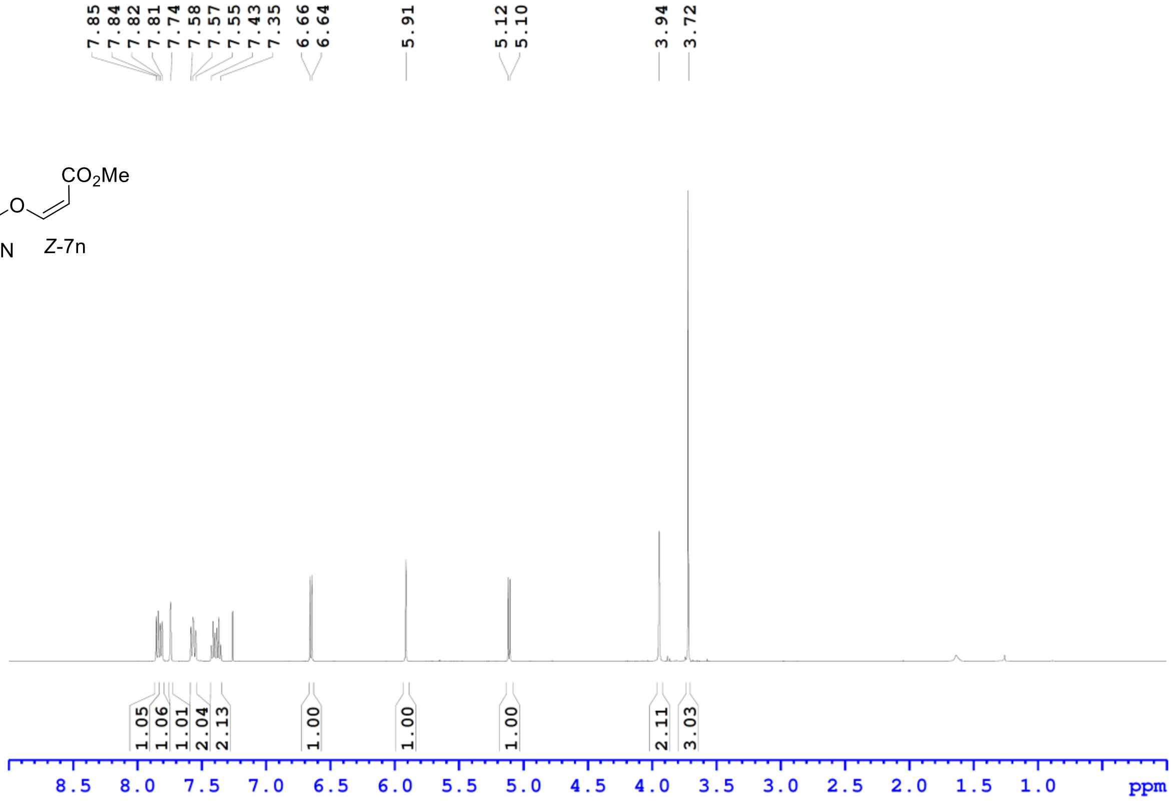
7.85
7.84
7.82
7.81
7.74
7.58
7.57
7.55
7.43
7.35
6.66
6.64
5.91
5.12
5.10
3.94
3.72
1.05
1.06
1.01
2.04
2.13
1.00
1.00
1.00
2.11
3.03
8.5 8.0 7.5 7.0 6.5 6.0 5.5 5.0 4.5 4.0 3.5 3.0 2.5 2.0 1.5 1.0 ppm

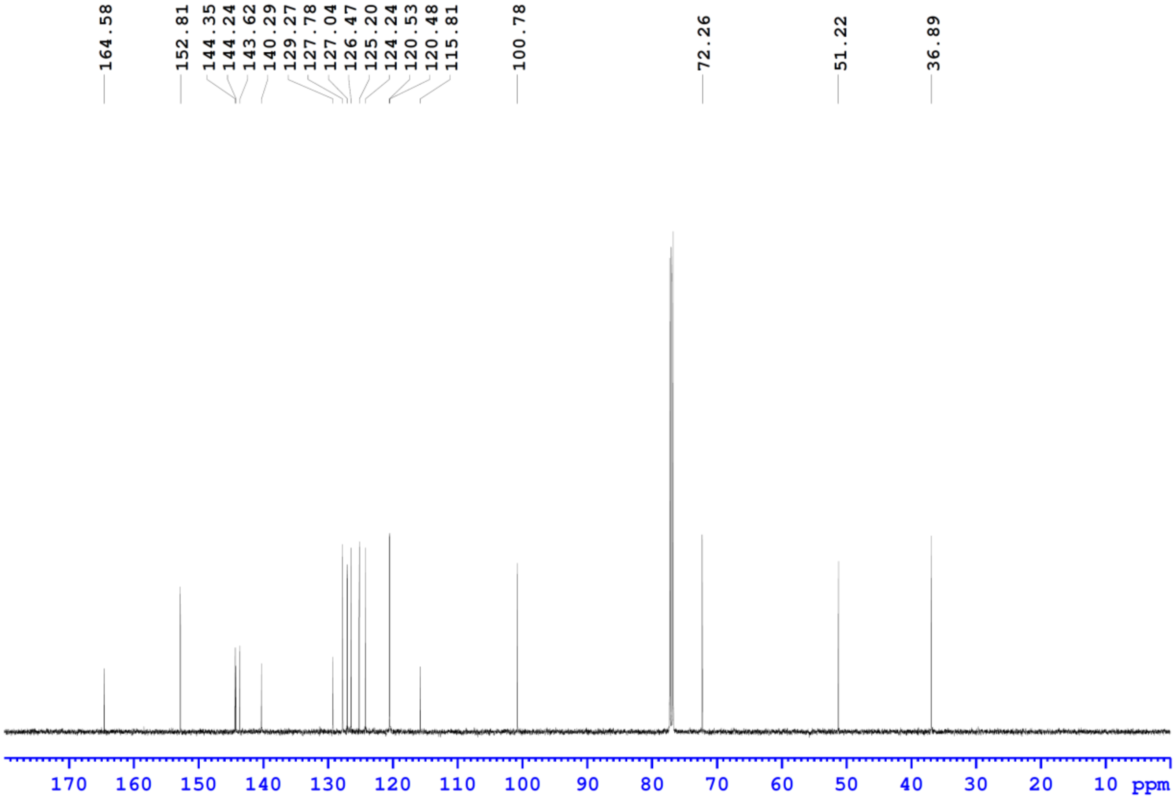
164.58
152.81
144.35
144.24
143.62
140.29
129.27
127.78
127.04
126.47
125.20
124.24
120.53
120.48
115.81
100.78
72.26
51.22
36.89
170 160 150 140 130 120 110 100 90 80 70 60 50 40 30 20 10 ppm

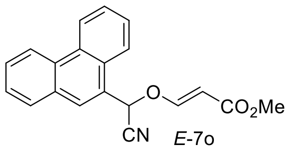
CO$_2$Me
CN

E-7o

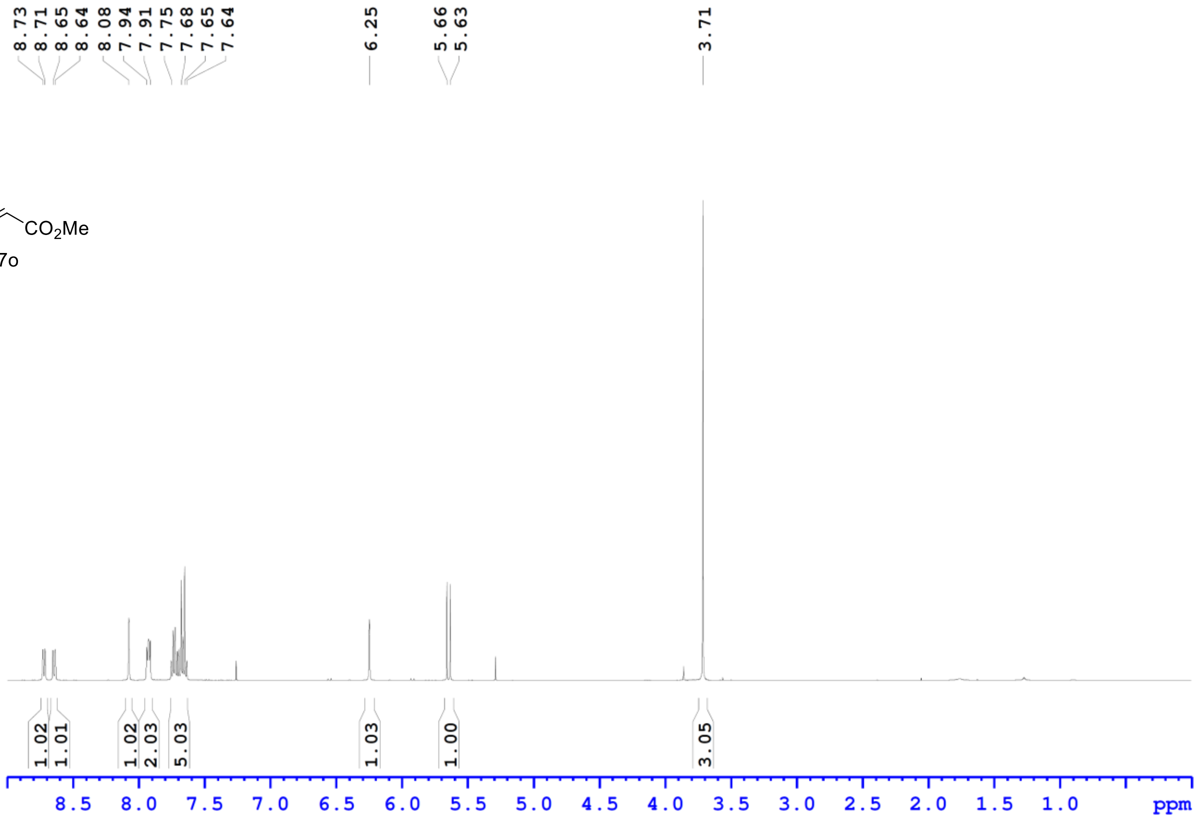
8.73
8.71
8.65
8.64
8.08
7.94
7.91
7.75
7.68
7.65
7.64
6.25
5.66
5.63
3.71
1.02
1.01
1.02
2.03
5.03
1.03
1.00
3.05
8.5 8.0 7.5 7.0 6.5 6.0 5.5 5.0 4.5 4.0 3.5 3.0 2.5 2.0 1.5 1.0 ppm

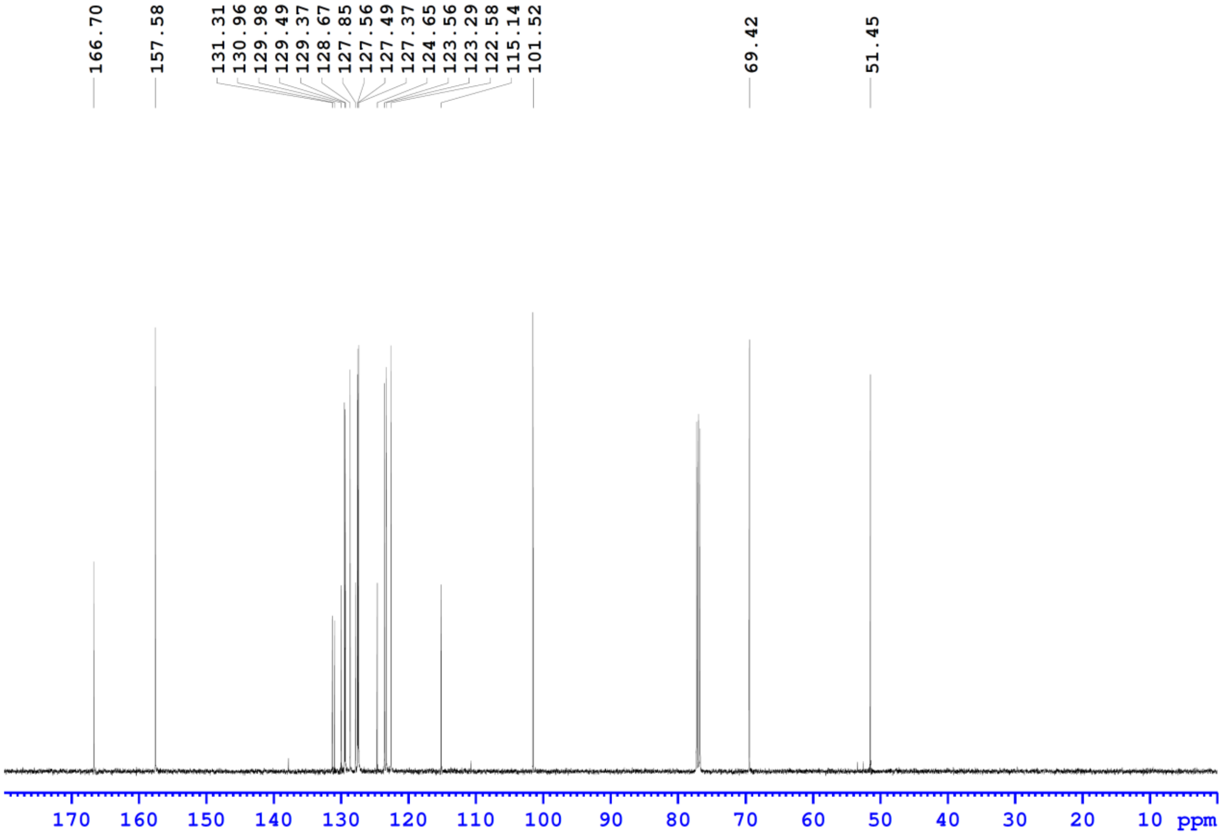
166.70
157.58
131.31
130.96
129.98
129.49
129.37
128.67
127.85
127.56
127.49
127.37
124.65
123.56
123.29
122.58
115.14
101.52
69.42
51.45
170 160 150 140 130 120 110 100 90 80 70 60 50 40 30 20 10 ppm

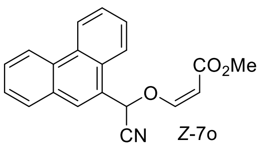
CO2Me
O
CN

Z-7o

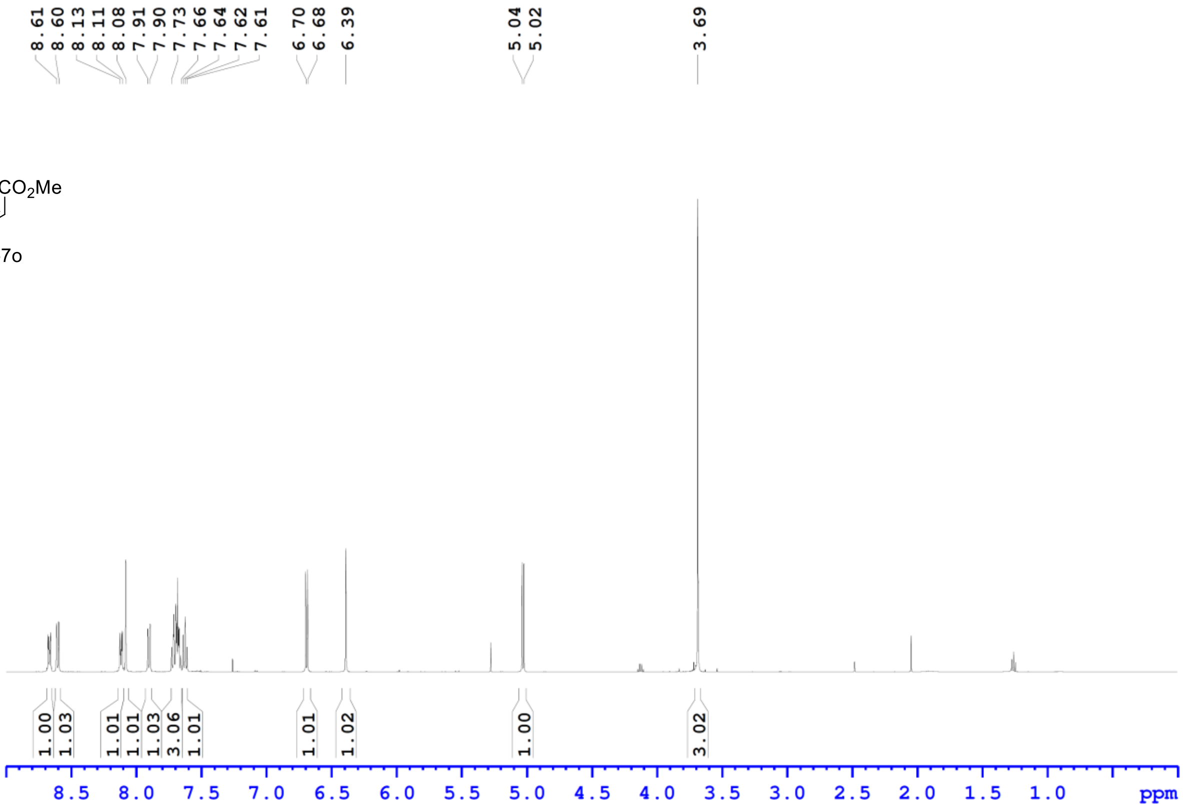
8.61 8.60 8.13 8.11 8.08 7.91 7.90 7.73 7.66 7.64 7.62 7.61 6.70 6.68 6.39 5.04 5.02 3.69
1.00 1.03 1.01 1.01 1.03 3.06 1.01 1.01 1.02 1.00 3.02
8.5 8.0 7.5 7.0 6.5 6.0 5.5 5.0 4.5 4.0 3.5 3.0 2.5 2.0 1.5 1.0 ppm

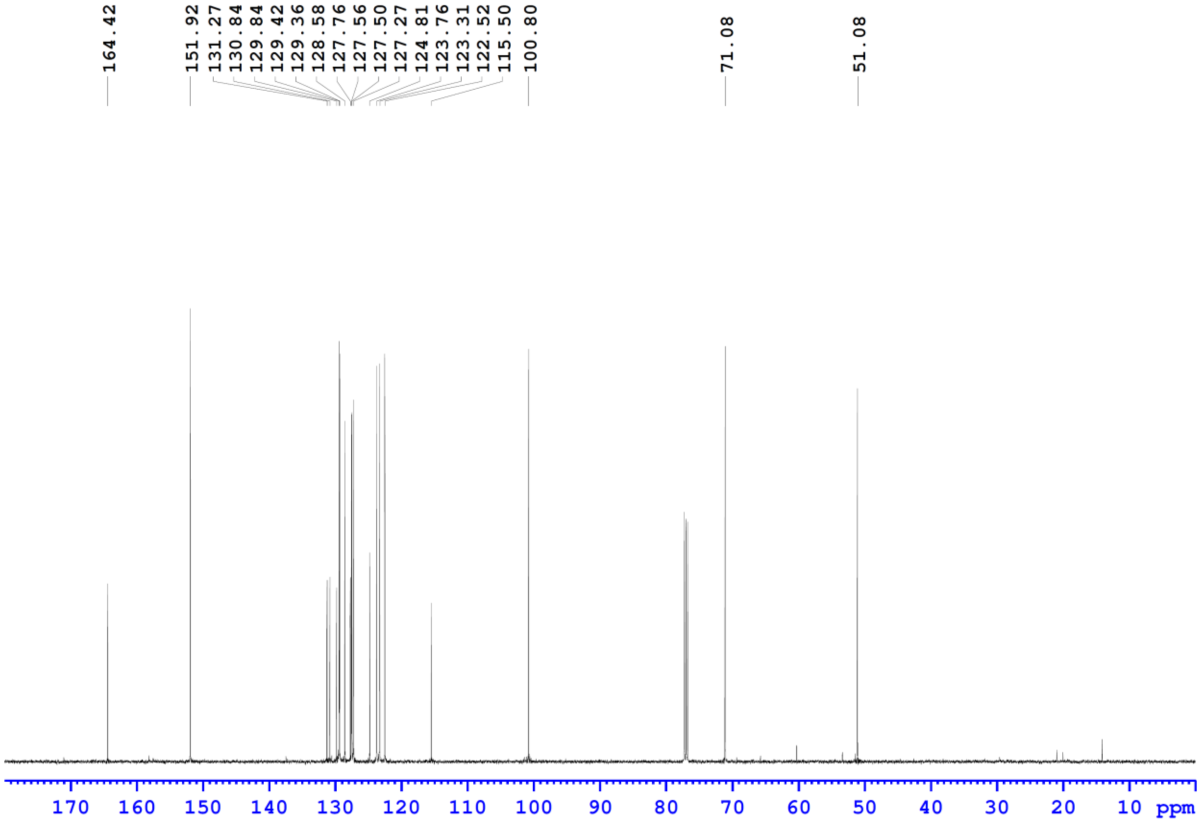
164.42 151.92 131.27 130.84 129.84 129.42 129.36 128.58 127.76 127.56 127.50 127.27 124.81 123.76 123.31 122.52 115.50 100.80 71.08 51.08
170 160 150 140 130 120 110 100 90 80 70 60 50 40 30 20 10 ppm

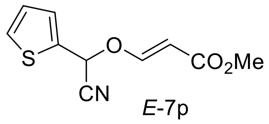
S
O
CO2Me
CN

E-7p

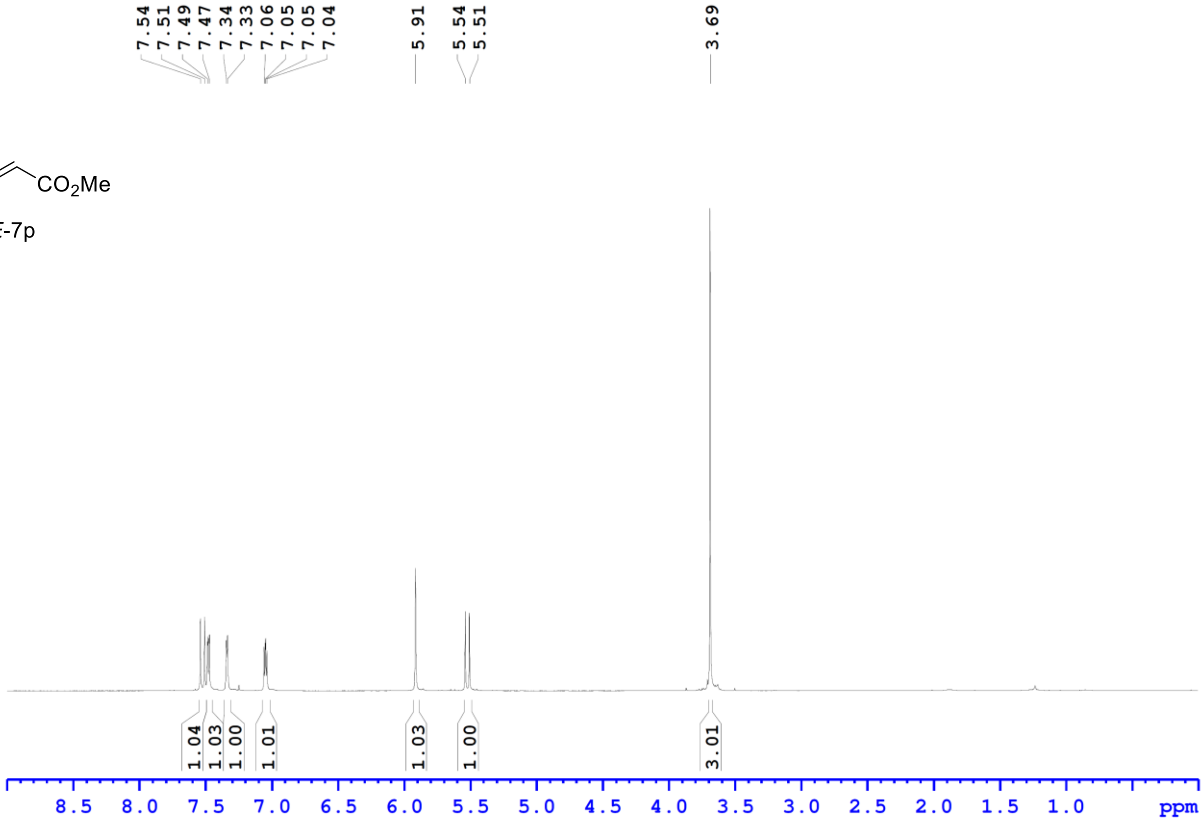
7.54
7.51
7.49
7.47
7.34
7.33
7.06
7.05
7.05
7.04
5.91
5.54
5.51
3.69
1.04
1.03
1.00
1.01
1.03
1.00
3.01
8.5 8.0 7.5 7.0 6.5 6.0 5.5 5.0 4.5 4.0 3.5 3.0 2.5 2.0 1.5 1.0 ppm

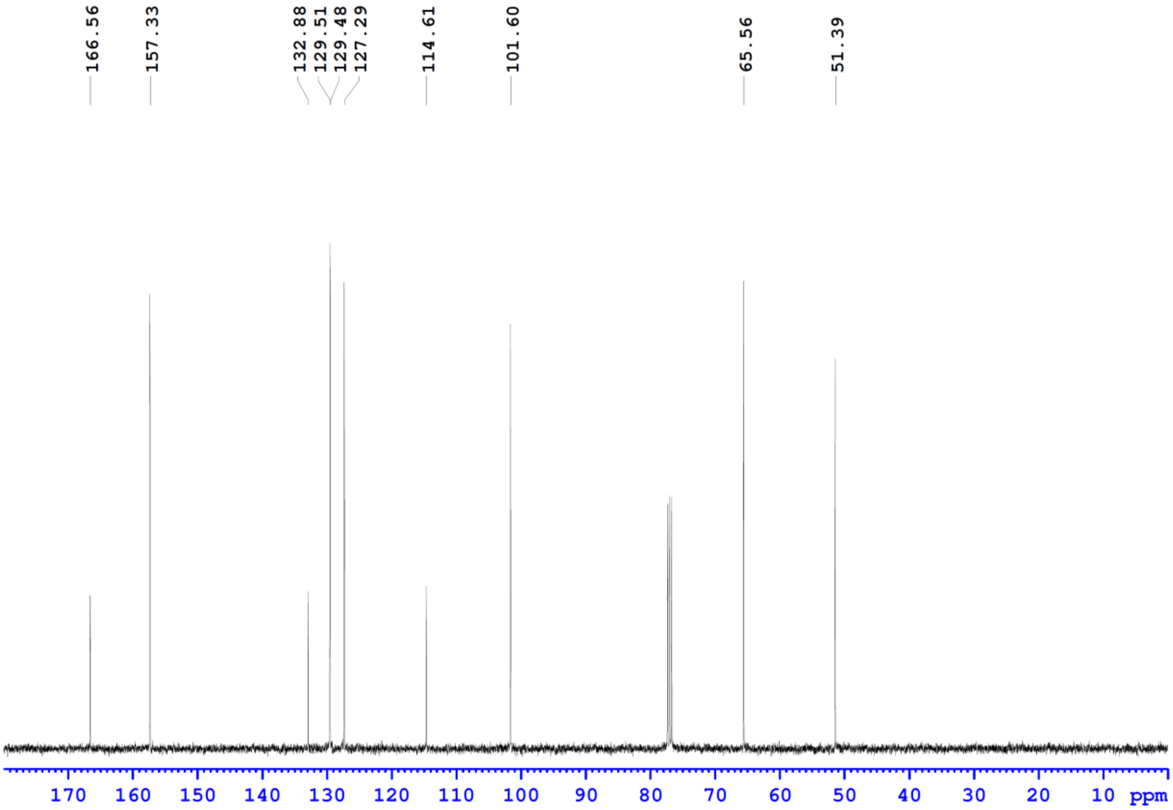
166.56
157.33
132.88
129.51
129.48
127.29
114.61
101.60
65.56
51.39
170 160 150 140 130 120 110 100 90 80 70 60 50 40 30 20 10 ppm

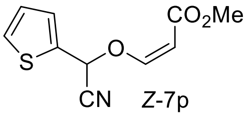
CO2Me
O
S
CN

Z-7p

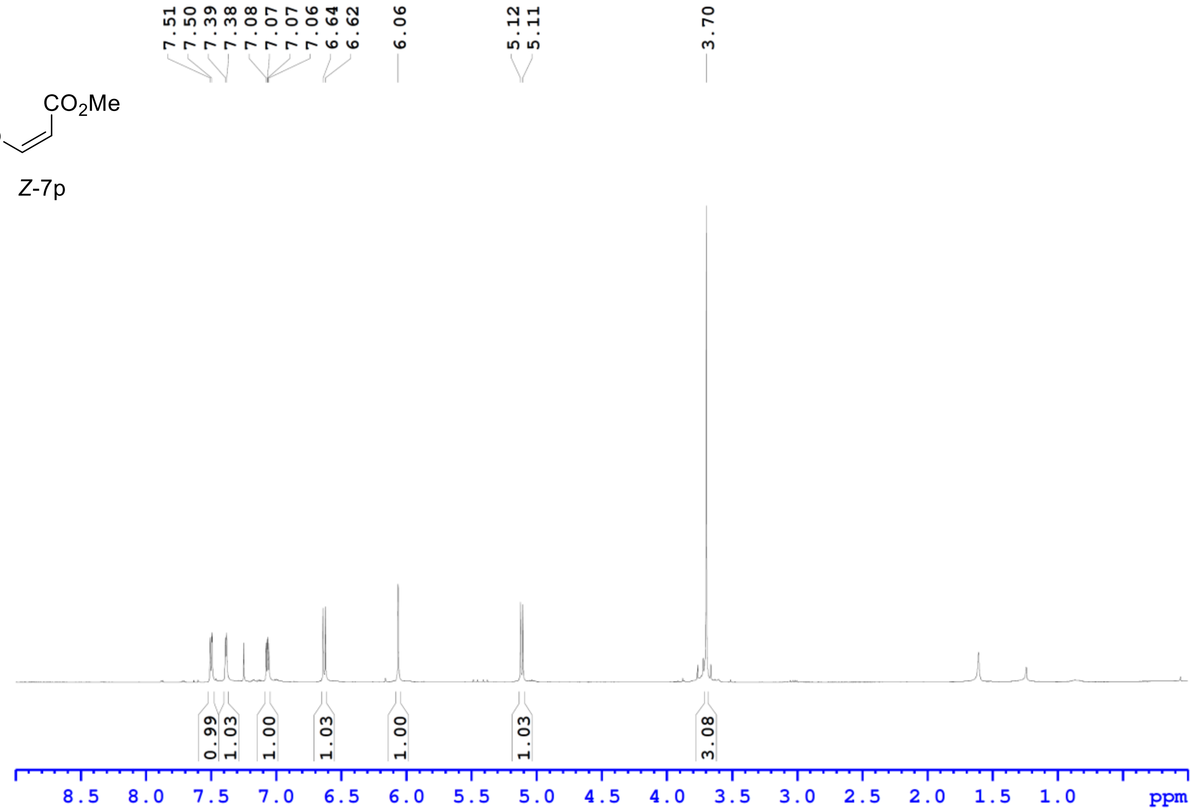
7.51
7.50
7.39
7.38
7.08
7.07
7.07
7.06
6.64
6.62
6.06
5.12
5.11
3.70
0.99
1.03
1.00
1.03
1.00
1.03
3.08
8.5 8.0 7.5 7.0 6.5 6.0 5.5 5.0 4.5 4.0 3.5 3.0 2.5 2.0 1.5 1.0 ppm

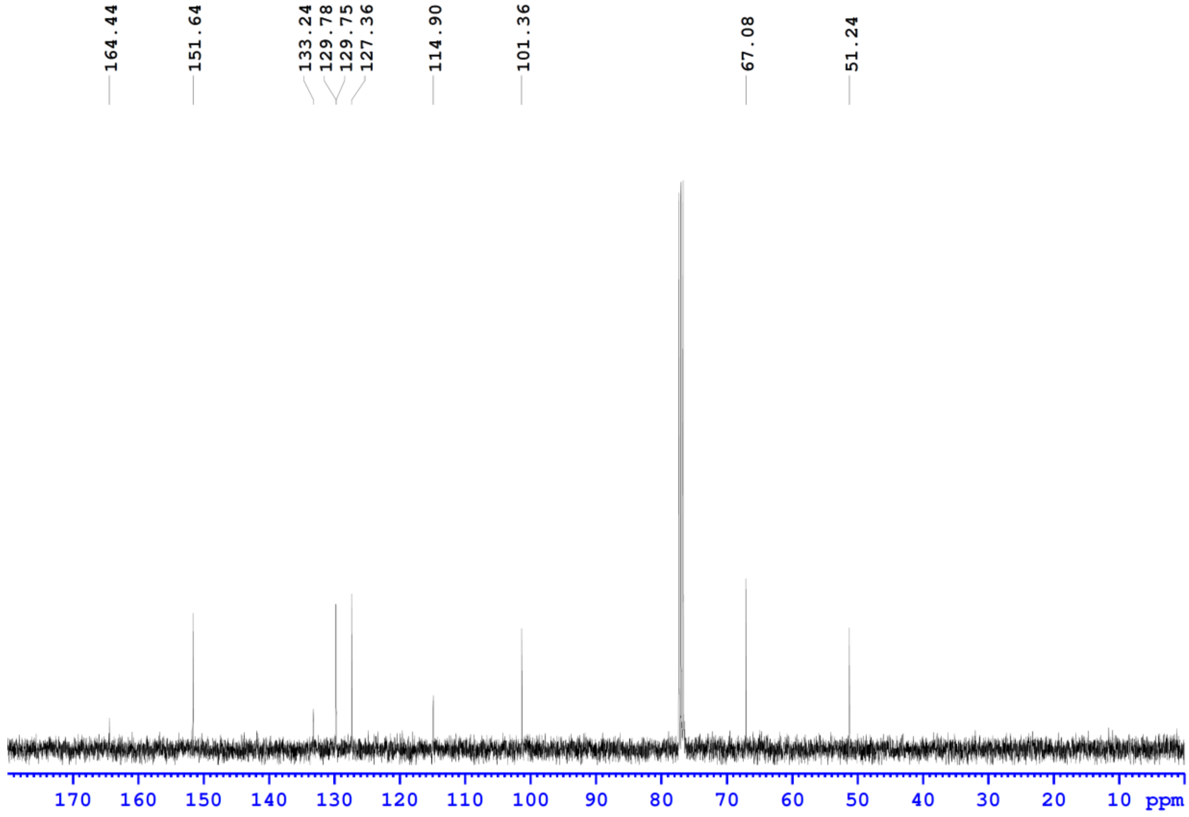
164.44
151.64
133.24
129.78
129.75
127.36
114.90
101.36
67.08
51.24
170 160 150 140 130 120 110 100 90 80 70 60 50 40 30 20 10 ppm

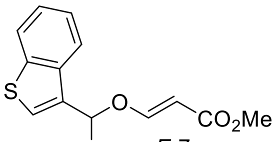
S
O
CO2Me
CN

E-7q

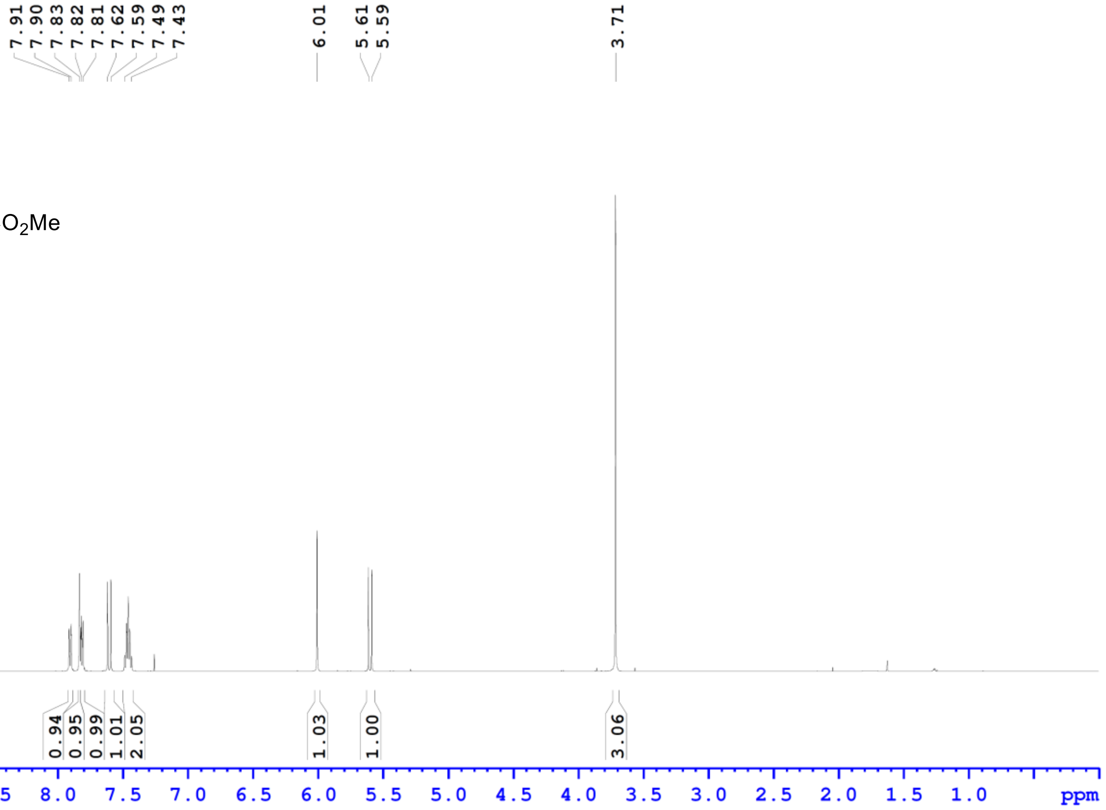
7.91
7.90
7.83
7.82
7.81
7.62
7.59
7.49
7.43
6.01
5.61
5.59
3.71
0.94
0.95
0.99
1.01
2.05
1.03
1.00
3.06
8.5 8.0 7.5 7.0 6.5 6.0 5.5 5.0 4.5 4.0 3.5 3.0 2.5 2.0 1.5 1.0 ppm

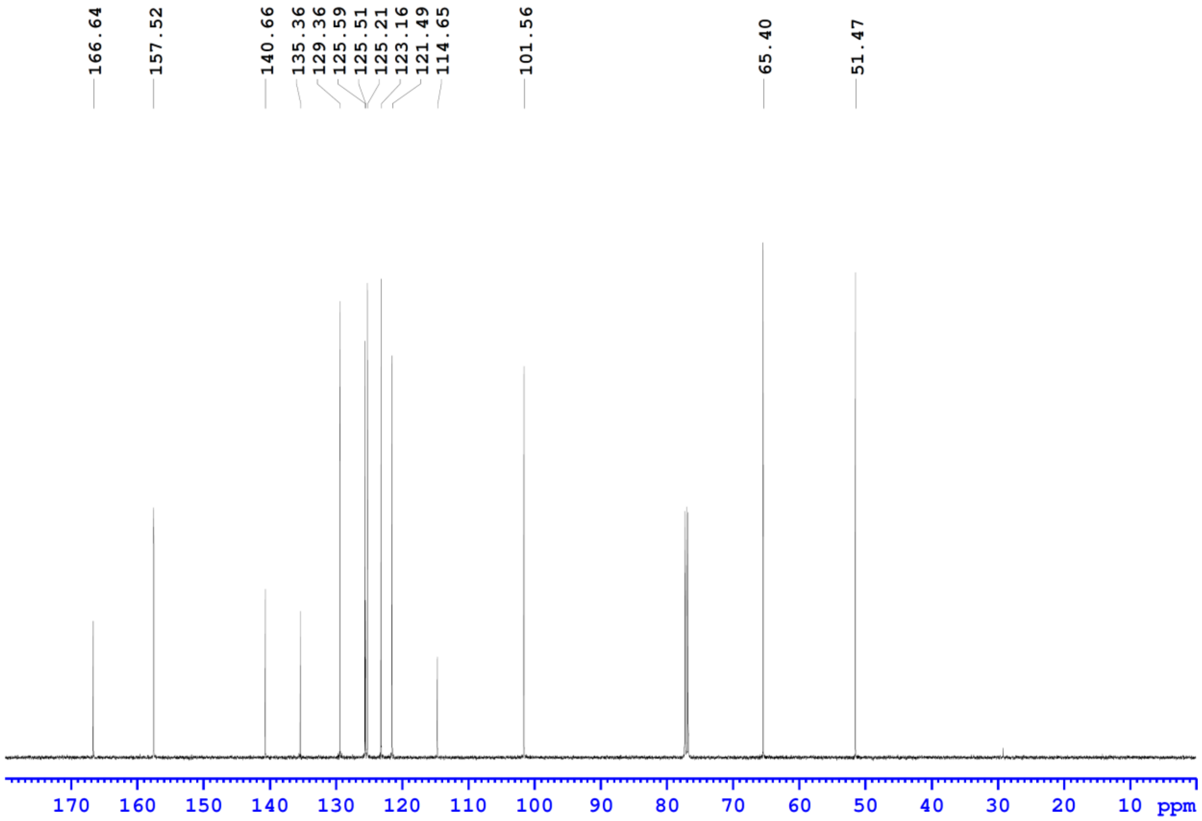
166.64
157.52
140.66
135.36
129.36
125.59
125.51
125.21
123.16
121.49
114.65
101.56
65.40
51.47
170 160 150 140 130 120 110 100 90 80 70 60 50 40 30 20 10 ppm

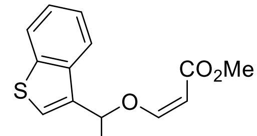
CO2Me
S
O
CN

Z-7q

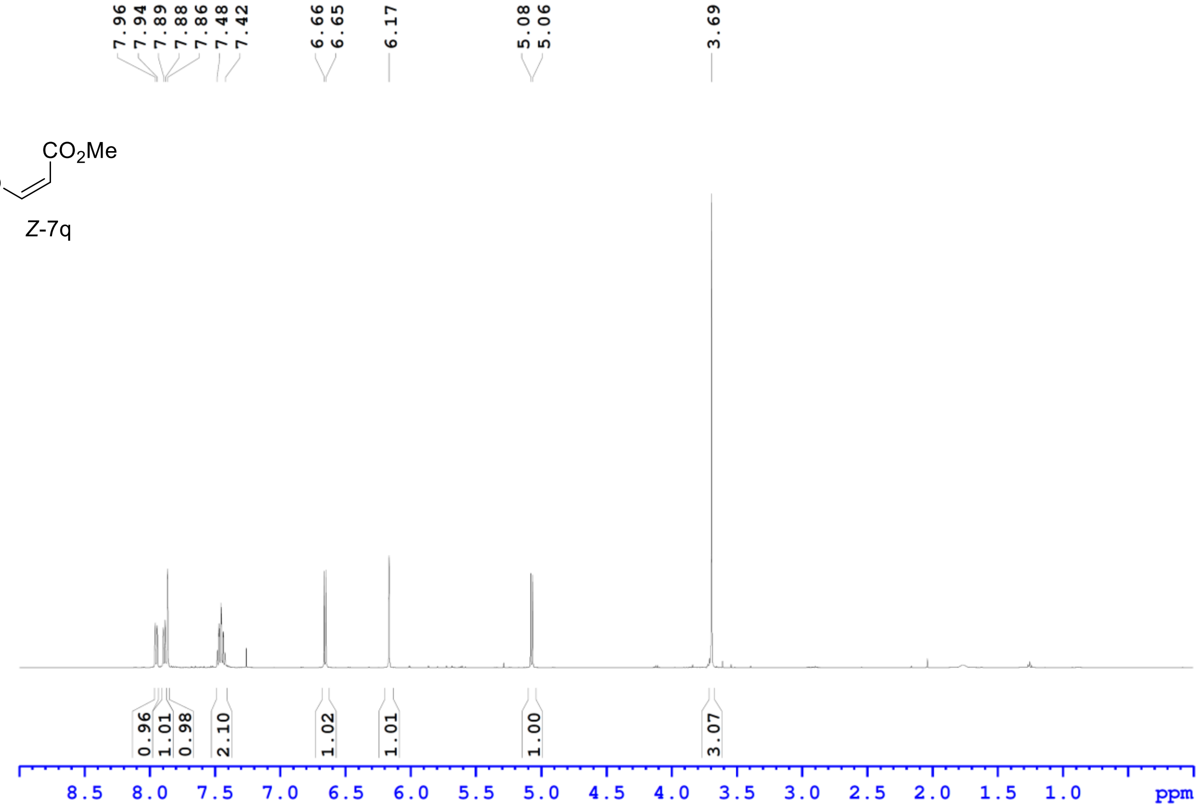
7.96
7.94
7.89
7.88
7.86
7.48
7.42
6.66
6.65
6.17
5.08
5.06
3.69
0.96
1.01
0.98
2.10
1.02
1.01
1.00
3.07
8.5 8.0 7.5 7.0 6.5 6.0 5.5 5.0 4.5 4.0 3.5 3.0 2.5 2.0 1.5 1.0 ppm

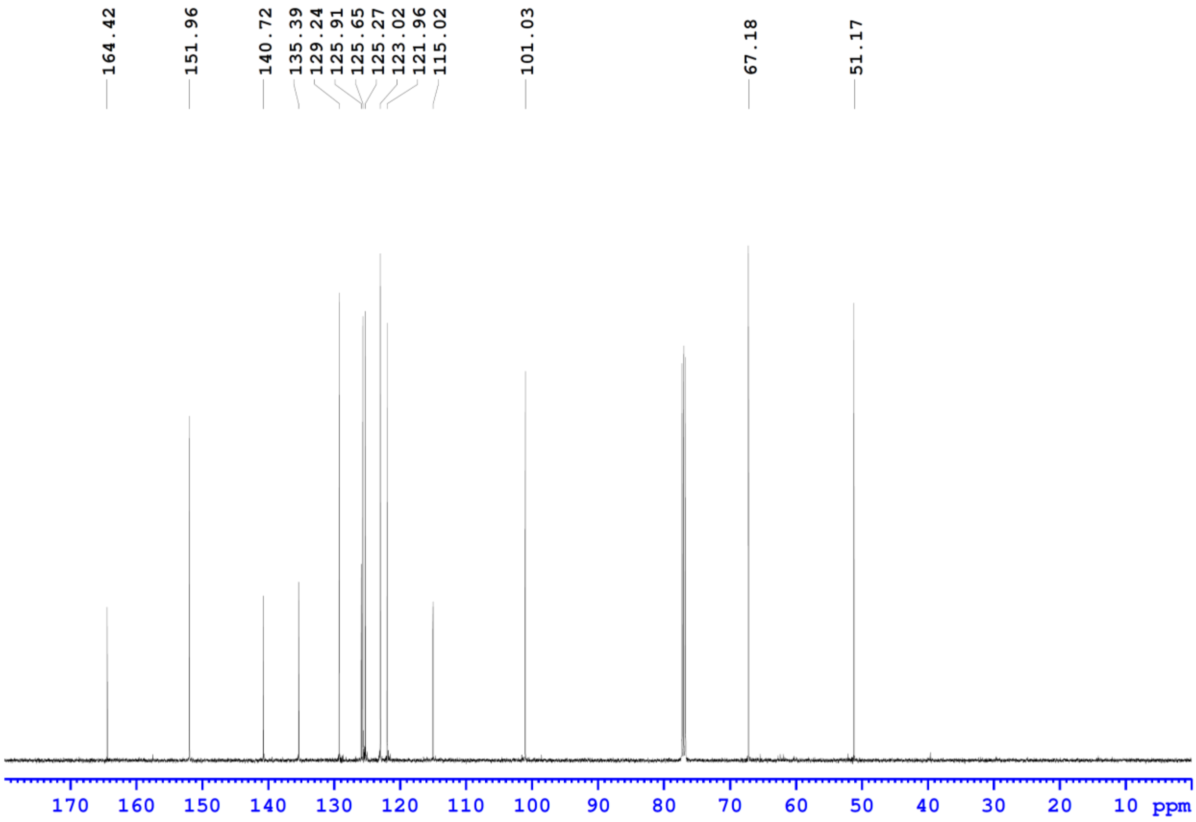
164.42
151.96
140.72
135.39
129.24
125.91
125.65
125.27
123.02
121.96
115.02
101.03
67.18
51.17
170 160 150 140 130 120 110 100 90 80 70 60 50 40 30 20 10 ppm

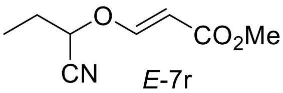

O
CO2Me
CN

E-7r

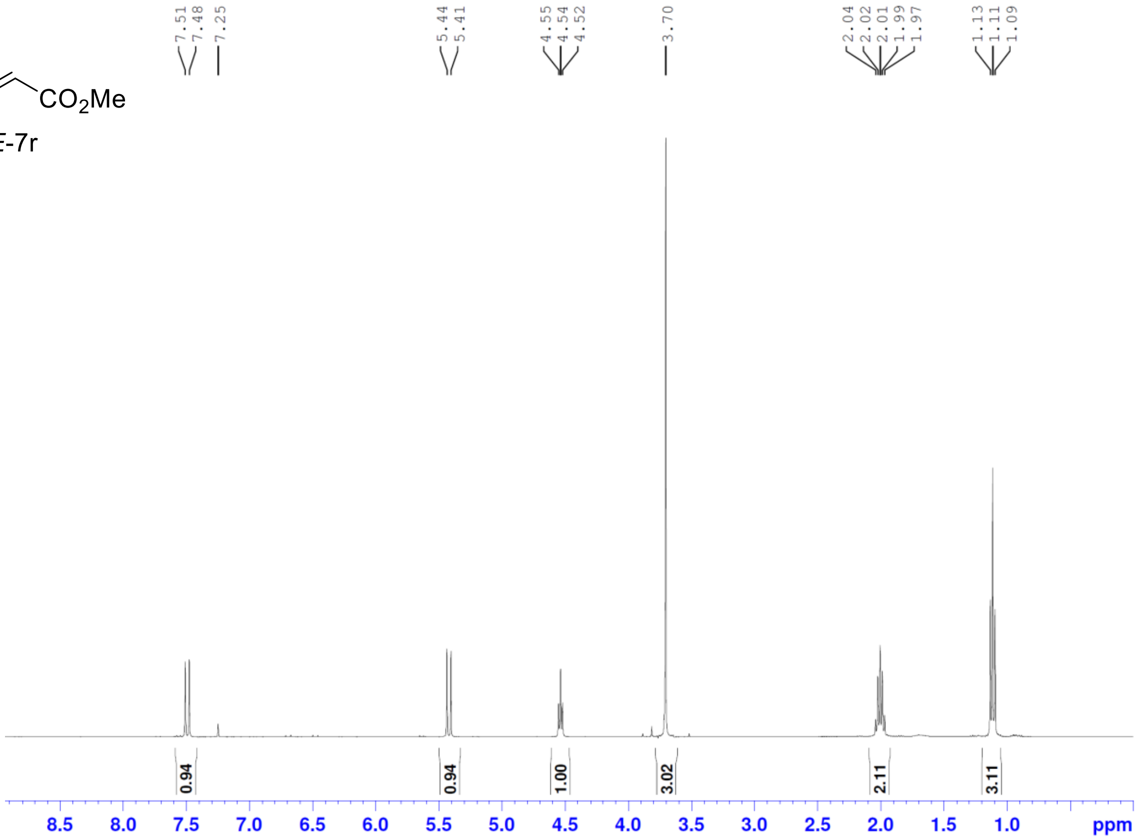

7.51
7.48
7.25
5.44
5.41
4.55
4.54
4.52
3.70
2.04
2.02
2.01
1.99
1.97
1.13
1.11
1.09
0.94
0.94
1.00
3.02
2.11
3.11
8.5 8.0 7.5 7.0 6.5 6.0 5.5 5.0 4.5 4.0 3.5 3.0 2.5 2.0 1.5 1.0 ppm

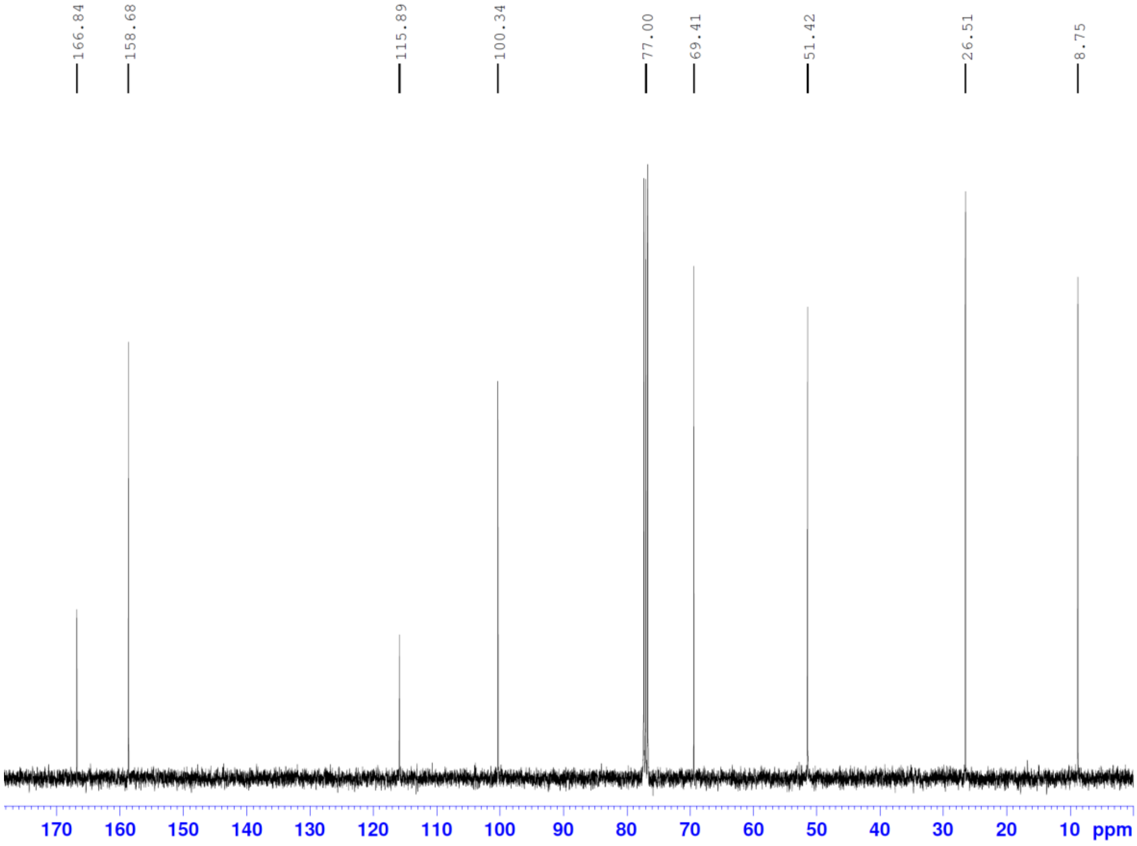

166.84
158.68
115.89
100.34
77.00
69.41
51.42
26.51
8.75
170 160 150 140 130 120 110 100 90 80 70 60 50 40 30 20 10 ppm

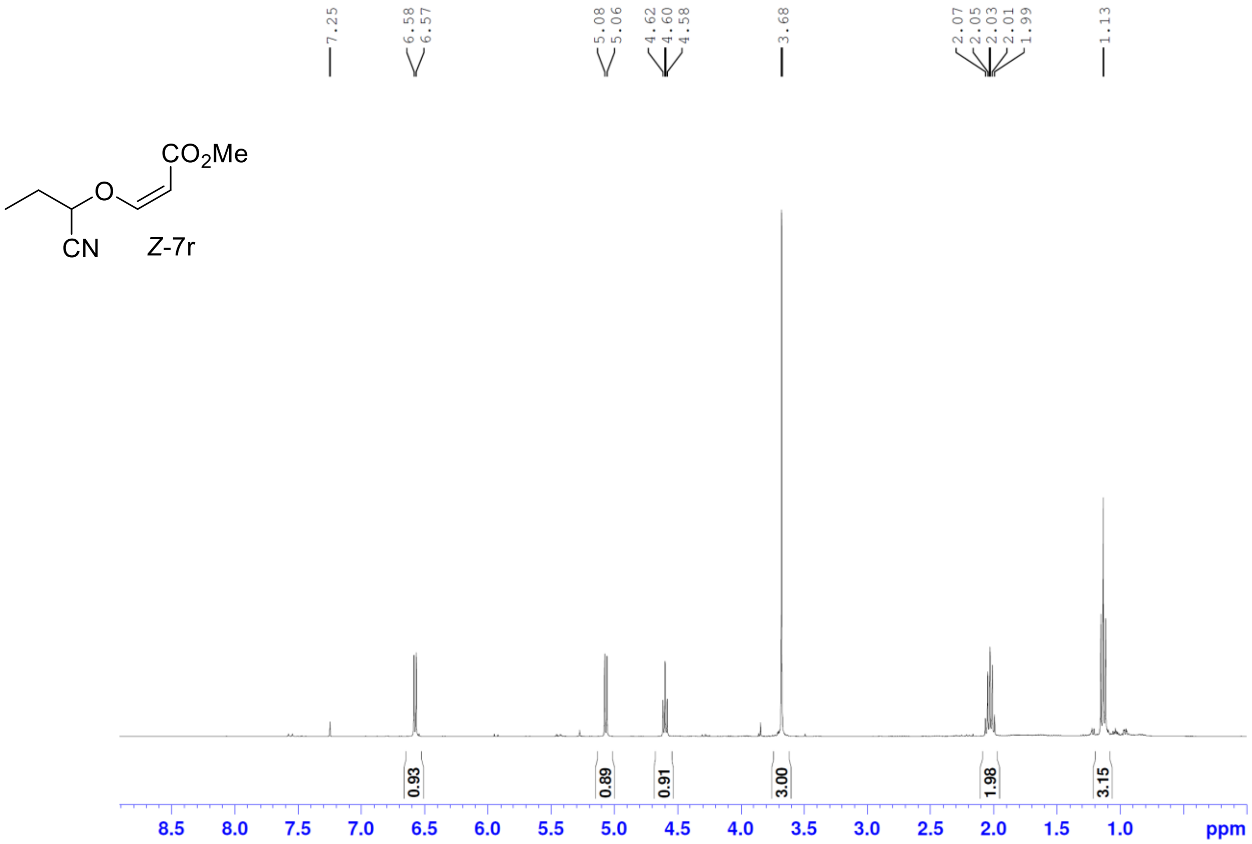

Z-7r
7.25
6.58
6.57
5.08
5.06
4.62
4.60
4.58
3.68
2.07
2.05
2.03
2.01
1.99
1.13
0.93
0.89
0.91
3.00
1.98
3.15
8.5 8.0 7.5 7.0 6.5 6.0 5.5 5.0 4.5 4.0 3.5 3.0 2.5 2.0 1.5 1.0 ppm

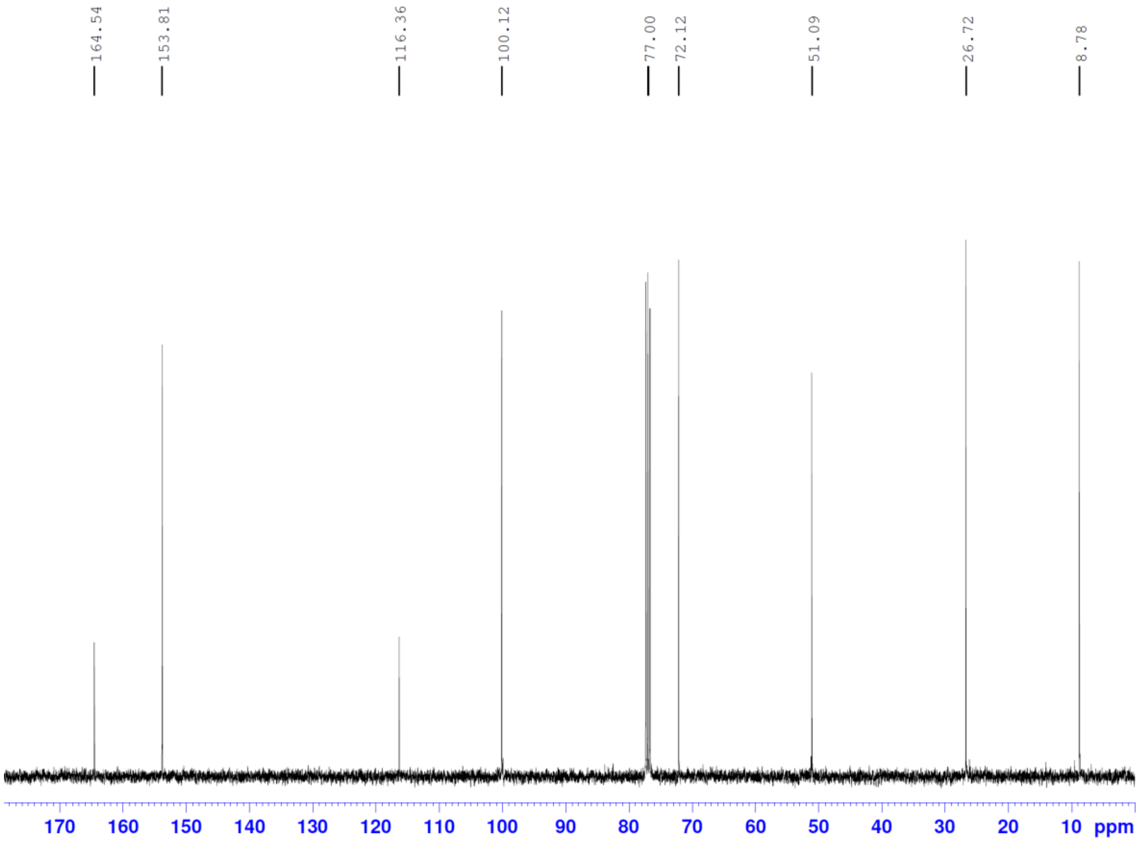
164.54
153.81
116.36
100.12
77.00
72.12
51.09
26.72
8.78
170 160 150 140 130 120 110 100 90 80 70 60 50 40 30 20 10 ppm

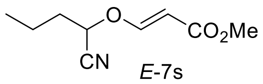
CO2Me
CN

E-7s

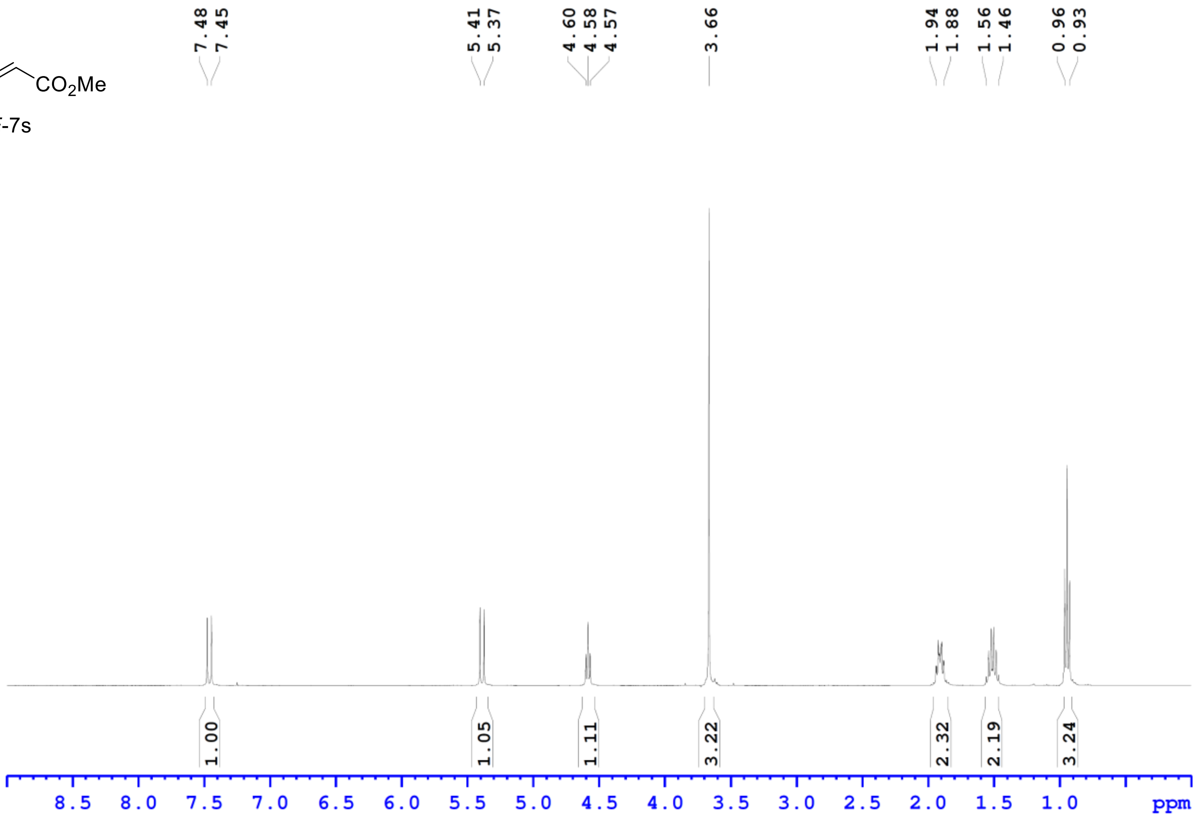
7.48
7.45
5.41
5.37
4.60
4.58
4.57
3.66
1.94
1.88
1.56
1.46
0.96
0.93
1.00
1.05
1.11
3.22
2.32
2.19
3.24
ppm

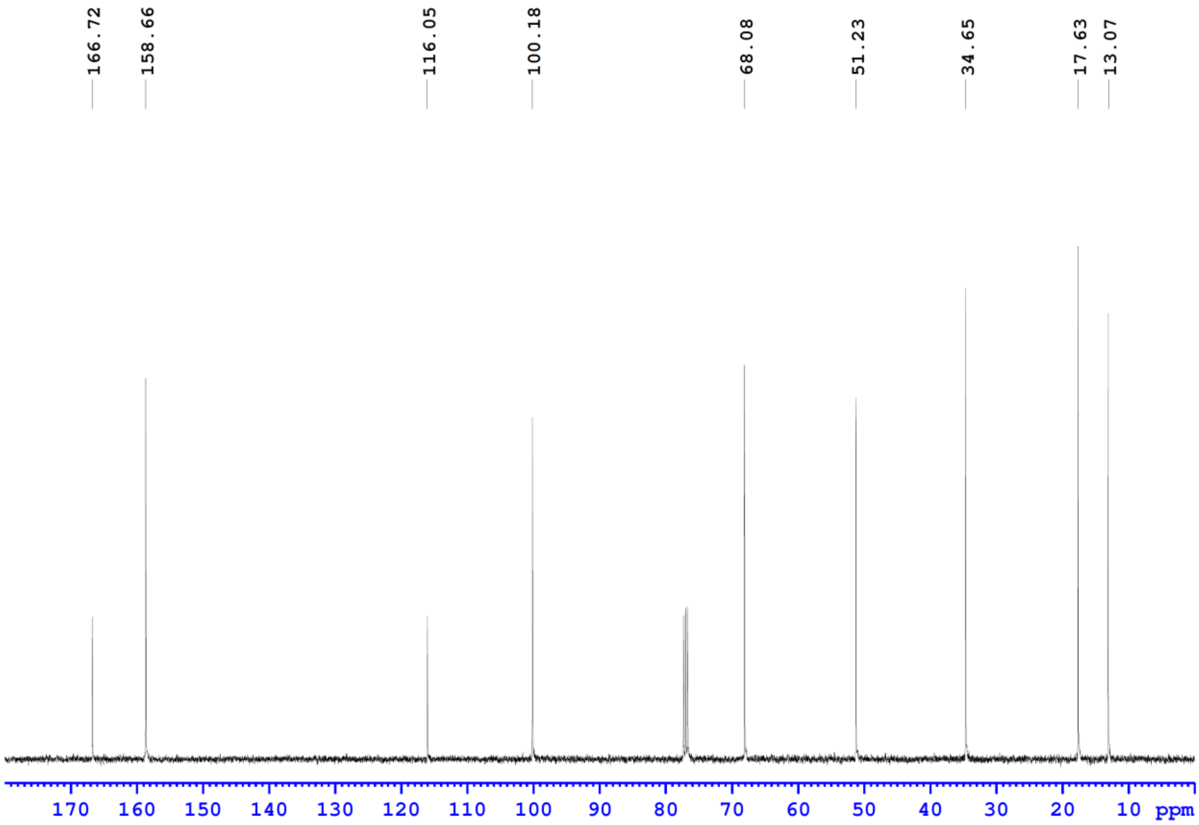
166.72
158.66
116.05
100.18
68.08
51.23
34.65
17.63
13.07
ppm

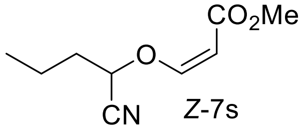
CO2Me
O
CN

Z-7s

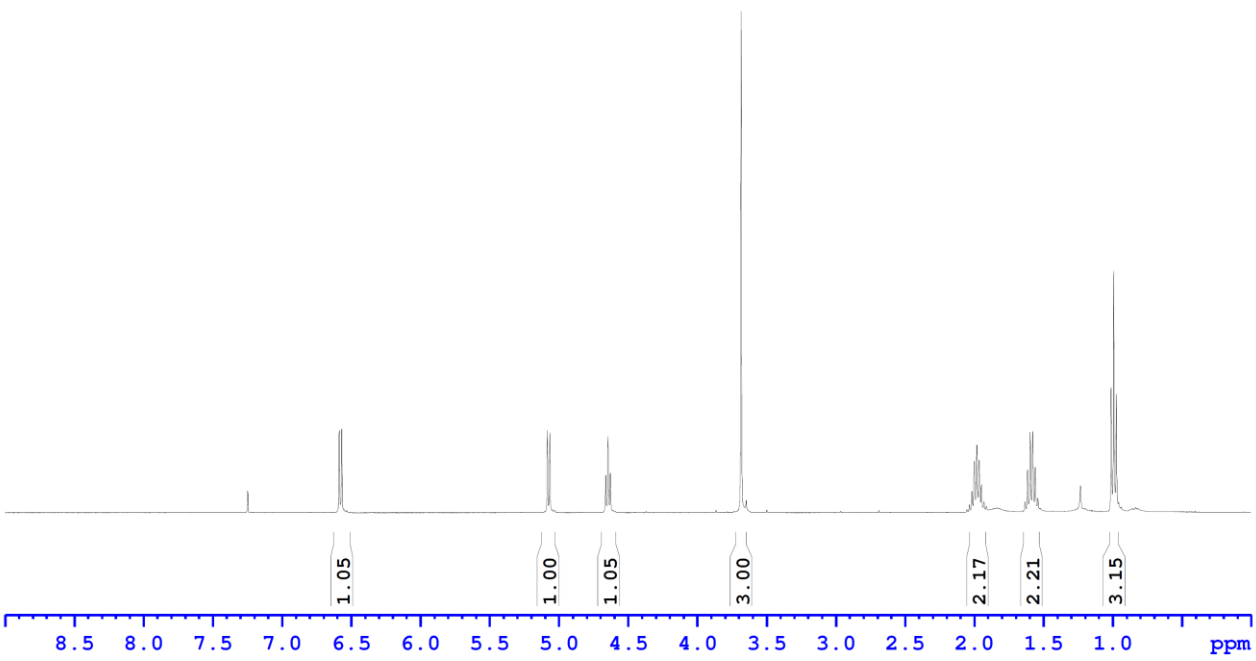
6.59
6.57
5.08
5.07
4.66
4.63
3.69
2.03
1.93
1.63
1.54
1.01
0.97
1.05
1.00
1.05
3.00
2.17
2.21
3.15
8.5 8.0 7.5 7.0 6.5 6.0 5.5 5.0 4.5 4.0 3.5 3.0 2.5 2.0 1.5 1.0 ppm

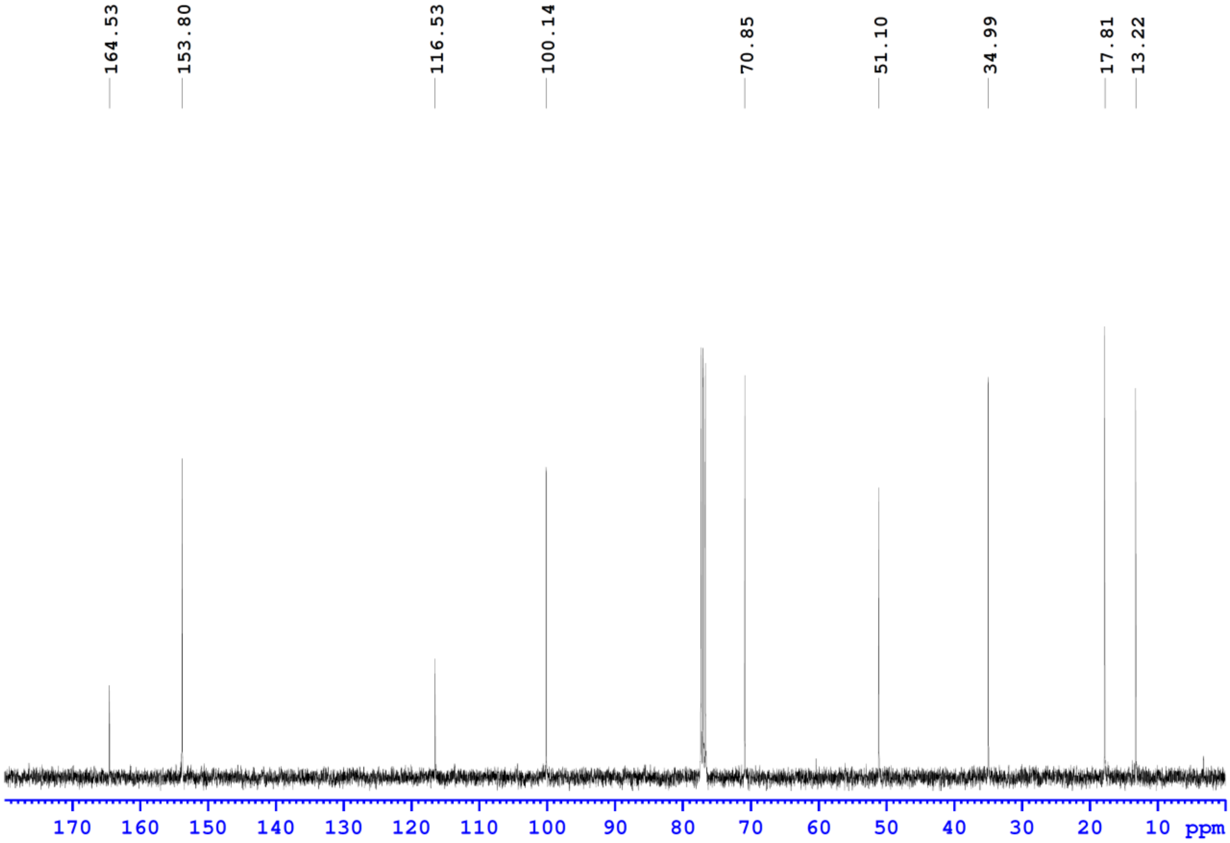
164.53
153.80
116.53
100.14
70.85
51.10
34.99
17.81
13.22
170 160 150 140 130 120 110 100 90 80 70 60 50 40 30 20 10 ppm

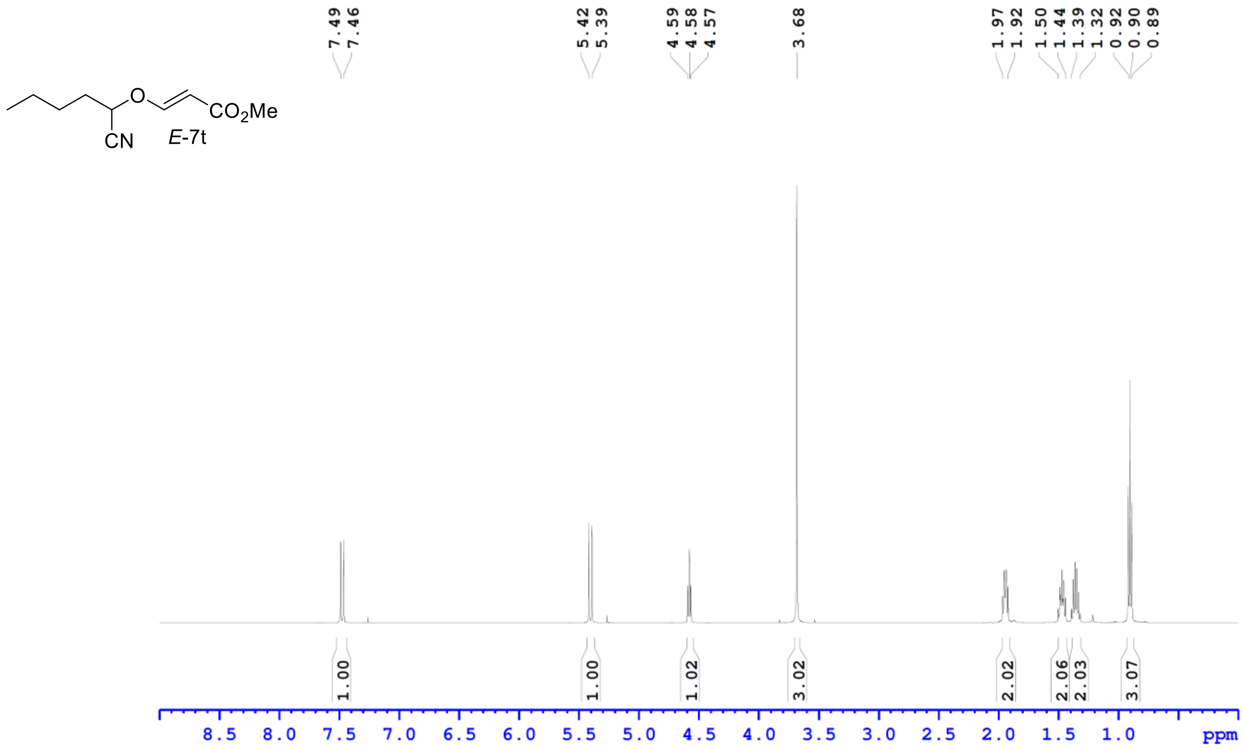

O
CO2Me
CN
E-7t
7.49
7.46
5.42
5.39
4.59
4.58
4.57
3.68
1.97
1.92
1.50
1.44
1.39
1.32
0.92
0.90
0.89
1.00
1.00
1.02
3.02
2.02
2.06
2.03
3.07
8.5 8.0 7.5 7.0 6.5 6.0 5.5 5.0 4.5 4.0 3.5 3.0 2.5 2.0 1.5 1.0 ppm

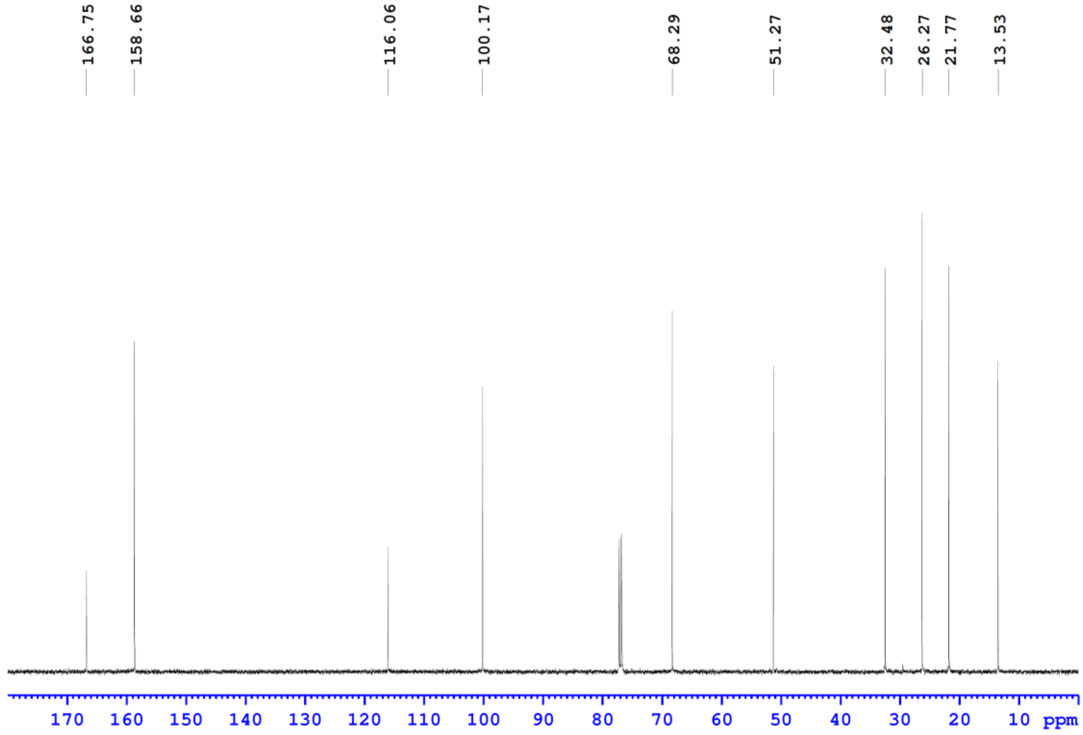

166.75
158.66
116.06
100.17
68.29
51.27
32.48
26.27
21.77
13.53
170 160 150 140 130 120 110 100 90 80 70 60 50 40 30 20 10 ppm

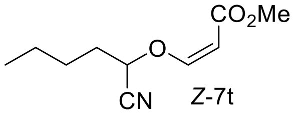
CO2Me
O
CN

Z-7t

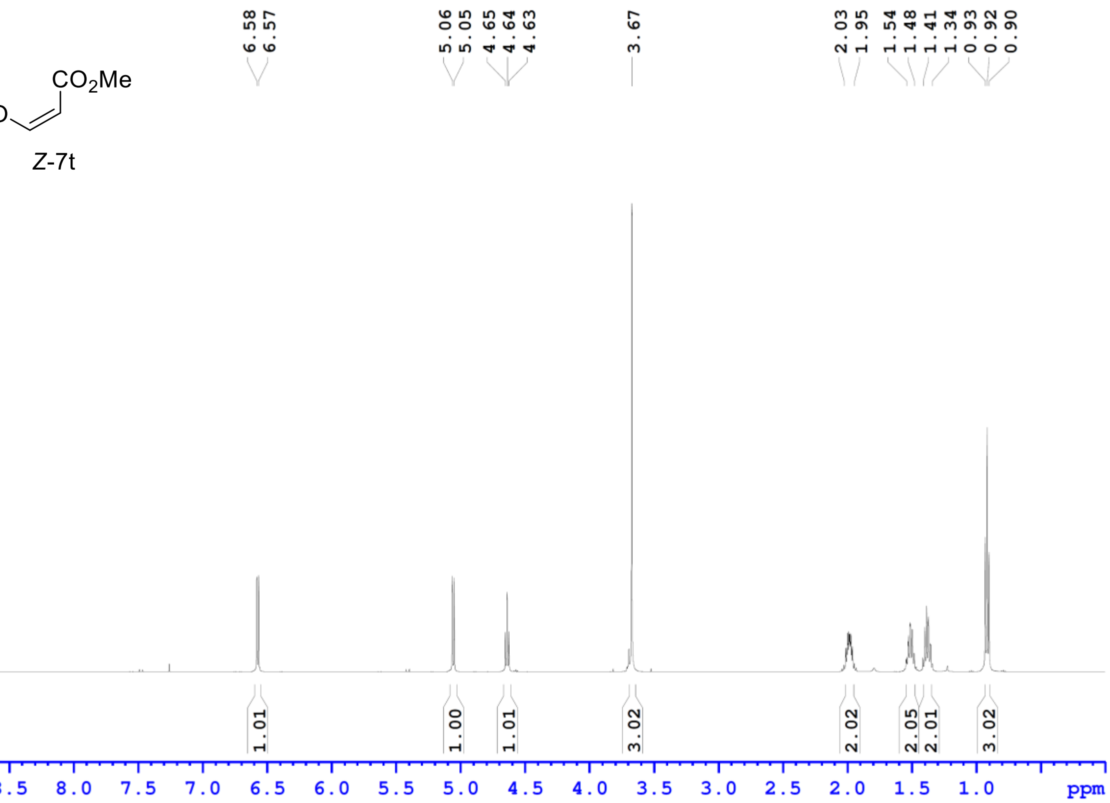
6.58
6.57
5.06
5.05
4.65
4.64
4.63
3.67
2.03
1.95
1.54
1.48
1.41
1.34
0.93
0.92
0.90
1.01
1.00
1.01
3.02
2.02
2.05
2.01
3.02
8.5 8.0 7.5 7.0 6.5 6.0 5.5 5.0 4.5 4.0 3.5 3.0 2.5 2.0 1.5 1.0 ppm

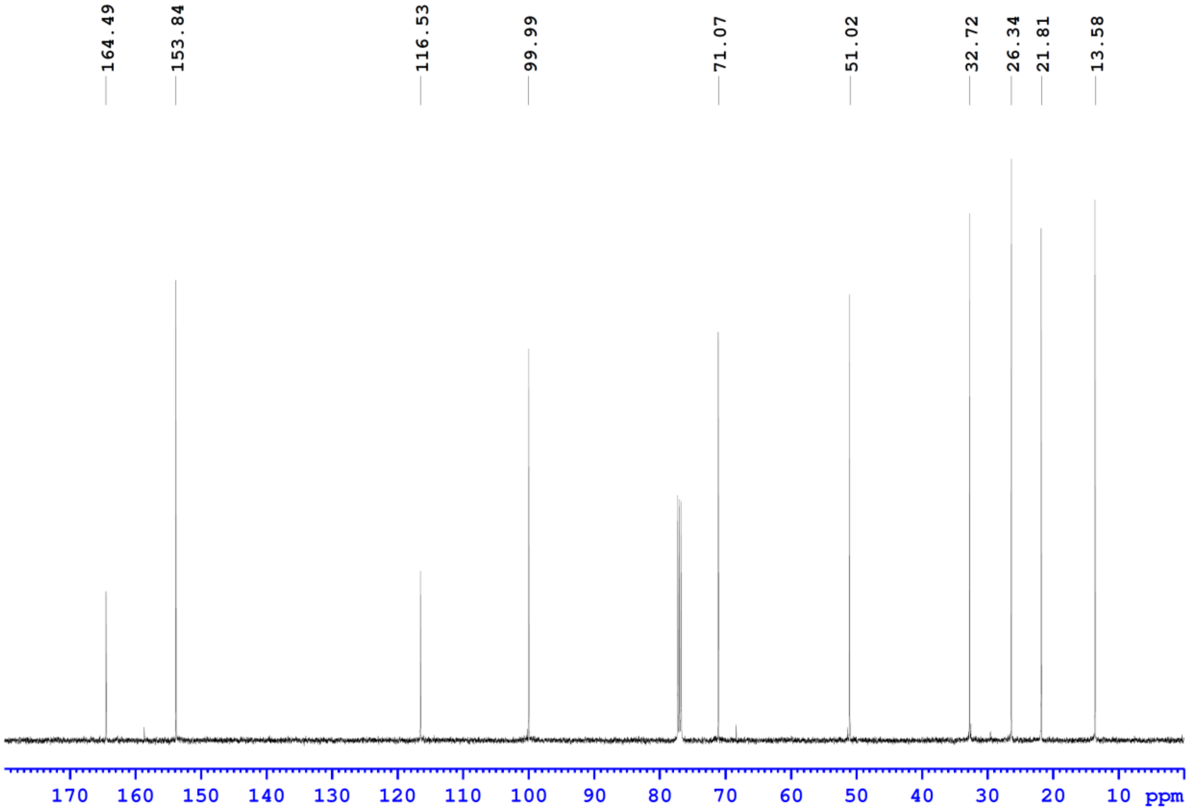
164.49
153.84
116.53
99.99
71.07
51.02
32.72
26.34
21.81
13.58
170 160 150 140 130 120 110 100 90 80 70 60 50 40 30 20 10 ppm

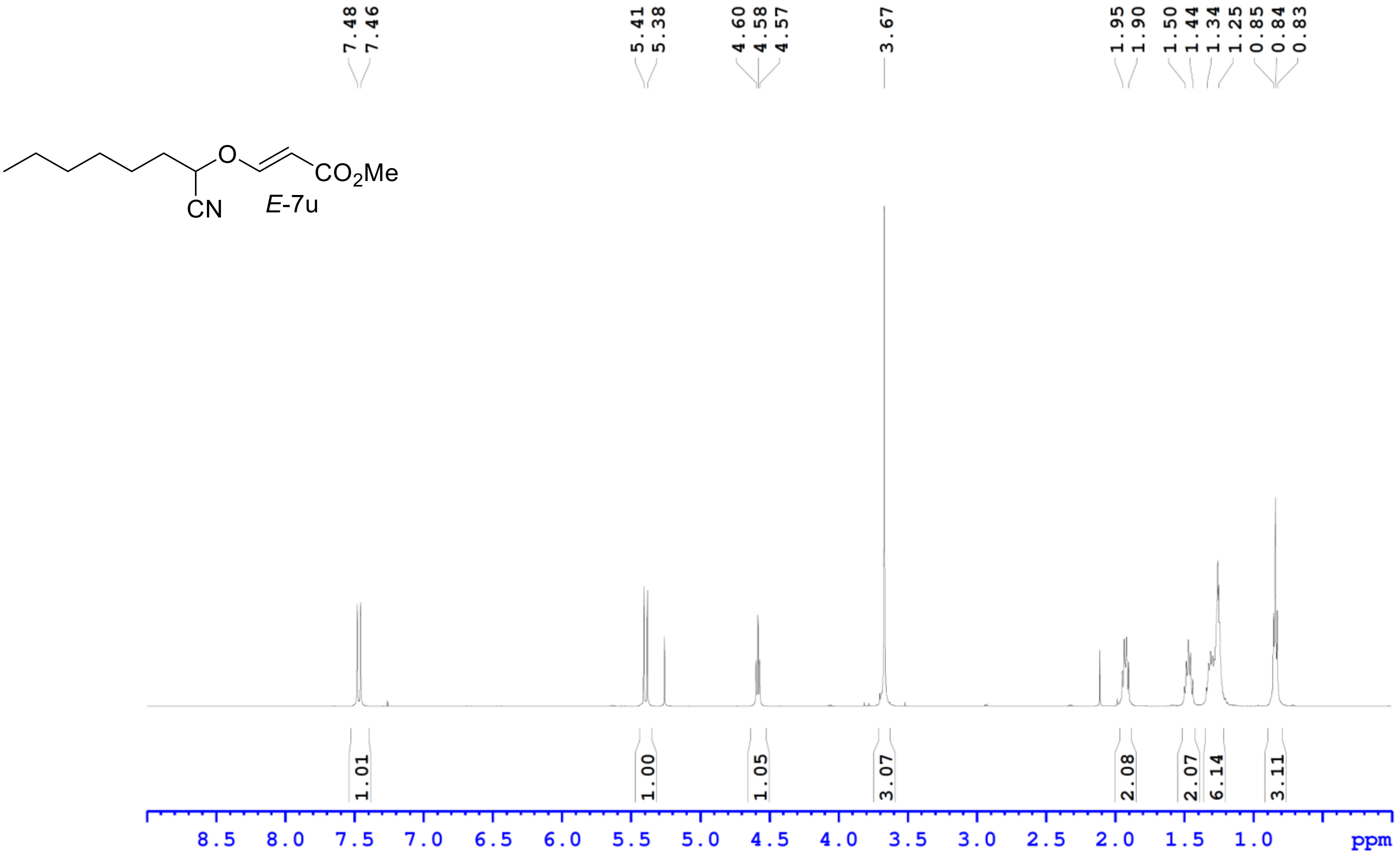

E-7u

7.48
7.46
5.41
5.38
4.60
4.58
4.57
3.67
1.95
1.90
1.50
1.44
1.34
1.25
0.85
0.84
0.83
1.01
1.00
1.05
3.07
2.08
2.07
6.14
3.11
ppm

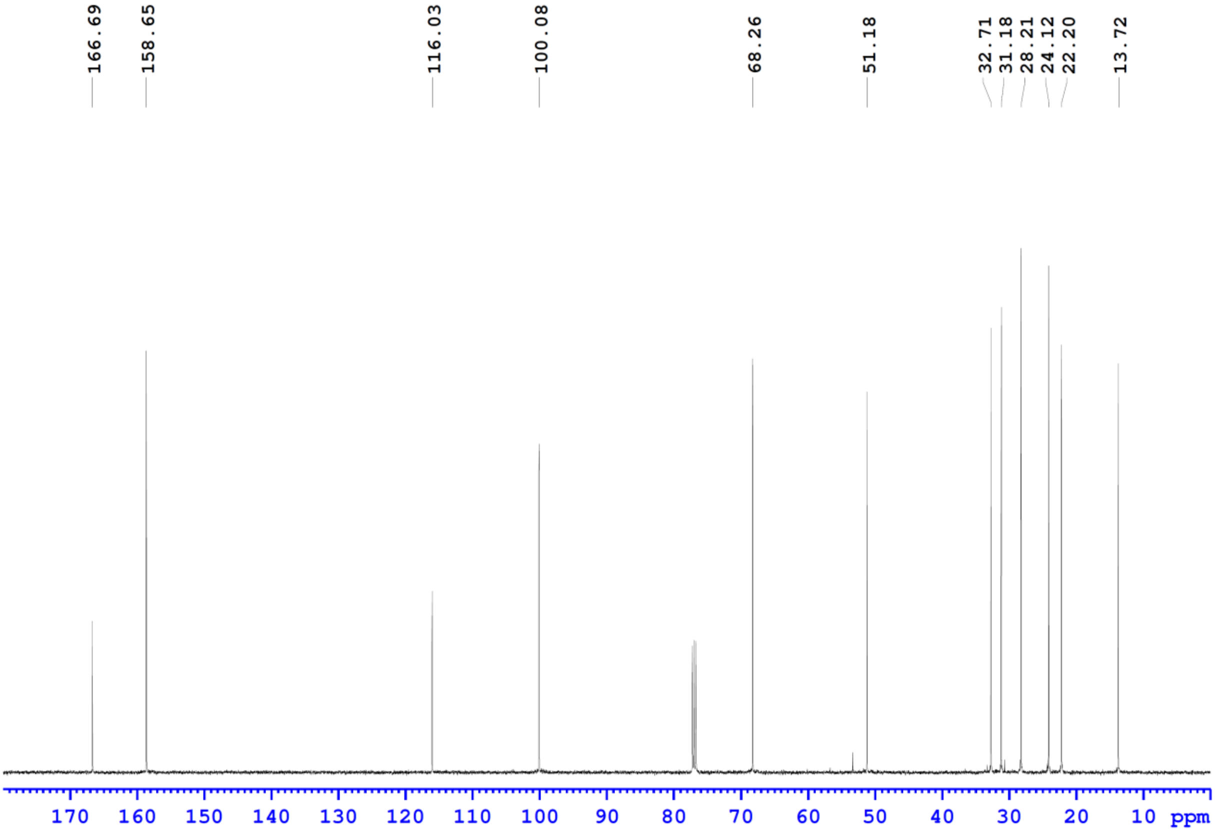
166.69
158.65
116.03
100.08
68.26
51.18
32.71
31.18
28.21
24.12
22.20
13.72
ppm

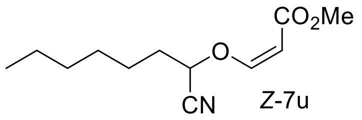

Z-7u

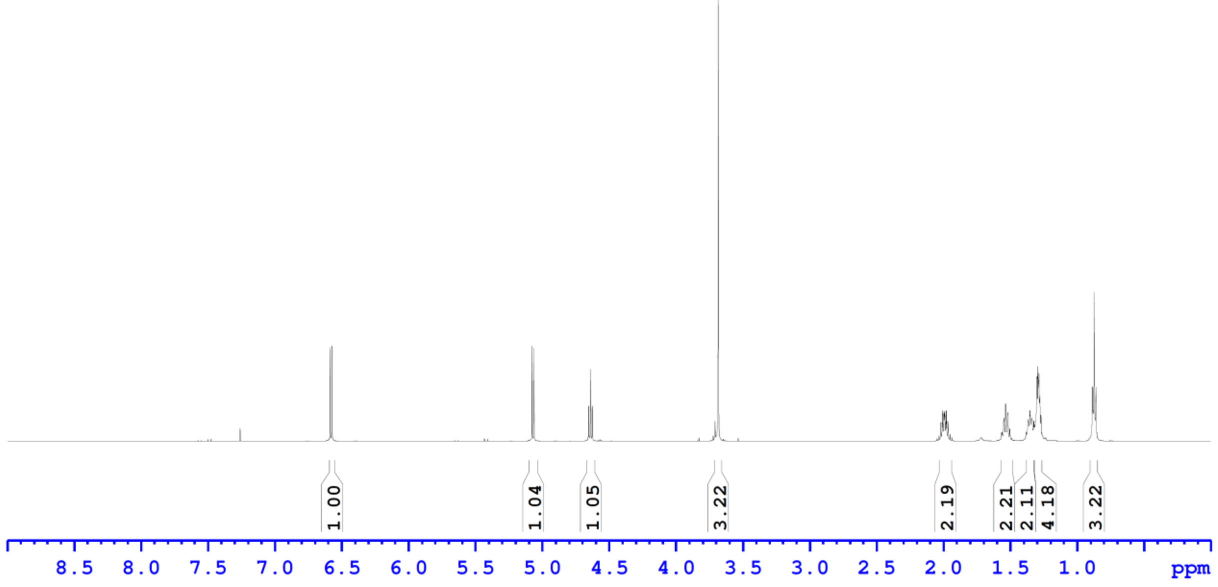
6.59
6.57
5.08
5.06
4.65
4.64
4.63
3.68
2.03
1.95
1.56
1.50
1.38
1.32
1.32
1.27
0.89
0.87
0.86
1.00
1.04
1.05
3.22
2.19
2.21
2.11
4.18
3.22
8.5 8.0 7.5 7.0 6.5 6.0 5.5 5.0 4.5 4.0 3.5 3.0 2.5 2.0 1.5 1.0 ppm

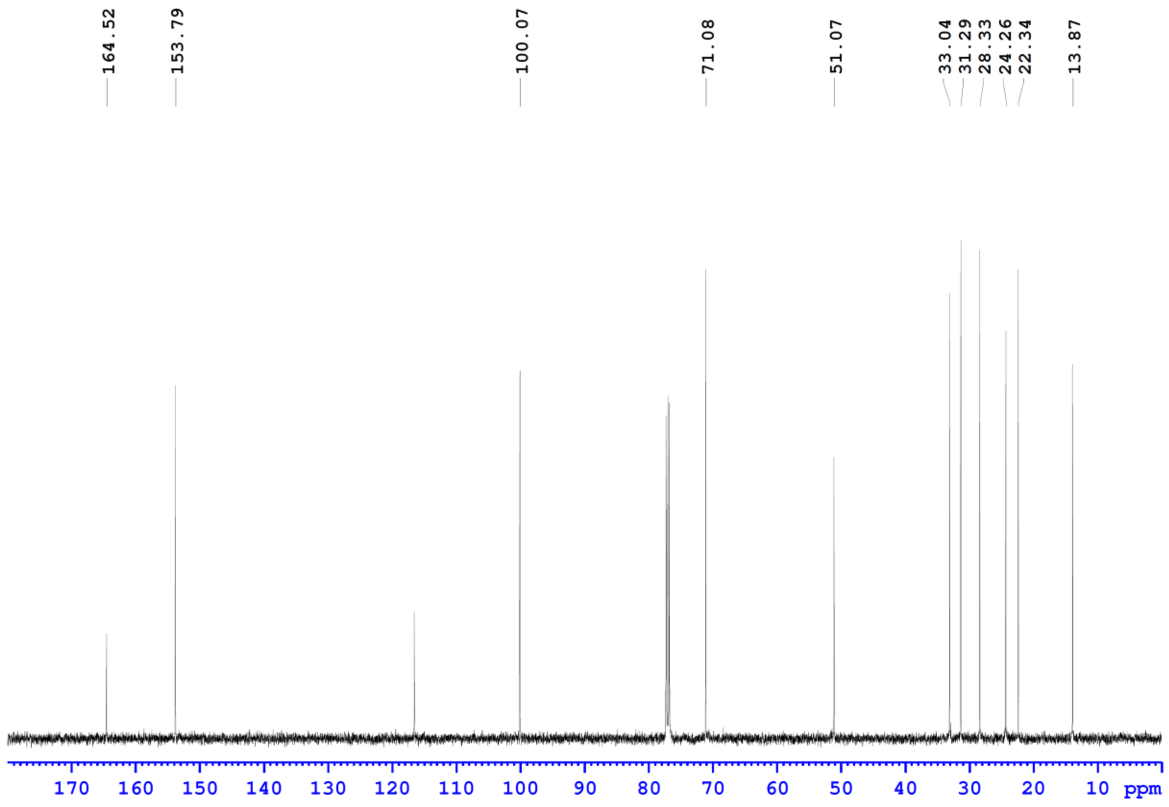
164.52
153.79
100.07
71.08
51.07
33.04
31.29
28.33
24.26
22.34
13.87
170 160 150 140 130 120 110 100 90 80 70 60 50 40 30 20 10 ppm

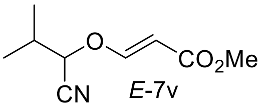
CO2Me
CN

E-7v

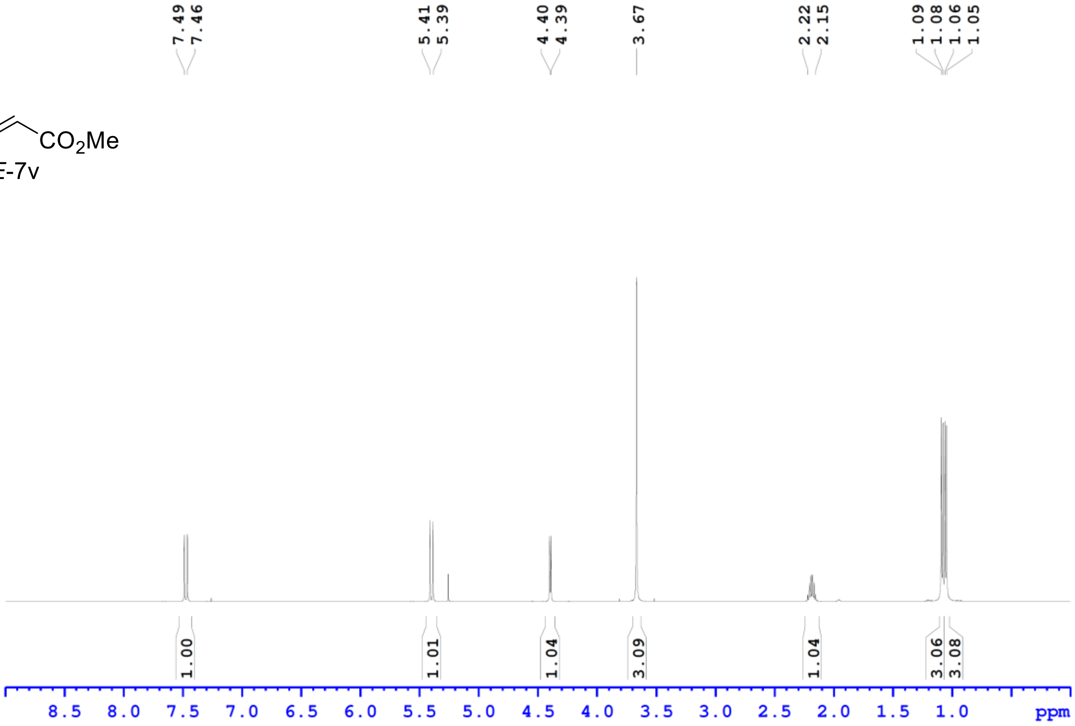
7.49
7.46
5.41
5.39
4.40
4.39
3.67
2.22
2.15
1.09
1.08
1.06
1.05
1.00
1.01
1.04
3.09
1.04
3.06
3.08
8.5 8.0 7.5 7.0 6.5 6.0 5.5 5.0 4.5 4.0 3.5 3.0 2.5 2.0 1.5 1.0 ppm

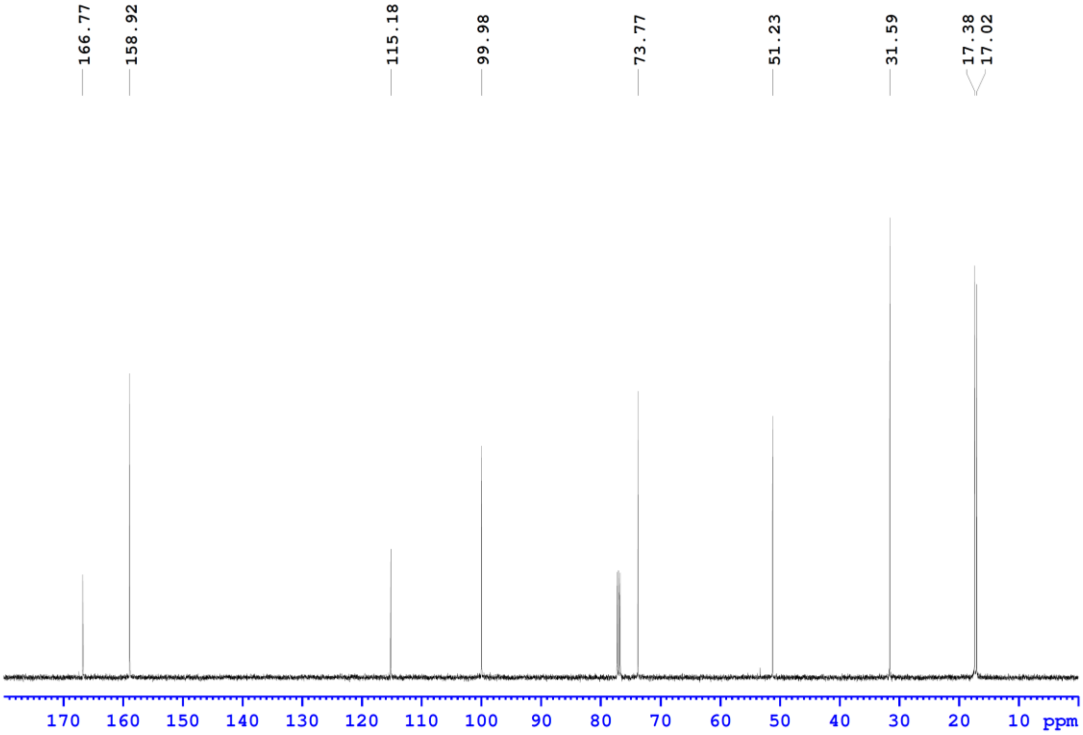
166.77
158.92
115.18
99.98
73.77
51.23
31.59
17.38
17.02
170 160 150 140 130 120 110 100 90 80 70 60 50 40 30 20 10 ppm

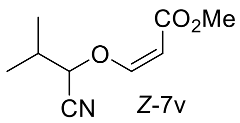

Z-7v

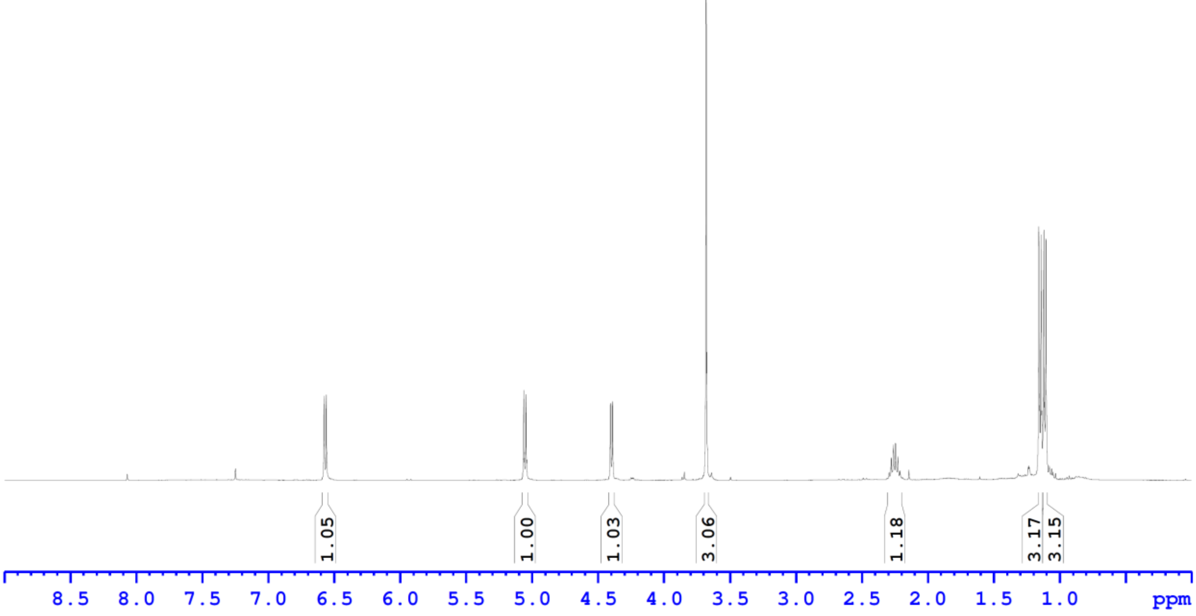

6.58
6.56
5.06
5.05
4.41
4.39
3.68
2.29
2.21
1.16
1.14
1.12
1.10
1.05
1.00
1.03
3.06
1.18
3.17
3.15
8.5 8.0 7.5 7.0 6.5 6.0 5.5 5.0 4.5 4.0 3.5 3.0 2.5 2.0 1.5 1.0 ppm

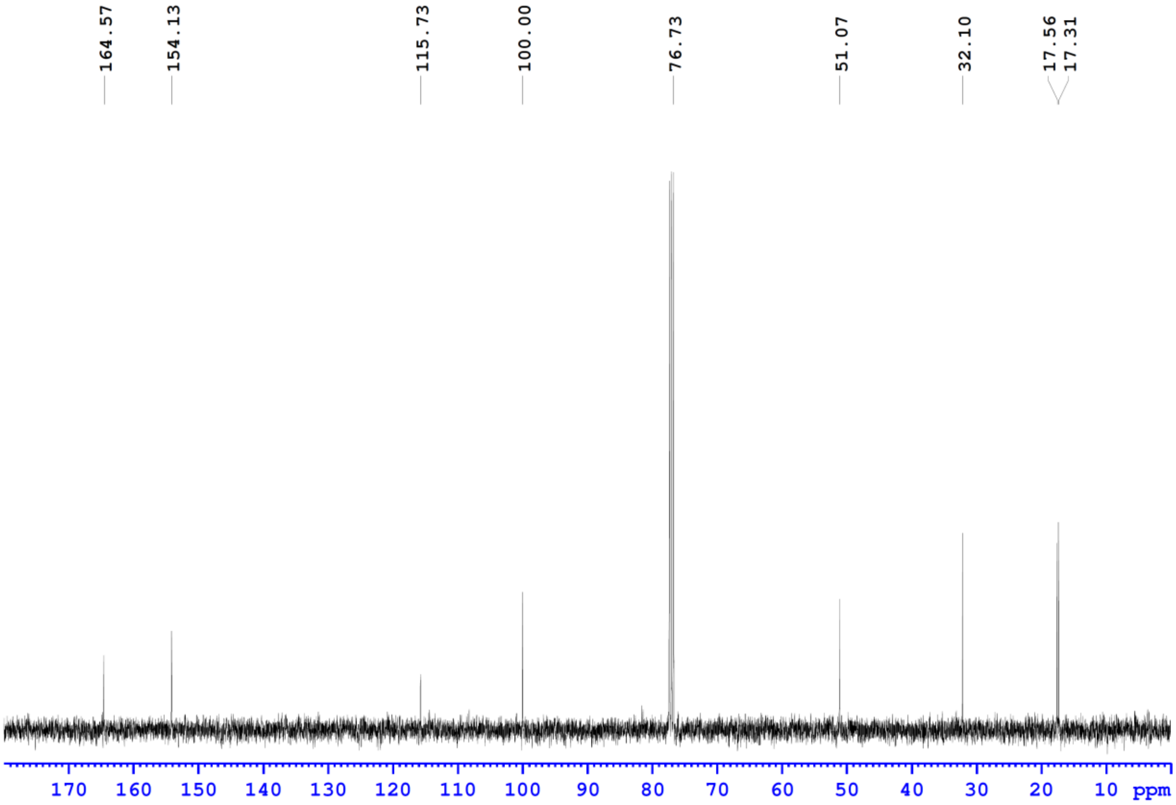

164.57
154.13
115.73
100.00
76.73
51.07
32.10
17.56
17.31
170 160 150 140 130 120 110 100 90 80 70 60 50 40 30 20 10 ppm

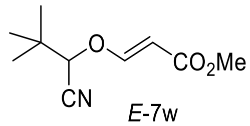
O
CO2Me
CN

E-7w

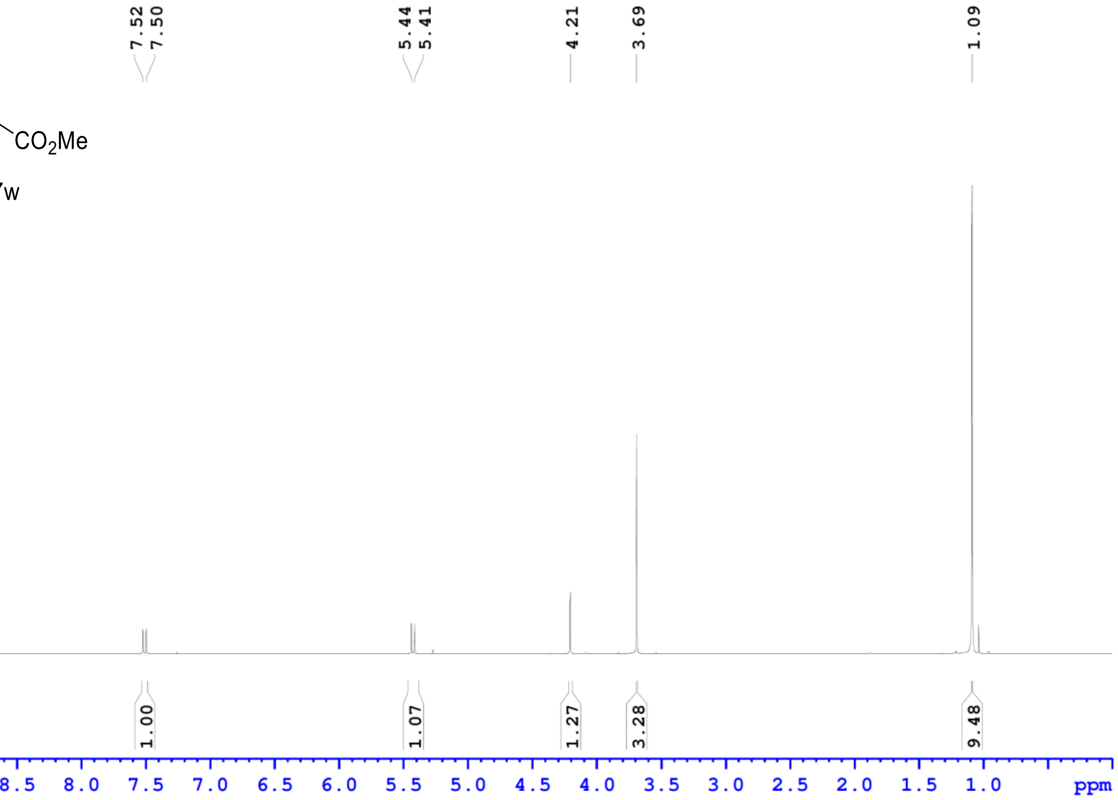
7.52
7.50
5.44
5.41
4.21
3.69
1.09
1.00
1.07
1.27
3.28
9.48
8.5 8.0 7.5 7.0 6.5 6.0 5.5 5.0 4.5 4.0 3.5 3.0 2.5 2.0 1.5 1.0 ppm

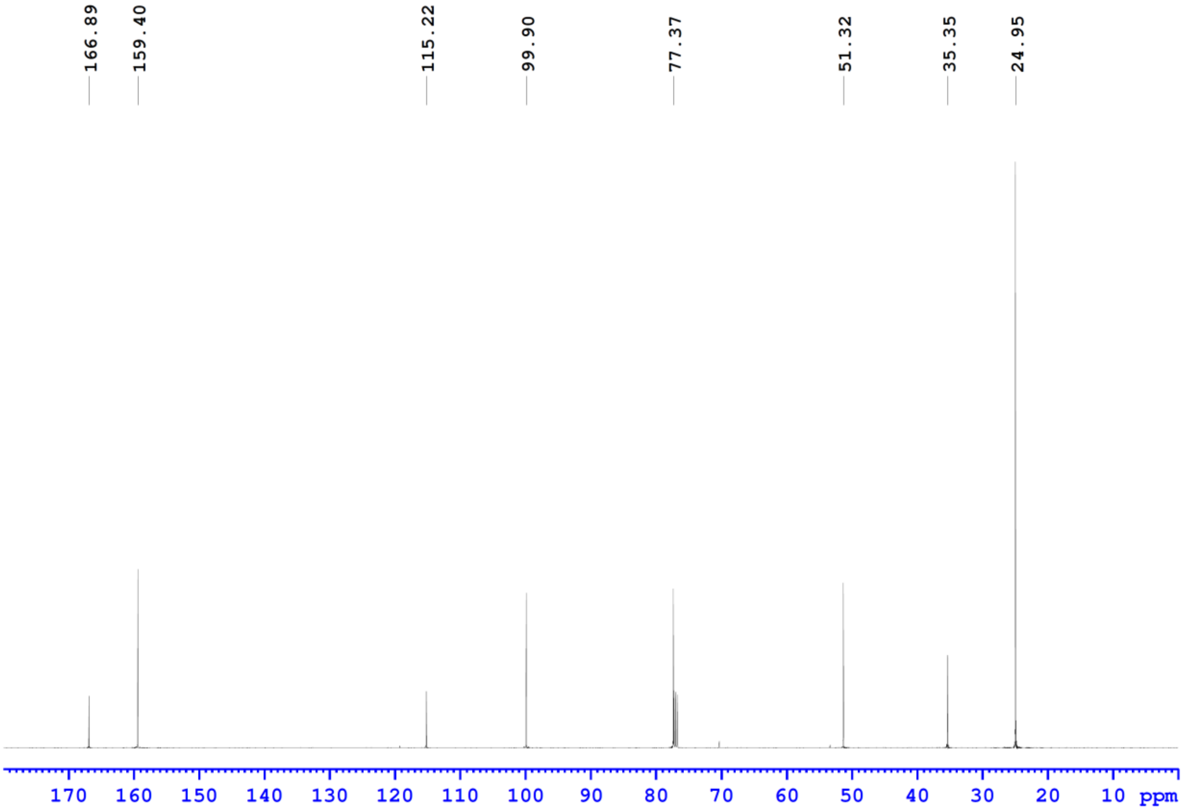
166.89
159.40
115.22
99.90
77.37
51.32
35.35
24.95
170 160 150 140 130 120 110 100 90 80 70 60 50 40 30 20 10 ppm

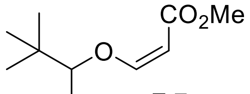
CO2Me
O
CN

Z-7w

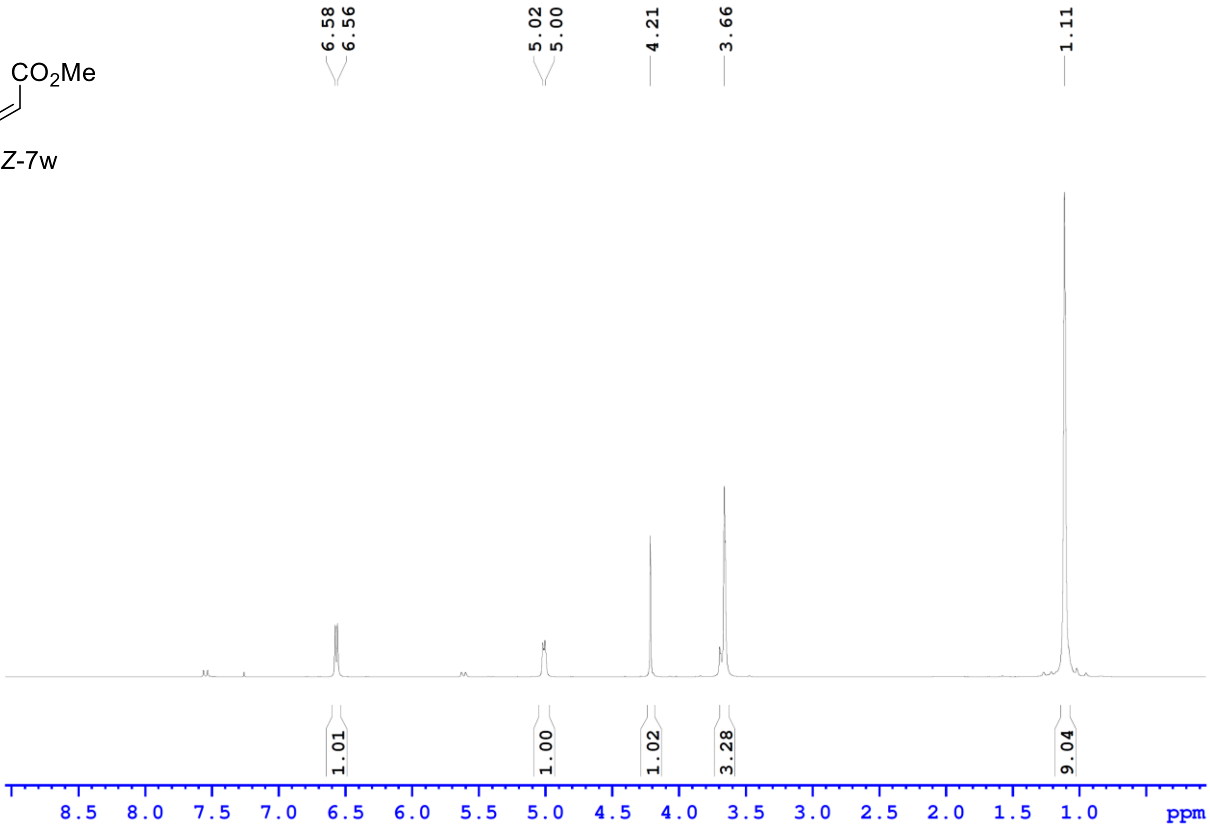
6.58
6.56
5.02
5.00
4.21
3.66
1.11
1.01
1.00
1.02
3.28
9.04
8.5 8.0 7.5 7.0 6.5 6.0 5.5 5.0 4.5 4.0 3.5 3.0 2.5 2.0 1.5 1.0 ppm

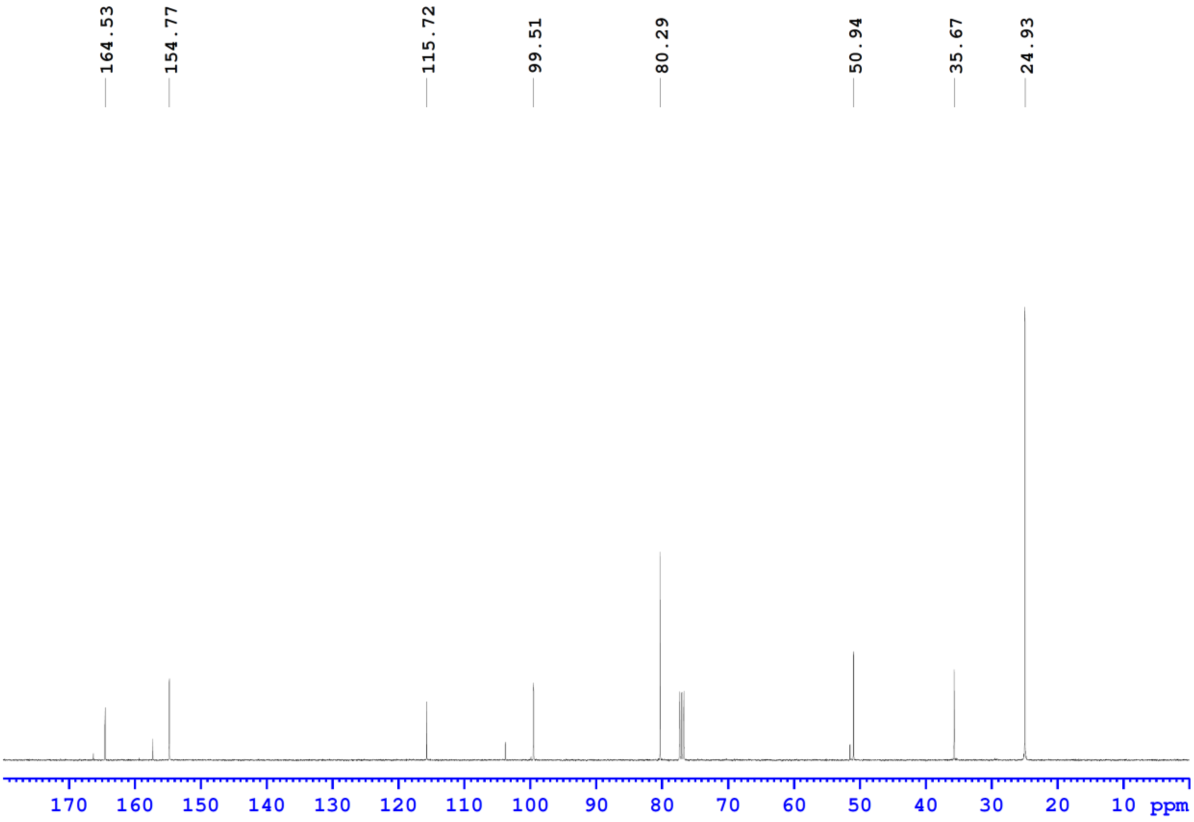
164.53
154.77
115.72
99.51
80.29
50.94
35.67
24.93
170 160 150 140 130 120 110 100 90 80 70 60 50 40 30 20 10 ppm
